# Challenges and opportunities for the characterization of electronic properties in halide perovskite solar cells

**DOI:** 10.1039/d5sc00504c

**Published:** 2025-04-29

**Authors:** Thomas Kirchartz

**Affiliations:** a IMD-3 Photovoltaics, Forschungszentrum Jülich 52425 Jülich Germany; b Faculty of Engineering and CENIDE, University of Duisburg-Essen Carl-Benz-Str. 199 47057 Duisburg Germany t.kirchartz@fz-juelich.de

## Abstract

Characterisation of the electronic properties of halide perovskites is often a dilemma for researchers. Many of the data analysis methods for the most common techniques in semiconductor device physics have a small validity window or are generally only applicable to classical doped semiconductors. As alternative data analysis approaches are often prohibitively complicated and require numerical simulations of electronic and often ionic charge carriers, the analysis of data is performed qualitatively and comparatively. The overarching idea is that even if data analysis methods do not apply to a given sample, the trend should still be maintained. However, even this last statement may not be correct in certain situations. Hence, the present review provides a summary of the canonical, frequently used methods to characterise electronic properties in halide perovskites and provides a short explanation of the pitfalls in applying the method, as well as the opportunities that arise from using these methods in ways that are not yet common in the current literature.

## Abstract 2

Finding a good match between sample, experiment and data analysis approach is challenging in halide perovskites. Solutions are presented in this perspective to facilitate this task for a wide range of material properties.

## Graphical abstract

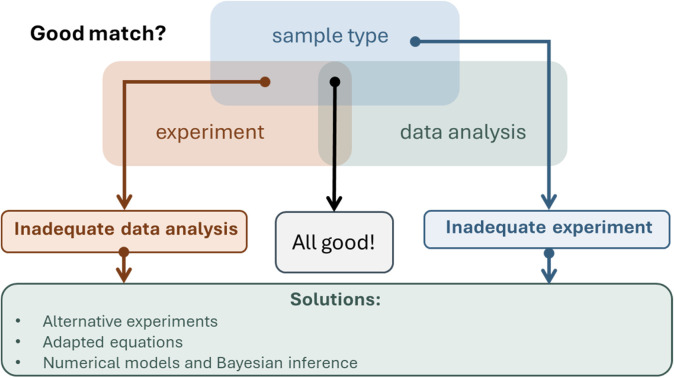

## 1. Introduction

**Fig. 1:**
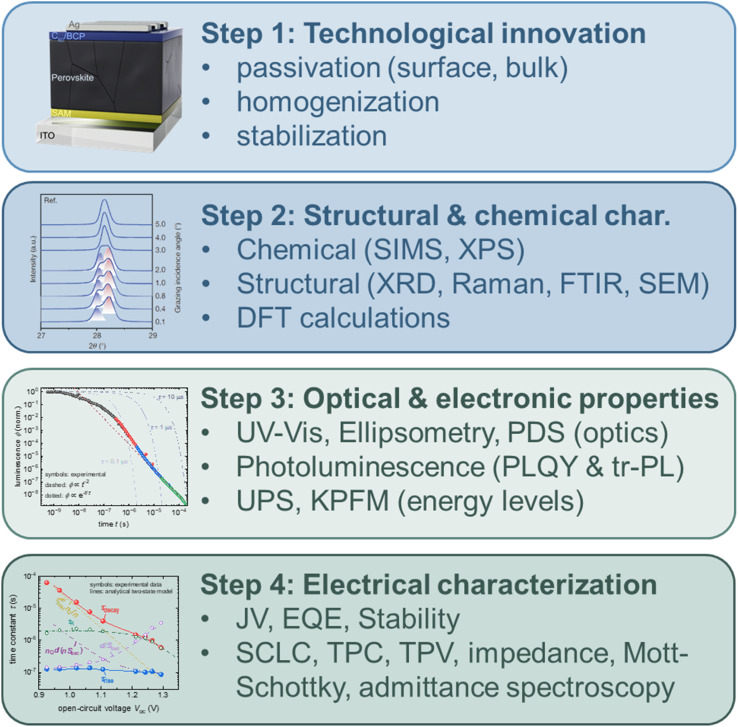
Typical flow of a primarily experimental paper that aims at technological improvements within the field of halide perovskite solar cells. Technological innovations are presented, and it is subsequently shown which structural, chemical, optical, and electronic material properties change. Eventually, the full device is characterised, which provides information on its performance and stability. Furthermore, electrical characterisation at the device level may provide further insights into properties such as defect densities, lifetimes, and interface recombination velocities. Abbreviations used: SIMS: secondary ion mass spectroscopy, XPS: X-ray photoelectron spectroscopy, XRD: X-ray diffraction, FTIR: Fourier-transform infrared spectroscopy, SEM: scanning electron microscopy, DFT: density functional theory, UV-vis: transmission and reflection measurements from the ultraviolet to the visible and often near-infrared wavelength range, PDS: photothermal deflection spectroscopy, PLQY: photoluminescence quantum efficiency, tr-PL: transient photoluminescence, UPS: ultraviolet photoelectron spectroscopy, KPFM: Kelvin-probe force microscopy, *JV*: current–voltage curves, EQE: external quantum efficiency (of a solar cell), SCLC: space charge limited current measurements, TPC & TPV: transient photocurrent & photovoltage. Figures for step 1 & step 2. Reproduced from ref. 5 under the terms of the CC-BY 4.0 license. Copyright the authors of ref. 5 (2023). Figure for step 4: reproduced from ref. 17 under the terms of the CC-BY 4.0 license. Copyright the authors of ref. 17 (2023).

A significant fraction of the work on halide perovskite solar cells is dedicated to improving the performance and stability^1–4^ of the devices by changing processes,^5–7^ modifying interfaces,^8–12^ or choosing suitable transport layers.^13–16^ As shown in Fig. 1, the improvement in solar cell performance is typically followed by an analysis of the data aimed at correlating the chemistry and structure of the materials and interfaces of the improved solar cell with electronic properties and subsequently with device performance (typically one-sun efficiency). Concerning the characterisation of electronic properties, the research community working on device optimisation has arrived at a clear consensus on the methods used to characterise each of the critical parameters shown in Fig. 1. At the same time, the community working on developing these characterisation methods and associated data interpretation methods has arrived at an entirely different consensus: many of the methods shown in the green parts of Fig. 1 are used and analysed in a way that may lead to erroneous results. Thus, the halide perovskite community is currently in a situation where classical methods of scientific quality control (*i.e.* mostly peer review) fail, such that a significant fraction of papers, including those in the most reputable journals, include data interpretation that is well understood to be wrong by the subset of the community that works on the data interpretation aspects. This problem is made more severe by the fact that in many cases “wrong” does not mean unprecise but uncorrelated to the quantity in question. In other situations, “wrong” might mean that the data interpretation may or may not be correct; it is impossible to discern from the data. In other words, confidence in the data interpretation approach is low, implying that additional information is required. Given the weak coupling between the subsets of research communities, it might be necessary to discuss known issues with the set of canonical techniques that have been established as the unwritten standard for electronic property characterisation in halide perovskites. Furthermore, I highlight possible alternatives in terms of both the techniques and data analysis methods.

### 1.1. The canonical techniques

An important question within the context of this article is how research communities arrive at a set of generally approved characterisation techniques that most researchers within the community understand and accept as both valid and useful. I will refer to these techniques within this article as canonical techniques, as they provide something akin to a research-project and paper-writing template for a significant fraction of the research community. For the religions of the book such as Judaism and Christianity, the canonical books are those that have been included in a certain edited volume of religious texts such as the bible. The selection of books that were included or excluded from a volume of religious texts was decided by a select group of people, for instance, by the Council of Rome (382 AD) for the case of the Catholic Bible. Research communities within the field of science and engineering may similarly arrive at a set of accepted wisdom, including accepted characterisation techniques that are considered both useful and sometimes mandatory for certain types of studies. Often, this set of accepted techniques that I refer to in this article as canonical techniques is not selected by groups of individuals but rather develops by interaction of researchers among each other as well as with editors and peer reviewers during the process of publishing a manuscript. Academic research can be thought of as an information network that has – in principle – excellent self-correcting mechanisms^18^ such as peer review and the possibility to write review articles, perspectives, commentaries on published papers, or discuss questions at conferences. These mechanisms should lead to continuous reassessment of the most suitable techniques for answering a given scientific question. As has been recently proven for certain techniques,^19^ these self-correcting mechanisms work relatively poorly (for the standards of academia) in the field of halide-perovskite solar cell research that is the subject of the present article.

Fig. 1 and Table 1 list a range of methods routinely used to characterise the electronic properties of halide perovskites. These techniques include the use of the trap-filled limit in single-carrier devices (often called space-charge-limited current (SCLC) measurements) and capacitance-based techniques to study defect densities. The defect densities are then correlated with the charge-carrier lifetimes and PL quantum yields, which are typically assessed by transient and steady-state photoluminescence. Often, additional time- or frequency-domain techniques are employed to determine the charge-carrier lifetimes of complete devices. These include transient photovoltage, impedance, and intensity-modulated photocurrent or photovoltage. Occasionally, charge extraction or charge transport is further characterised using methods such as transient photocurrent, transient photoluminescence on bilayers, and recently, voltage-dependent photoluminescence on complete solar cells.

**Table 1:** List of electronic properties of interest for perovskite photovoltaics connected to typically used characterisation methods and method-specific challenges and risks for data interpretation

Quantity	Method	Challenges & risks for misinterpretation
Defect density	Trap filled current in single carrier devices	Built-in voltage leads to exponential regime^20^
	Capacitance based methods	Electrode charge or injected charge dominates result^21^
Lifetime	Transient photoluminescence	Non-exponential decays interpreted with exponential model^22^
		Repetition rate affects result^23–25^
	Transient photovoltage	Capacitive effects interpreted as recombination lifetime^26^
	Impedance	One Nyquist plot at one voltage shown. Insufficient for interpretation of time constant^27,28^
Doping	Capacitance, Mott–Schottky	Missing depletion capacitance^29^
		Transition from geometric to chemical capacitance interpreted as doping density^21,29^
Energy levels	Photoelectron spectroscopy	Huge variation in values as a function of analysis method of the band edge^30,31^
Mobility	SCLC, TPC	Difficult to disentangle the effect of perovskite absorber from that of contact layers^30^
	Photoluminescence	Out-of-plane *vs.* in-plane mobilities vary by orders of magnitude^32^
Charge transfer and extraction	Transient PL on bilayers	Discrimination between recombination and charge transfer difficult^33,34^
	Voltage dependent PL	Difficult to disentangle effects of electron and hole transport layers. Complete device needed

Problems with the methods for determining defect densities are mostly based on the challenge of measuring the charge of the defects in a situation in which the charge density integrated over the thickness is much smaller than the charge density per area of the electrodes. This requirement leads to a situation in which defects can be measured *via* their charge only if their density exceeds a certain value that depends on the thickness *d* of the sample and permittivity *ε* of the material.

Challenges with determining charge-carrier lifetimes are usually associated with the difficulty in discriminating different transient effects from each other.^33,35^ Transients in films, layer stacks and devices are typically affected by many different phenomena that include charge trapping/detrapping, recombination, charge transfer and back transfer to other layers,^34^ capacitive charging/discharging of electrodes,^26,36^ and ion motion.^37–39^ Sometimes, the interpretation of the decay time as charge-carrier lifetime is possible while in other situations it is not possible or very difficult.^26^ Thus, in this context, strategies are necessary to identify confidence in the interpretation of time constants or decay times in terms of one of the previously mentioned physical phenomena.^40,41^

In this article, I will first discuss some important fundamental concepts underlying many data analysis methods for electrical and optoelectronic measurement techniques in Chapter 2. From Chapter 3 onwards, the article deals with different parameters of interest along the logic of Table 1, discusses the challenges in each chapter in more detail (last column in Table 1), and then highlights opportunities for improvement in the techniques and the respective data analysis approaches. These opportunities may be based on recent research or point towards possible directions for future innovations. The methods that I discuss will be primarily taken from the list of frequently used (*i.e.* canonical) methods. However, especially where canonical methods fall short of providing answers to the major scientific questions, I will also briefly discuss alternative techniques that may provide solutions for the problems of the more frequently used techniques. While these alternatives are not currently widely used, they may become important in the future if – as I hope – the canon of popular methods gradually shifts towards characterization and data analysis methods that maximize information gain for researchers working in the field.

## 2. Important concepts

Many concepts for the analysis of experimental data on any type of solar cell are based on the analytical approximations to the continuity equations for electrons and holes as well as the Poisson equation that will be introduced in Chapter 2.1. Analytical approximations are often possible only if one deals with a doped semiconductor, where only the continuity equation of the minority carriers is important. Thus, many of the analytical equations frequently found in the literature on halide perovskite solar cells originate from doped semiconductors, which are often inapplicable to many lead-halide perovskite compositions that frequently show a very low doping density^42^ and potentially a high density of mobile ions. The following sections allow interested readers to examine some of the concepts mentioned later in the chapters on different measurement techniques.

### 2.1. Drift-diffusion model

The importance of what I will refer to as the electronic properties of semiconductors is their impact on the transport and recombination of charge carriers in semiconducting devices. Material properties, such as the crystallinity and stoichiometry of a chemical compound, can affect transport and recombination,^43–45^ but these material properties do not directly appear in the equations used to calculate *e.g.* current–voltage curves of semiconducting devices. Furthermore, processing parameters, such as the viscosity of a solution or the annealing temperature and time, can affect the properties of the resulting device; however, they are not directly featured in the typically used equations. Instead, the electronic properties discussed in this review are properties such as mobility, lifetime, and interface recombination velocity of charge carriers as well as the defect and doping densities, work functions, and energy levels of the semiconductors involved. All these parameters appear directly or indirectly in the three equations used to calculate the carrier and current densities as a function of the external light and voltage bias. This formalism is usually referred to as the drift-diffusion model. The three coupled differential equations^46^ that are typically solved are the Poisson equation1
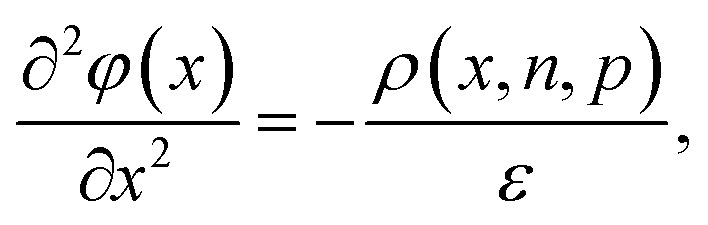
the continuity equations for electrons2
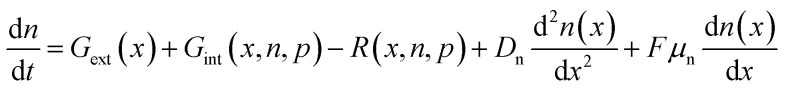
and the continuity equation for holes3
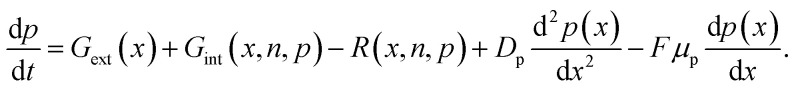
Here, *x* is one spatial coordinate normal to the surface of the film, *t* is the time, *φ* is the electrostatic potential, *ρ* is the space charge, and *ε* is the permittivity. The recombination rate, *R*, is a complex function of the charge carrier densities, *n* and *p*. The generation rate *G*_ext_ depends only on the optical properties, whereas *G*_int_ considers photon recycling, that is, the absorption of photons generated by radiative recombination. The diffusion coefficient is given by *D*_n,p_ = *μ*_n,p_*kT*/*q*, where *kT*/*q* is the thermal voltage, and *F* is the electric field.

Eqn (1)–(3) are often solved numerically using so-called drift diffusion solvers (see Table 2) and then provide a solution for *n*(*x*, *t*), *p*(*x*, *t*), and *φ*(*x*, *t*). From the carrier densities, the current densities for electrons and holes follow *via*4
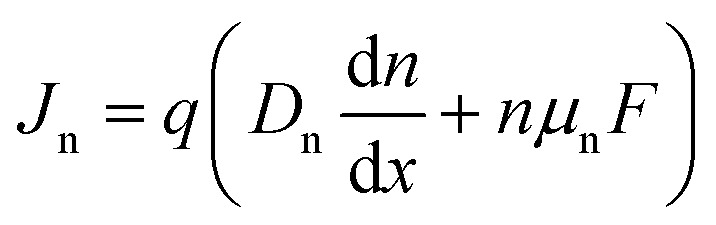
and5
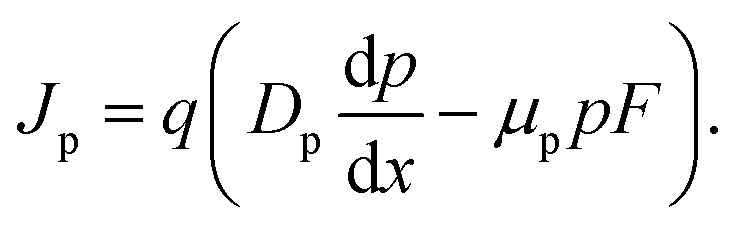


**Table 2:** Selection of drift-diffusion software tools that have found application in the context of halide perovskite solar cell simulations. The order is arbitrary, and the selection does not imply any assignment of quality by the author but is meant as a starting point for the reader to select suitable tools and inform themselves *via* the URLs provided

Name	URL	Ions included?	Transients	Frequency dependent	Free or commercial
SETFOS	https://www.fluxim.com/setfos-intro	Yes	Yes	Yes	Comm.
SIMsalabim^47^	https://github.com/kostergroup/SIMsalabim	Yes	Yes	Yes	Free
BOAR & SiMsalabim	https://github.com/i-MEET/boar	Note: Bayesian optimization tool combined with drift-diffusion tool (for fitting and parameter estimation)			
DriftFusion^48^	https://github.com/barnesgroupICL/Driftfusion	Yes	Yes	Yes	Free (Matlab based)
IonMonger^49^	https://github.com/PerovskiteSCModelling/IonMonger	Yes	Yes	Yes	Free (Matlab based)
OghmaNano^50^	https://www.oghma-nano.com/contact.html	Yes	Yes	Yes	Free
SCAPS^51,52^	https://scaps.elis.ugent.be/	No	No	Yes	Free
ASA^53,54^	https://asa.ewi.tudelft.nl/	No	No	No	Commercial

The total current density is then *J* = *J*_n_ + *J*_p_. Alternatively, in any steady state situation, the current densities can also be calculated *via* integrating over the rates of recombination and generation. In the typical sign convention where the photocurrent is negative and the recombination current is positive, we can write^55^6
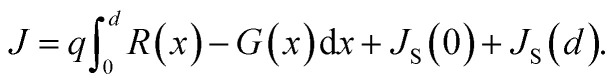
where *J*_S_ is the surface recombination current density at either integration boundary. In practice, the integration boundaries (0 and *d*) are typically chosen as the interfaces to the layers on the anode and cathode side that behave electrically as metallic, *e.g.* ITO (indium-tin oxide) and Ag. Any losses within these layers would likely be ohmic series resistances, which could be considered independently of the solution of eqn (1)–(3). The surface recombination terms consider the possibility that electrons are transported to the hole contact and *vice versa* and recombine there with the respective other carrier. As the first term on the left-hand side of eqn (6) is an integral over a finite range in the spatial coordinate *x*, the last two terms are needed to consider the boundary conditions for eqn (2) and (3) that involve the so-called surface or interface recombination velocities *S*. For more details on the boundary conditions, the effect of *S* and typical values for *S*, see for instance ref. 53 and 55–61.

### 2.2. Ionic–electronic conduction

Halide perovskites have the peculiar property that several of the possible intrinsic defects have a significant diffusion coefficient implying that the material contains charged, mobile defects.^62–65^ These mobile defects are typically referred to as mobile ions and could be for instance, halide interstitials or halide vacancies. If they are charged, any movement of these intrinsic defects by drift or diffusion will create an ionic current and may change the electrostatics of the device.^38,63,66^ Thus, ions can have a direct effect on the transient currents measured that would be absent in a purely steady-state situation. However, even in steady state, the ions can change the band diagram as opposed to a situation without ions.^67,68^ For instance, the movement of negatively charged ions towards the cathode and positively charged ions towards the anode can lead to a screening of the electric field within the perovskite absorber layer. Thus, due to this combination of ionic and electronic conduction in halide perovskites, the drift-diffusion model can be extended to accommodate also the effect of ion movement.^63,69^ Usually, ions are implemented in such a way that their charge contributes to the charge density *ρ* in eqn (1) and their movement due to drift or diffusion is added to the electronic current densities in the same way as for electrons and holes. However, there is usually no term for the generation and recombination of ions included, but their overall density is fixed, and the codes then simulate how their density changes as a function of time after some change in condition (*i.e.* a voltage step during the simulation of a current–voltage curve).

Table 2 provides a list of drift-diffusion solvers that are accessible either commercially or for free (*via e.g.* GitHub repositories) and that are either specifically designed for use with halide perovskites or have been applied to perovskite solar cells in specific situations. The table further provides information on whether the software allows one to simulate ion diffusion, transients, and frequency domain measurements. An exception is the entry for the python code BOAR which creates an interface between a Bayesian optimization algorithm and a drift-diffusion solver (in this case SIMsalabim^47^), which thereby allows finding parameters for use in a drift-diffusion code based on experimental observations (*e.g.* current–voltage curves). This implies fitting the output of a drift-diffusion solver to experimental data and thereby inferring unknown material parameters.

### 2.3. The depletion approximation

The depletion approximation is an important component in the analytical analysis of capacitance measurements and indicates that there is (a) a space-charge region (synonym: depletion region) with a certain width, such that (b) can be assumed to be depleted of free charge carriers. The charge density in the depletion region is then entirely determined by the charge of the ionised dopant atoms. Thus, if we had a p-type semiconductor that has a space-charge region towards a low-workfunction metal, the space charge in the space-charge region would be given by

7 *ρ* = *q*(*p* − *n* − *N*_A_ + *N*_D_) ≈ −*qN*_A_

where *n* is the electron and *p* is the hole density, *N*_A_ is the ionised acceptor, and *N*_D_ is the ionised donor concentration. The electric field in the space-charge region changes linearly with the position, whereas the electrostatic potential changes parabolically. This is achieved by integrating the constant space charge once (electric field) and twice (electrostatic potential) (see the discussion from page 80 onwards in ref. 70). Thus, the depletion approximation is the reason for the piecewise parabolic bands in all types of semiconductor–semiconductor and semiconductor–metal junctions.^70^ This is also the central component in deriving the Mott–Schottky method, as discussed in Section 3.2.

### 2.4. The neutral zone

In doped semiconductors, the device can usually be split into a neutral zone and one or two space charge regions. In the neutral zone, the space charge (for example for a p-type semiconductor) is

8 *ρ* = *q*(*p* − *n* − *N*_A_ + *N*_D_) ≈ *q*(*p* − *N*_A_) ≈ 0.

Hence, there is no electric field, which allows us to simplify the continuity equation within the neutral zone to the diffusion equation (see Section 2.5) to study transport and recombination. The neutral zone is central to the way Si solar cell device physics is usually explained,^71^ as in Si solar cells; typically, the space-charge region width is on the order of hundreds of nanometers, while the neutral zone has a typical thickness of 99.9% of the total wafer thickness.

### 2.5. The Debye length

The Poisson equation (eqn (1)) states that the second spatial derivative of the electrostatic potential is proportional to the space-charge density. Thus, for a given constant space charge density *ρ*, a certain distance is required for the electrostatic potential to change by a given amount. If we assume this amount to be the thermal voltage *kT*/*q*, the distance required is named the Debye length *w*_D_ and is given by (see p. 85 in ref. 70)9
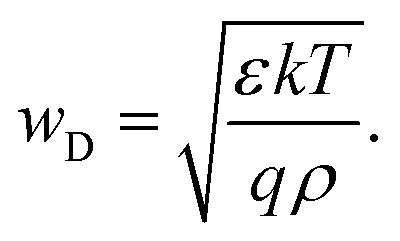


The Debye length is closely related to the width of the space-charge region which is given for the example of a p-type semiconductor–metal junction by10
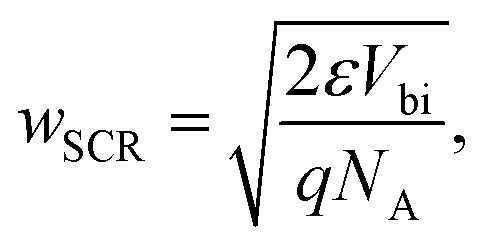
where *V*_bi_ is the built-in electrostatic potential difference of the space-charge region and *N*_A_ is the ionised acceptor density. Eqn (9) uses the depletion approximation described in Section 2.1. The significance of eqn (9) and (10) is that they allow us to estimate whether the charge density can be measured using a certain method and a certain sample. If the sample is significantly thicker than the Debye length, it will have a space-charge region and a neutral region; hence, many methods, such as Mott–Schottky, are likely to be applicable. If the sample is not thicker than the Debye length, the methods will not be applicable. Based on this logic, a detection threshold^72^ (the minimum value for the charge density) can be derived for different methods to determine the defect and doping densities (see Chapter 3.1).

### 2.6. The charge-carrier lifetime

In physics, many decay processes can be approximated as following the solution to a first order differential equation of the form d*n*/d*t* = −*n*/*τ*, where *n* is the decaying species, *t* is the time and *τ* is a characteristic time constant. The solution to such an equation is an exponential decay of the form *n*(*t*) = *n*(0)exp(−*t*/*τ*). Usually, the parameter *τ* of these decays has important implications that may range from how well suited a semiconductor is for photovoltaics up to how long one must store radioactive waste. The parameter *τ* has many names that include decay time, characteristic time constant or lifetime of the species *n*. For processes that do indeed follow d*n*/d*t* = −*n*/*τ* to a good approximation, the lifetime is extremely important, useful, and straightforward to understand. Once processes do decay but may not exactly (or even not at all) follow d*n*/d*t* = −*n*/*τ*, the use and interpretation of the lifetime becomes a challenge of central relevance also to the field of halide perovskites.

Within the field of semiconductors, the charge-carrier lifetime is a concept for quantifying the speed of recombination that makes sense in doped semiconductors and semiconductors with deep traps.^73^ In both cases, decay processes follow d*n*/d*t* = −*n*/*τ* quite well. Either of the two conditions being fulfilled (doping or deep traps) is a highly relevant scenario for all traditional semiconductors. Thus, the charge-carrier lifetime is nearly omnipresent in the literature on semiconductors. However, this is not an easy-to-use concept for intrinsic semiconductors with shallow traps or band tails, which is often relevant for both lead-halide perovskites and organic semiconductors. To understand the charge-carrier lifetime, we must distinguish between models and quantities derived from experimental data, both of which are often called lifetimes but are not necessarily the same.

### 2.7. The diffusion length

In the neutral zone of a solar cell based on doped absorber layers, we have a negligibly small electric field, and majority and minority carriers. Thus, in the example of a p-type semiconductor, in the neutral zone, electrons are minority carriers, and only the recombination and transport of electrons affect the open-circuit voltage *V*_oc_ and short-circuit current density *J*_sc_. The slow transport of majority carriers can cause resistive loss, reducing the fill factor, FF, but it cannot lead to recombination losses. In the neutral zone, eqn (2) simplifies to11
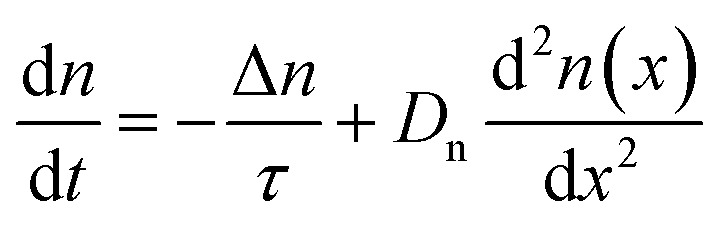
which in steady state (d*n*/d*t* = 0) has the solution12
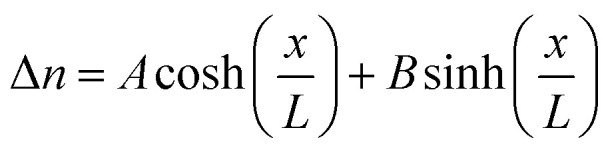
where 
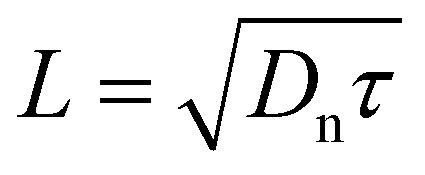
 must hold to satisfy eqn (11). Parameters *A* and *B* must be determined using appropriate boundary conditions but are not important for the current discussion. The important aspect of eqn (12) is that only the square root of the product of the diffusion coefficient and lifetime controls the spatial dependence of the excess electron concentration and thereby the recombination rate *R* = Δ*n*/*τ*. All other parameters that could affect recombination are part of the boundary conditions. Thus, it is a logical concept to use the diffusion length *L* as a figure of merit for the bulk electronic quality of a doped (!) semiconductor.

## 3. Defect characterization

Quantifying the densities and energetic or spatial positions of defects is a useful intermediate step in relating the effect of bulk or interface passivation strategies to recombination rates and, eventually, functionality. To measure defects, one requires an observable that serves as a proxy for the actual defect density. The most frequently used proxy for defect density is the bulk charge density^72^ which is unrelated to the charge of free carriers. If such a charge density can be unambiguously identified, values of defect densities can be inferred at least approximately. Alternatively, defects can be inferred from photoemission signals; that is, if a UV photon can create a free electron above the vacuum level from a certain energetic depth, then one can infer the presence of a near-surface state at a certain energy.^16,31^ As long as this occurs at an energy within the bandgap, defects near the surface can be identified in such a way.

### 3.1. *JV*-Curves of single carrier devices

#### 3.1.1 Introduction

**Fig. 2:**
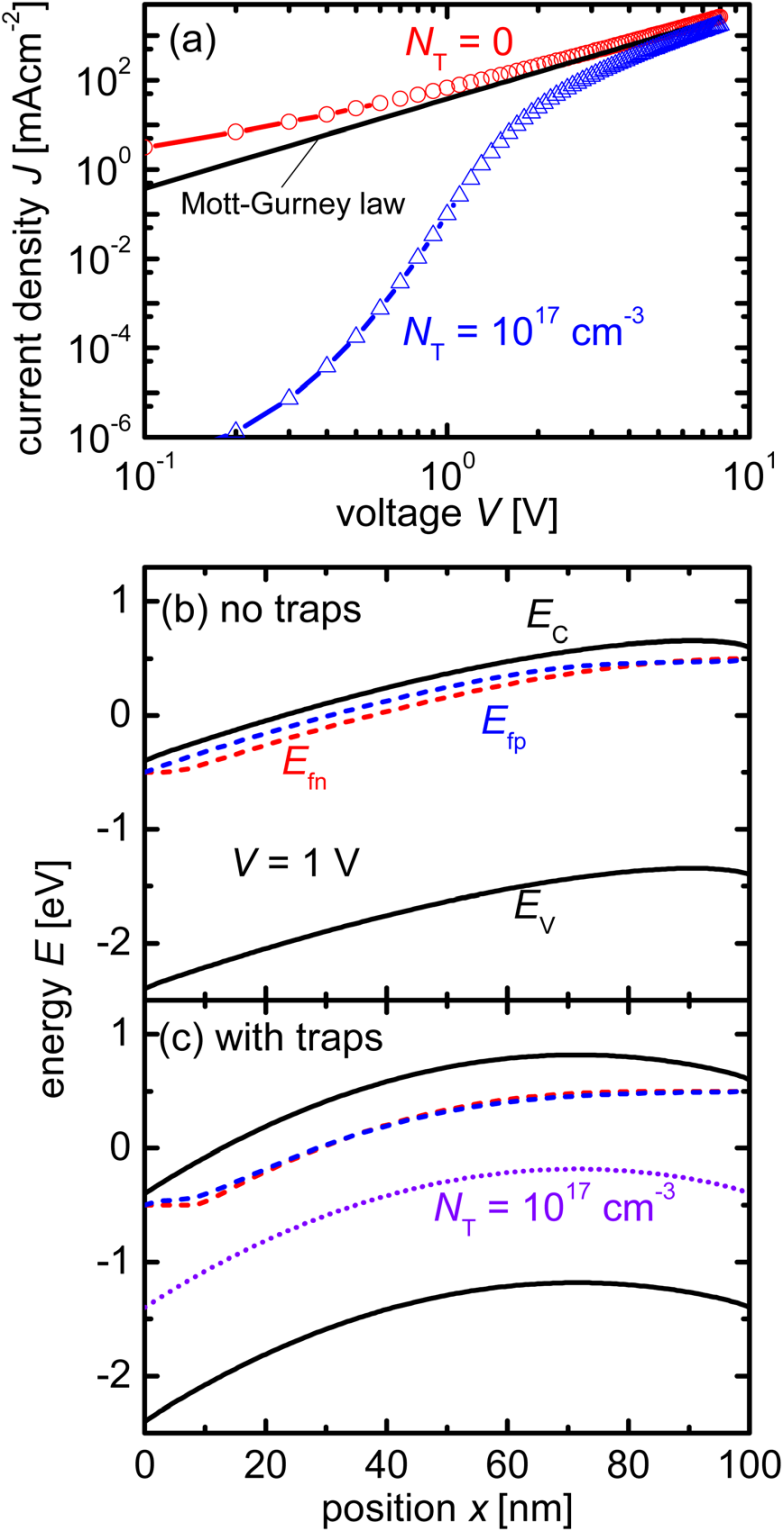
(a) Current/voltage curves of a device with and without charged acceptor-like defects with a total concentration of *N*_T_ = 10^17^ cm^−3^ and a Gaussian width of *σ* = 100 meV are compared to the Mott–Gurney law. Band diagrams of device (b) without charged defects and device (c) with charged defects. The acceptor-like defects in (c) create a barrier owing to their negative charge. The diffusion of electrons up the barrier causes a reduction in the current in (a). Figure reproduced from ref. 20 under the terms of the CC-BY 2.0 license. ©2013. The author of ref. 20.

**Fig. 3:**
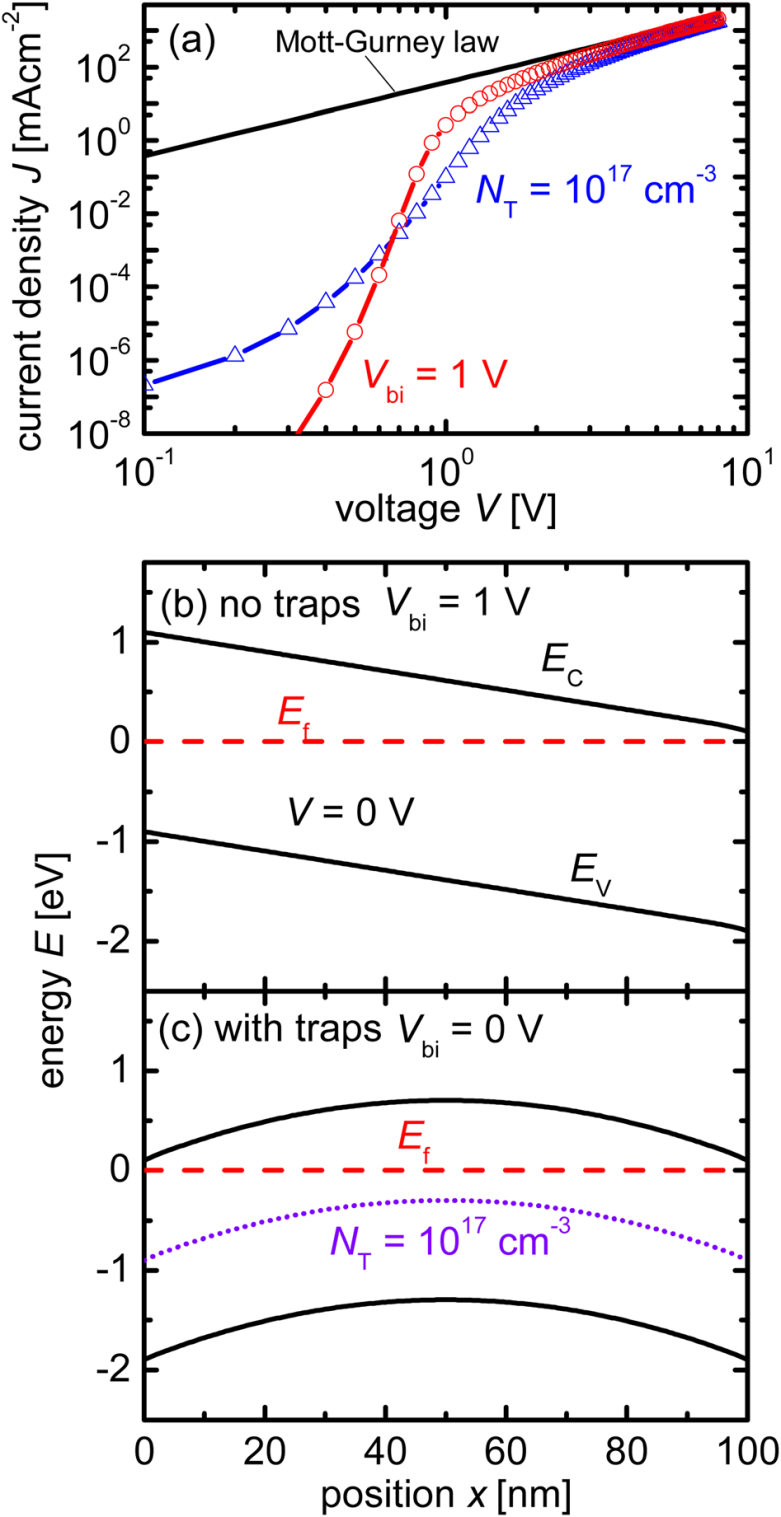
(a) Current/voltage curves of a device with charged acceptor-like defects and a built-in voltage of *V*_bi_ = 0 V (as in Fig. 1a), and a device with no defects but a built-in voltage of*V*_bi_ = 1 V are compared to the Mott–Gurney law. Band diagram of device (b) without charged defects and *V*_bi_ = 1 V, and (c) with charged defects and *V*_bi_ = 0 V. Both band diagrams are depicted at short circuit. Figure reproduced from ref. 20 under the terms of the CC-BY 2.0 license. © 2013. The author of ref. 20.

Measuring the current–voltage (*JV*) curves of single-carrier devices is a popular technique in highly resistive materials used for semiconductor applications. The original physics behind these techniques originates from research fields that have studied current injection into insulators.^74^ Subsequently, these techniques are frequently used in the characterisation of organic semiconductors, where mostly mobilities were measured using the Mott–Gurney law.^75,76^ Here, the key advantage of the method is that at certain forward voltages of usually >1 V, the current scales quadratically with voltage with the prefactor only depending on mobility, thickness, and permittivity (see Fig. 2a).^77,78^ As it is usually possible to fabricate both electron and hole-only devices, this is one of the few methods to determine electron and hole mobilities separately in a poorly conductive material, where the use of the Hall effect is usually difficult to impossible.^79^ At lower voltages, the current may scale linearly or exponentially with voltage (see Fig. 2a and 3a).^77^ The linear behaviour is seen for intrinsic, defect-free single-carrier devices with a built-in voltage of zero. The latter implies that the work-function difference between the two contacts is zero (see Fig. 2b). If one of these conditions is not met, exponential behaviour is observed.^20,80^ Usually, the exponential relationship between the current and voltage is due to the diffusion of carriers over an electrostatic barrier (see Fig. 2c and 3c). The easiest type of electrostatic barrier is a built-in voltage that creates an electric field that impedes the flow of majority carriers (see Fig. 3b and c).^80^ This is similar to the design of any rectifying diode, including solar cells, where under forward bias, charge carriers have to diffuse against the electric field of a pn-junction, pin-junction, or two electrodes with different work functions. A second option would be the existence of charged defects (see Fig. 2c), whereby the charge has the same polarity as the majority carriers.^81^ Thus, if there are negatively charged defects in an electron-only device, the electrons will have to diffuse against the electric field created by this charge density before they can reach the other electrode. In an ideal case, where no other effects such as injection barriers modify the current–voltage curve, the voltage at which the exponential trap-filled current transitions into the quadratically voltage dependent space-charge-limited current can be used to determine the trap density using *V*_tfl_ = *qN*_def_*d*^2^/(2*ε*_0_*ε*_r_). As this equation is easily solved for the defect density *N*_def_ it would be an attractive and simple way to determine the defect density if all underlying assumptions were met.

#### 3.1.2 Challenge

**Fig. 4:**
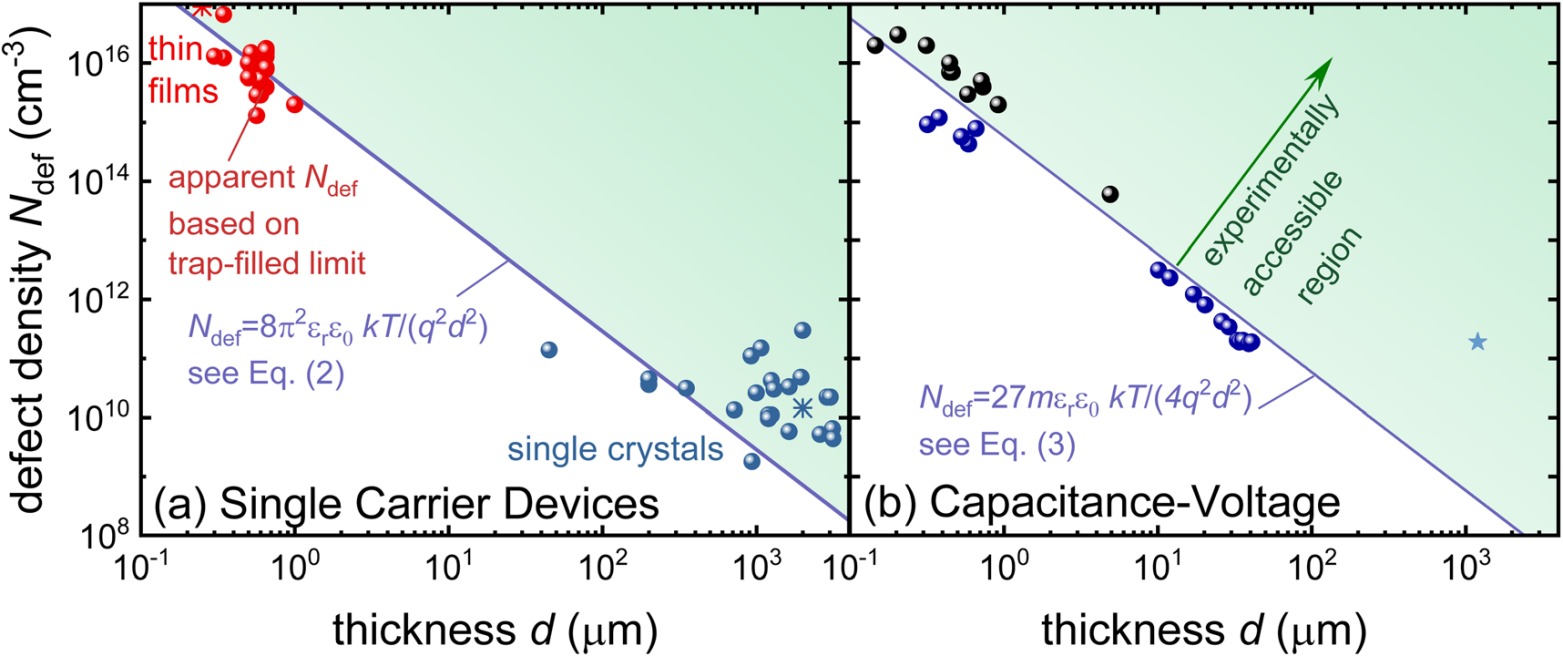
Apparent defect densities *vs.* thickness for (a) single-carrier devices and (b) capacitance–voltage curves of perovskite solar cells (black symbols) and drive-level capacitance profiling data (blue symbols). The lines indicate the minimum volume defect density that should be measurable according to the equations in the figure. Reprinted with permission from ref. 72. © 2021. The authors of ref. 72.

An overwhelming number of studies in the field of halide perovskites deal with defect passivation; hence, measuring the successfully reduced density of defects is crucial for maintaining the narrative of the paper. Measuring the exponential part of the current–voltage curve of single-carrier devices is extremely simple and hence a very attractive method to support the conclusions of many experimental studies. However, for the bulk charge density *qN*_def_ to be detectable, it must be at least on the same order of magnitude as the surface charge density on the electrodes. In a constant field approximation, the surface charge density *σ* is given by *σ* = *ε*_0_*ε*_r_Δ*φ*/*d*, where *d* is the thickness between the plates, *ε*_0_ and *ε*_r_ are the vacuum and relative permittivity, and Δ*φ* is the electrostatic potential difference between the two electrodes. Hence, we obtain the relation *N*_def_ ≫ *ε*_0_*ε*_r_Δ*φ*/(*qd*^2^) from these simple arguments. Based on numerical simulations performed in ref. 72, we obtain *N*_def_ > 8π^2^*ε*_0_*ε*_r_*kT*/(*q*^2^*d*^2^) as a more accurate threshold for our ability to detect bulk charge densities in a sandwich type device structure. Regardless of the exact equation, the detection threshold for detecting defects with single-carrier device measurements shows a permittivity/thickness^2^-type behaviour, which implies that the thinner the film, the higher the defect density must be to be detectable. As shown in the meta-analysis of the literature data provided in Fig. 4a, halide perovskite thin films generally do not exhibit sufficient bulk charge density to uniquely identify the defect density. Thus, the blue line in Fig. 4a must be regarded as the upper limit of the defect density.

#### 3.1.3 Opportunity

The frequent use of the trap-filled limit in analysing trap densities in halide perovskites has triggered a small but still relevant number of studies aiming at improving the measurement and interpretation of the method in such a way that it becomes more useful for the halide perovskite community. Among the first innovations was the pulsed measurement technique proposed by Duijnstee *et al.* that was developed primarily in response to ionic effects.^82^ Ion movement can lead to hysteresis in the *JV* curves that can be so significant that data analysis even in thick single crystals that are unaffected by the detection threshold issue discussed above becomes difficult or impossible. The solution to this problem is the so-called pulsed-voltage SCLC measurement.^82^ The idea is to combine short measurement times (20 ms for the single crystal samples under investigation) and combine that with long rest times (7 min) between individual measurement points at different voltages. This approach leads to negligible hysteresis in the resulting *JV* curves but of course dramatically increases the overall measurement time.

A second noteworthy insight^83^ was the investigation into the correct feature of the *JV* curve that should be used to assign the trap-filled voltage to, once the detection-threshold check is positive. Thus, for applications in single crystals, where the sample thickness is high enough for the trap detection method to fundamentally work, there is still the question of how to reliably and accurately determine the trap-filled voltage *V*_tfl_. Often, the first kink (at the lowest voltages) is used in literature. This first kink separates the linear part of the *JV* curve to the exponentially increasing one that is a signature of the actual trap filling process. The second kink is then the transition from the trap-filled current to the space-charge limited current (*J*–*V*^2^), which is indeed the point that does correlate with the trap density. This, however, means that even for single crystals, the trap density determination requires three regimes to be clearly visible (linear, exponential and quadratic) as only the transition exponential to quadratic contains the information on the overall trap density. For a visual illustration of this message, see for instance the table of contents figure of ref. 19. Further, it is noteworthy that 3 years after the 2021 publication of ref. 83 still 80% of the papers citing (!) ref. 83 use the lower kink in the curve and only 20% use the correct upper kink.^19^ This means that even though the information is in the literature, and the papers are found and cited, they are still having a moderately low influence on the actual behaviour of the researchers performing and analysing the measurements.

Instead of measuring the defect density, it might be safer to use the measurement of single-carrier devices to map the energy levels and work functions of contacts by comparing forward and reverse bias measurements. In an ideal single-carrier device, both injection barriers are zero. However, the injection barriers are often slightly different, which leads to differences in the current–voltage curves for forward and reverse bias. Röhr recently developed an analytical approach to quantify the electrostatic potential difference between the two electrodes of a single carrier device from the current ratio between forward and reverse bias sweeps.^80^ As small differences in the injection barriers are visible in the current ratios, the method can be applied to measure the work function differences between different contact layers.^30^ While the absolute work function is not accessible, the differences are, and these are usually the relevant quantities needed for numerical simulations of devices.

**Fig. 5:**
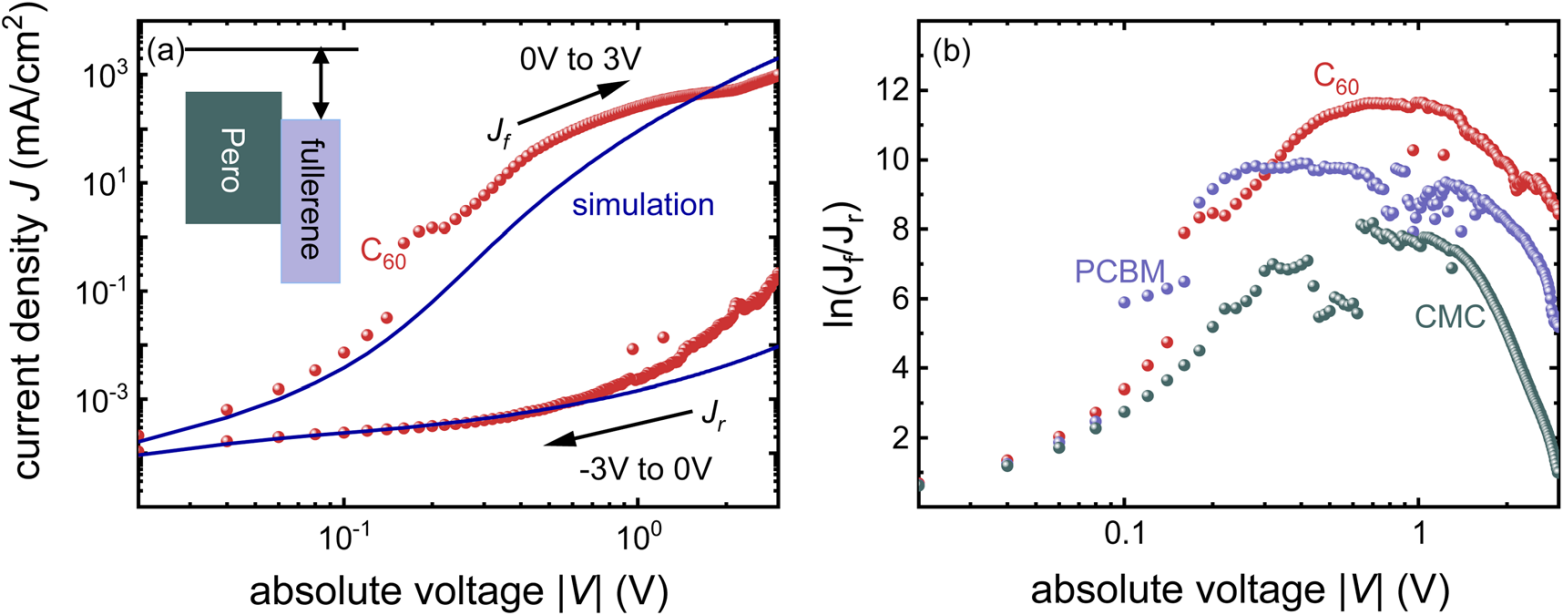
(a) Dark *JV*-curves of the electron-only device with C_60_ as electron transport layer (spheres) and drift-diffusion simulations (solid lines). The lower branch with the arrow to the left is the measurement/simulation from −3 V to 0 V (reverse current *J*_r_), and the upper branch with the arrow to the right represents the measurement/simulation from 0 V to 3 V (forward current *J*_f_). (b) Logarithm of the ratio *J*_f_/*J*_r_ calculated from measurements of electron-only devices with different fullerenes (spheres). Redrawn from ref. 30 under the terms of the CC-BY 4.0 license. © 2023. The authors of ref. 30.

Fig. 5 shows an example of how the choice of electron injection layer affects the *JV* curves of single-carrier devices. Fig. 5a shows the forward and reverse bias measurements of the glass/ITO/SnO_2_/perovskite/C_60_/BCP/Ag device. Fig. 5b shows the experimental ratio between the forward and reverse current densities for different fullerenes with slightly different electron affinities. As fullerenes with higher electron affinities and a larger conduction band offset to the perovskite show higher current ratios, we presume that there is Fermi level pinning at the BCP-fullerene interfaces that leads to higher built-in potential differences in those cases, where the electron affinity is high.

### 3.2. Capacitance-based techniques

#### 3.2.1 Introduction

The Mott–Schottky method is a classical approach for determining the doping densities in Schottky junctions or pn-junctions. The width of a space-charge region has a square-root-like voltage dependence, and the width determines the size of the capacitance of the space-charge region.^84^ As the doping density is a prefactor of the relation between capacitance and voltage, it can be determined provided only the knowledge of the relative permittivity of the material. As charged defects are not conceptionally different from ionised dopants, it is possible to measure the defect densities by measuring their impact on the capacitance of a device.

#### 3.2.2 Challenge

**Fig. 6:**
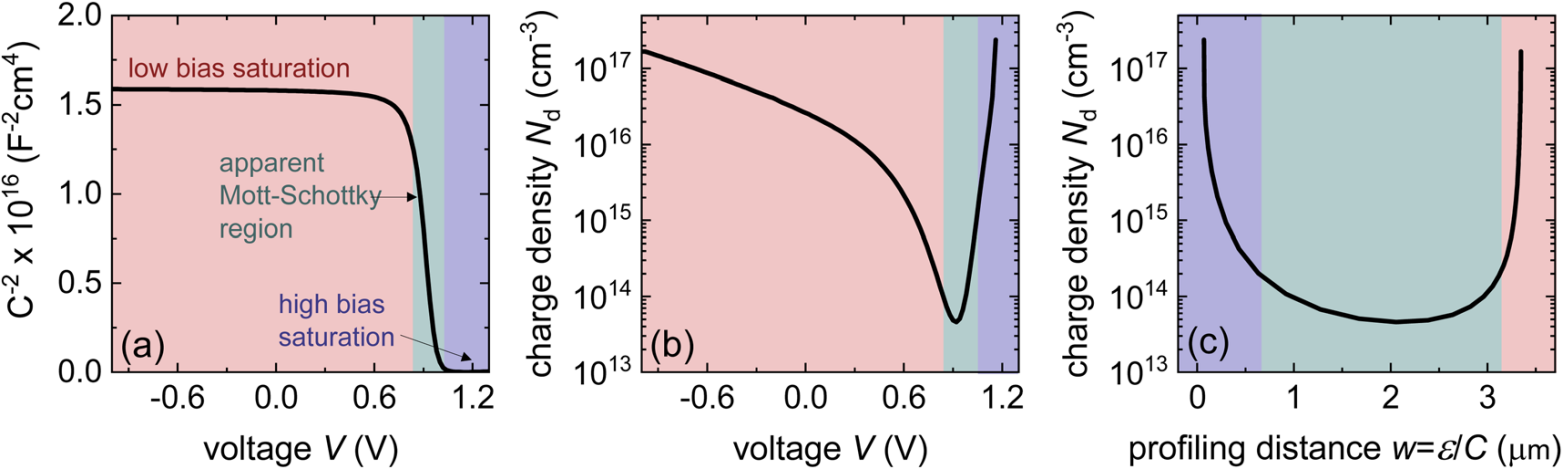
(a) Simulated Mott–Schottky plot of an intrinsic perovskite solar cell that shows a transition from a voltage-independent geometrical capacitance at reverse and low forward bias (red) to a higher capacitance at forward bias owing to charge injection (blue). The transition region (green) is frequently misinterpreted as a region indicative of the depletion capacitance, and hence, the doping density. (b) Apparent charge density resulting from the slope of the curve in (a) plotted as a function of voltage. (c) Apparent charge density resulting from the slope of the curve in (a) plotted as a function of x position. The figure was reproduced with permission from ref. 21. © The American Association for the Advancement of Science, 2021.

The key prerequisite for the above-mentioned approach is that the sample has a space-charge region that depends on the volume charge density.^84^ If the device for instance is fully depleted or has a capacitance *C* that is dominated by other types of capacitances (*e.g.* chemical or geometrical capacitances), the determination of the defect or doping density is impossible.^29^Fig. 6 explains the general features of a Mott–Schottky analysis of an undoped and defect free sample. Fig. 6a shows the Mott–Schottky plot, where *C*^−2^ is plotted *vs.* voltage *V*. At reverse and low forward bias (red region), the capacitance saturates to a constant value given by the geometrical capacitance. Towards forward bias (approximately 0.6 V), the value of *C*^−2^ decreases to (seemingly) zero. During this drop, the curve has an inflection point (within the green region), which is frequently used to fit a straight line to the data. Alternatively, the derivative of the curve in (a) is used to calculate the apparent charge density, which is later interpreted based on contextual knowledge about the sample as either doping or defect density. The data could initially be plotted as a function of voltage, as shown in Fig. 6b. Subsequently, the apparent charge density decreases slightly during the saturation part to achieve a sharp minimum at the inflection point of the curve in panel (a). At higher voltages, the apparent charge density increases again. Note again that the *y*-axis in panel (b) is the charge density determined by an inapplicable equation from the simulation in panel (a) and is entirely wrong, as the simulated sample is completely defect- and doping-free. Fig. 6c shows a plot that is frequently used in the perovskite community to present a depth-dependent defect density. The logic of the plot is that in a partially depleted sample, each voltage represents a certain position on the edge of the space charge region, which should dominate the signal.^84,85^ In the present situation of the undoped and defect-free sample with a fully depleted absorber layer, both the *y*-axis and *x*-axis are determined by inapplicable equations, however. The final U-shaped result is^29^ dangerously attractive^21,86,87^ as it apparently shows a low and quite constant bulk defect density (green region) and a higher defect density towards the interfaces. This complies with expectations (confirmation bias) but constitutes a misinterpretation of the data due to the inapplicability of the assumptions required for the data analysis approach. The simulated sample had neither bulk nor interfacial defects. The blue and red regions that apparently show a high defect density towards the interfaces result from the geometrical capacitance (red) and a capacitance related to charge injection (blue).

**Fig. 7:**
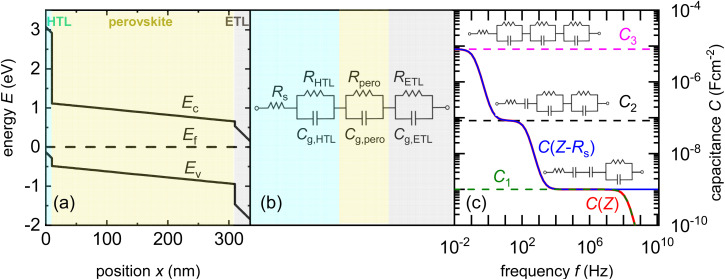
(a) Band diagram of a perovskite solar cell with the hole transport layer (HTL) and electron transport layer (ETL) highlighted. (b) AC equivalent circuit of the layer stack in (a), whereby each of the resistances except *R*_s_ must be understood as being inherently voltage dependent. (c) Frequency-dependent capacitance based on the equivalent circuit shown in (b), whereby each plateau shown corresponds to one of the resistances in the equivalent circuit being negligible relative to the value of 1/(*jωC*) of the respective R‖C element. Figure reproduced from ref. 88 under the terms of the CC-BY 4.0 license. © 2022. The authors of ref. 88.

A second challenge in understanding the shape of the capacitance–voltage curves is the debated origin of the forward bias capacitance, which leads to the inflection point in Fig. 6a and the U-shape in Fig. 6c. There are several reasons why the capacitance can increase at a forward bias. The implicitly assumed reason in the logic of the Mott–Schottky analysis is the change in the width of the space-charge region with voltage. This change should happen, however already at reverse bias and the absence of any significant slope until short circuit is a clear indicator that this is not the reason. The second option is the so-called chemical capacitance, which originates from charge-carrier injection into the absorber. A third and often overlooked reason is that perovskite solar cells consist of several layers, as shown in Fig. 7a and b, each containing a capacitance and differential resistance at every bias point. While the resistances of the contact and transport layers might be ohmic (or not), the differential resistance of the actual absorber layer, including its interfaces, must be exponential over a significant voltage range in forward bias in any good solar cell. Thus, even if we assume a constant (*e.g.* geometric) capacitance in any individual layer, by the simple fact that one of the resistances scales exponentially with voltage, the total measured capacitance also starts to have steps both in voltage and frequency (as shown in Fig. 7c). Thus, the simple idea of a series connection of several RC elements, as shown in Fig. 7b, is sufficient to lead to a capacitance step, such as that shown in Fig. 7c. The significance of the position of this step on the voltage axis is related to the dark *JV* curve and open-circuit voltage. As many researchers have correlated the position of the inflexion point with the built-in electrostatic potential difference *V*_bi_ of the solar cell and then, in turn, with *V*_oc_, one often observes correlations between the two parameters. Thus, this method is promoted by an unconscious confirmation bias. The method confirms what researchers intuitively expect and is, therefore, popular. From a conceptual point of view, the key problem is the mix of causality and correlation. The onset of the *C*(*V*) measurements correlates with parameters such as *V*_oc_, but they are not the cause but rather the effect. Instead of a change in *V*_bi_ causing a higher *V*_oc_, the data could also be explained by improved bulk or interface passivation leading to a higher *V*_oc_, thereby lowering the saturation current density *J*_0_ and the *C*(*V*) step at a higher forward voltage.

#### 3.2.3 Opportunity

Based on our current level of understanding, the classical Mott–Schottky method is only useful for confirming that a certain perovskite composition is sufficiently intrinsic that the Debye length is larger than the thickness of the perovskite layer. We are not currently aware of any successful attempts to measure the doping density of halide perovskite thin films using the Mott–Schottky method. Even in single crystals, only the thickest ones seem to show a behaviour that is indicative of a doped semiconductor with an extremely low doping density.^86^ However, there are cases in which a clear change in the Mott–Schottky plot in thin films could be observed after pre-biasing the sample,^89^ which shows that the properties and distribution of ions influence the measurement. Furthermore, Diekmann *et al.* studied the possibility to quantify mobile ion densities from low-frequency Mott–Schottky methods. Unlike in the case of higher frequencies, where electronic processes dominate, the Mott–Schottky plot at low frequencies (<5 Hz in the case of ref. 90) can approximately reproduce the input values of the ion densities used for the simulations if the ion densities exceed around 10^16^ cm^−3^ for an assumed perovskite thickness of 500 nm. Note that this finding is consistent with the detection threshold shown in Fig. 4. In addition, also experimental data shows an approximately linear behaviour that does not have the typical shape as seen in Fig. 6a that is consistent with an intrinsic semiconductor. The biggest source of error in the determination of the ion density *via* Mott–Schottky originates from the electrostatic potential drop over the electron and hole transport layers. They can lead to an underestimation of high ion densities, whereby the error increases up to an order of magnitude for ion densities of 10^18^ cm^−3^. This in turn is consistent with the challenges discussed in the context of Fig. 7. The ability to determine the ion density, in turn also allows the determination of ion diffusion coefficients *via* a combination of a Mott–Schottky like analysis of the ion density combined with a charge-extraction measurement that is sensitive to the overall ionic conductivity (product of mobility and density of ions) which in turn allows the extraction of the ion mobility or diffusion coefficients as shown by Diethelm *et al.*^91^

### 3.3. Pump-push photocurrent

#### 3.3.1 Introduction

The discussion within Chapter 3 was so far centred around frequently used methods that share the common problem that they measure defect densities *via* the indirect effect of the charge densities of defects on observables such as the current flow or the capacitance (*i.e.* the out of phase current flow). The downside of all these techniques is that they do not supply a measure to suppress other sources of charge such as the surface charge density present on electrodes of different workfunction or different electrostatic potential that is essentially always present in solar cells. A logical approach to investigate defects more directly is therefore to find a way to selectively excite the defects in such a way that the signal of the defects can be isolated from other effects.

#### 3.3.2 Challenge

**Fig. 8:**
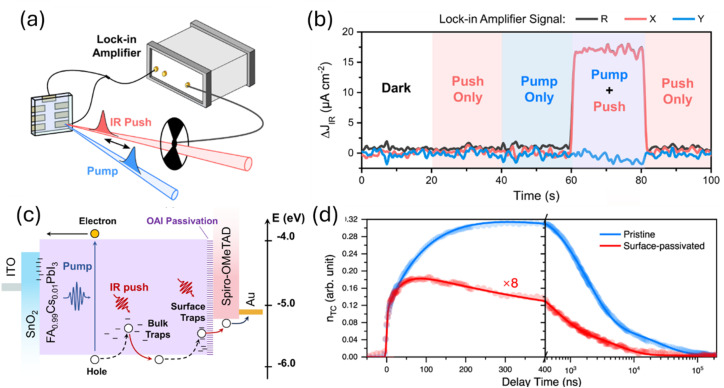
(a) Schematic of the pump-push photocurrent setup that involves a perovskite sample with contacts, a pump pulse creating electrons and holes, an IR push pulse to detrap trapped electrons or holes and an electrical detection system (lock-in amplifier) to measure the current flow. (b) Measurement of the additional current due to the IR push pulse as a function of excitation conditions showing that only if the pump pulse and the push pulse are on simultaneously, an excess current Δ*J*_IR_ is flowing. (c) Schematic of the effect of pump and push pulse on charge carrier generation and charge detrapping. (d) Trapped carrier density as a function of time for samples with (“surface passivated”) and without (“pristine”) OAI passivation layer between absorber and Spiro-OMeTAD hole transport layer. Reproduced from ref. 93 under the terms of the CC-BY 4.0 license. © The authors of ref. 93 (2023).

One conceivable approach to implement such a trap-selective measurement is the so-called pump-push photocurrent method. This approach has been used previously in the context of organic photovoltaics to study exciton dissociation and charge separation at donor–acceptor heterointerfaces.^92^ Recently, it has been applied to the quantification of the dynamics of traps in halide perovskite samples,^93^ whereby the authors used two different variants, namely a quasi-steady state version and a time-resolved version. Both experiments are performed using a setup as schematically shown in Fig. 8a. The pump laser creates free electrons and holes in the perovskite absorber layer of a solar cell. Some of these free electrons and holes might be trapped in the bulk or at the surface of the perovskite film. The IR push pulse can now selectively excite trapped charge carriers in such a way that they contribute to the measured photocurrent. Fig. 8b shows that the additional IR photocurrent detected by the lock-in amplifier is only present if pump and push lasers are both on, showing that it needs both the creation of free carriers followed by trapping and the detrapping *via* the IR push to see a signal. Fig. 8c schematically shows the processes that lead to photocurrent generation. The measurement can now be done using steady-state lasers with the only modulation being the chopper needed for lock-in amplification (quasi-steady-state) or using pulsed lasers, whereby the result is plotted as a function of time between the arrival of the pump and the push pulse. In each case, the two lasers allow two degrees of freedom, namely, to modify the intensity of the pump and of the push laser. Pan *et al.*^93^ show that in the quasi-steady state case, the variation of push laser intensity combined with the detection of the photocurrent *J*_pump_ due to the pump and the additional photocurrent Δ*J*_IR_ due to the IR push allows one to quantify the absorption coefficient of the traps, which should be linearly proportional to the density *n*_t_ of trapped charge carriers. Thus, we can write13
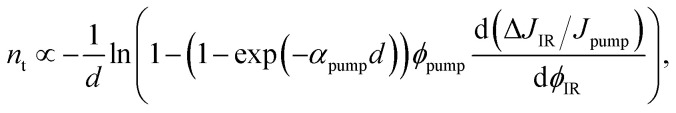
where *α*_pump_ is the absorption coefficient of the perovskite at the pump laser wavelength, *d* is the thickness, and *ϕ*_pump_ and *ϕ*_IR_ are the pump and IR photon flux of the respective lasers (in photons per area and time). In case of the transient experiment, the equation is given as14
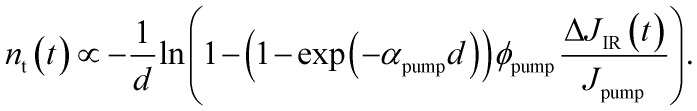


Fig. 8d shows an example of the application of eqn (14) to two different samples, whereby one (“pristine”) featured no passivation between the absorber and the hole transport layer (Spiro-OMeTAD) and the other sample (“surface passivated”) had an *n*-octylammonium iodide (OAI) passivation layer between absorber and HTL. We note the relative magnitude of the signals is considerably different as the data from the surface passivated sample is multiplied by 8 and therefore in absolute numbers much lower than that of the unpassivated sample. Thus, we can conclude that a significant effect of the trapping will come from the surface. A second observation is the time dependence of the initial rise of the signal, which may be due to different energetic or spatial distributions of trap states. For instance, it may take more time to fill surface states than bulk states as the filling of surface states would initially require diffusion of free electrons or holes to the surface.

#### 3.3.3 Opportunity

The pump-push photocurrent method is so far a rarely used method in the context of halide perovskites and far from being in any way a canonical method in the sense as discussed in Chapter 1.1. Thus, it is premature to discuss the full impact of the method on quantifying traps. However, the method has clear advantages relative to many other methods that are frequently employed in publications on halide perovskite solar cells as it does not suffer from the limitations of charge-based methods, is not limited by the detection threshold discussed in the context of Fig. 4 and does not require excessively complicated equipment. The main ingredients for the setup are two lasers and a lock-in amplifier. Furthermore, the method is applied to finished solar cells which is appealing insofar as it does not require the fabrication of special samples such as *e.g.* in the case of Hall-effect measurements which will be discussed in Chapter 6.1. An apparent downside is the proportionality sign in eqn (13) and (14), which originates from the unknown value of the optical capture cross section of trap states. However, in many practical situations, relative information (*e.g.* sample A has a ten times higher trap density than sample B) is already highly useful, which suggests that the method might find more widespread application in the future.

## 4. Charge-carrier lifetimes and recombination coefficients

**Fig. 9:**
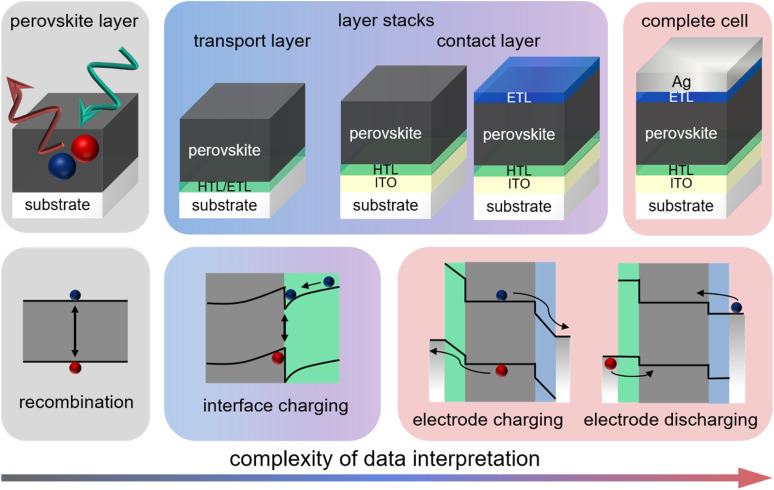
Upper row: layer stacks from films on a glass substrate *via* films with transport and contact layers, such as ITO, towards complete cells. Bottom row: schematic band diagrams showing the processes that affect transient photoluminescence measurements. Figure reproduced from ref. 33 under the terms of the CC-BY 4.0 license. © The authors of ref. 33 (2021).

Recombination is a crucial loss process for solar cells because it reduces the flux of collected charge carriers, which leads to photocurrent but also reduces the energy per extracted electron, that is, the photovoltage. A typical method of quantifying recombination is to use a charge-carrier lifetime, as discussed in Section 2.4, or a recombination coefficient. Recombination coefficients are typically used for higher-order recombination mechanisms, such as radiative recombination, Auger recombination, or sometimes Shockley–Read–Hall recombination *via* shallow traps. Furthermore, recombination can be studied in semiconductor films, layer stacks, and complete devices (see Fig. 9). The key issue here is that interface recombination is often a rather important factor in understanding recombination in a device.^60,94–96^ Therefore, studying recombination on a film alone could be considered only moderately useful for understanding the final device. However, the more complex the sample structure is, the more complex the interpretation of the measurement methods will become.^33^ This is schematically shown in Fig. 9 which shows a range of different sample structures that could be analysed using optical methods that do not require contacts. The complexity of data analysis increases from left to right. In this chapter, we use the same approach, start with the least complex situation (films), and work our way up to the most complex (complete solar cells).

### 4.1. Films

#### 4.1.1 Introduction

The recombination coefficients and lifetimes of halide perovskite films are typically determined by their steady-state or transient photoluminescence (PL). As an alternative to transient PL, a range of other transient measurements are regularly used that involve transient absorption, transient reflectance, or transient photoconductivity measurements. All these methods have in common that they analyze an observable that is related to the charge-carrier concentration as a function of time. The observable can be proportional to the product of the electron and hole concentrations (*e.g.* in tr-PL) or a weighted sum (in all other methods). The weighting of the sum can be related to the mobility (*e.g.* in transient photoconductivity Δ*σ* = *q*(*μ*_n_Δ*n* + *μ*_p_Δ*p*)) or the density of states (*e.g.* in transient absorption).

The key metric used for steady-state PL is the luminescence quantum efficiency *Q*^lum^_e_, while the key metric for transient PL is the lifetime *τ* of the decay. There is no general or simple relationship between the two quantities. The luminescence quantum efficiency is given as the ratio of the radiative to total recombination rates while considering the efficiency *p*_e_ = 1 − *p*_r_ of light outcoupling, where *p*_r_ is the probability of reabsorption. When neglecting Auger recombination, we then obtain15
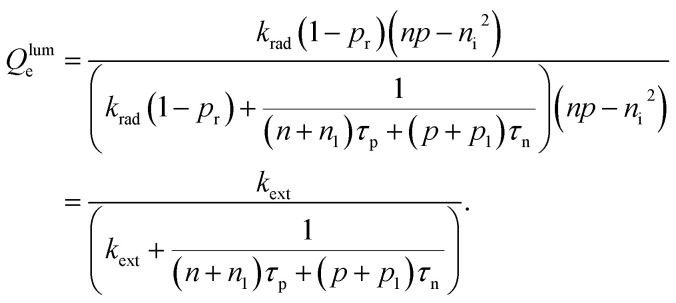
where we used the abbreviations *k*_ext_ = *k*_rad_(1 − *p*_r_), where *k*_rad_ is the radiative recombination coefficient, as well as *n*_1_ = *N*_C_ exp(−(*E*_C_ − *E*_T_)/*kT*), *p*_1_ = *N*_V_ exp((*E*_V_ − *E*_T_)/*kT*), *τ*_n_ = (*β*_n_*N*_T_)^−1^, *τ*_p_ = (*β*_p_*N*_T_)^−1^. Furthermore, *n*_0_ and *p*_0_ are the equilibrium electron and hole concentrations, *β*_n_ and *β*_p_ are the capture coefficients of electrons and holes, *E*_C_ is the conduction band edge, *E*_V_ is the valence band edge, *E*_T_ is the trap energy, *N*_C_ and *N*_V_ the effective density of states in the conduction and valence band, and *N*_T_ is the trap density. In the simple case, where the defect is deep, and the semiconductor is intrinsic, eqn (15) simplifies to16
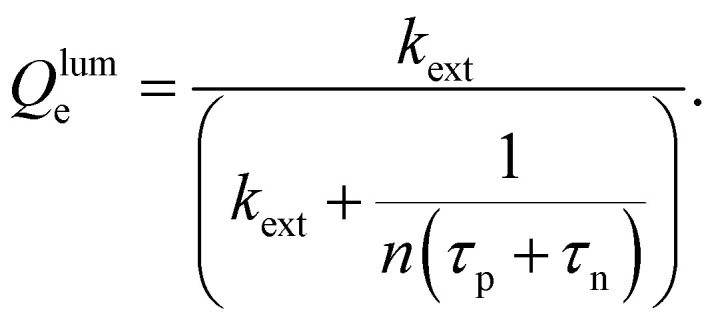


In the logic of eqn (16) (deep defect and *n* = *p*), we can also determine an effective lifetime *τ*_eff_ that encompasses both recombination mechanisms (radiative and SRH) as17
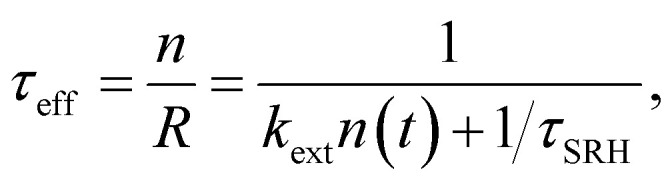
where *R* is the total recombination rate.

An alternative quantification of recombination is possible using an effective recombination coefficient that includes both radiative and nonradiative processes. In the logic of eqn (16), the effective recombination coefficient is simply the denominator of the luminescence quantum efficiency, that is,18
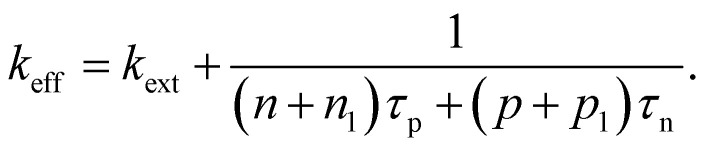


It is clear from the equations above that it is not, in general, possible to determine *τ*_eff_ from the luminescence quantum efficiency, except if the relation between *n* and *p* is known. For *n* = *p* for instance, we would obtain

19 *τ*_eff_ = (*k*_ext_*n*((*Q*^lum^_e_)^−1^ − 1))^−1^.

Thus, if *k*_ext_ and *n* can be determined separately, the lifetime can be directly determined from the luminescence quantum efficiency under these simplifying assumptions. Thus, the lifetime can only be deduced from steady-state experiments making strong assumptions.

In the case of transient photoluminescence, we observe the opposite situation. Now, the decay time can be deduced from the experimental data quite directly, while the comparison with, for example, a steady-state luminescence quantum efficiency, is much less straightforward. Again, we face the problem of making assumptions regarding the relation between *n* and *p*. If we assume the sample to be intrinsic as well as the absence of asymmetric trapping of one type of charge carrier (photodoping), the differential equation governing the decay would be given by20
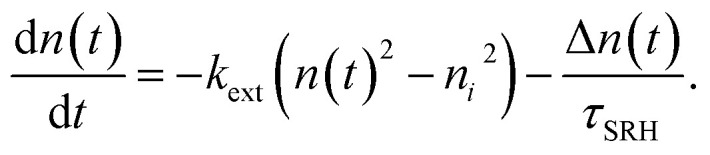


A sensible definition of a decay time would be given by^36^21
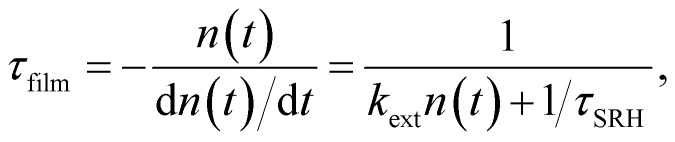


Note that there is no need to work out the solution for *n*(*t*) from eqn (20) to determine the decay time *τ*_film_. The implicit solution of eqn (20), which gives the decay time, can be determined without knowing *n*(*t*). Eqn (21) is notably identical to eqn (17); that is, in the case of a deep defect and *n* = *p*, there is no difference in the information obtained from the steady-state or transient data.

**Fig. 10:**
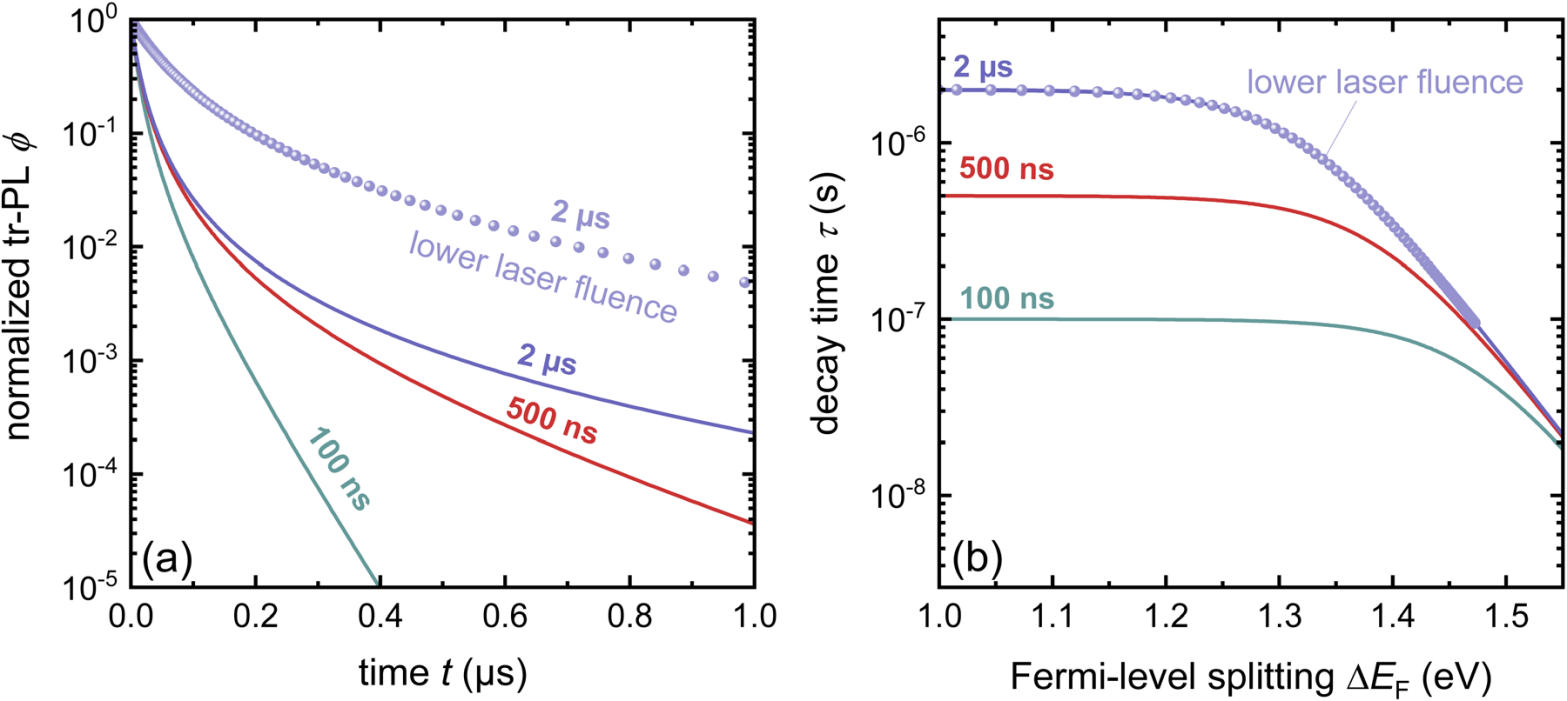
(a) Calculated transient photoluminescence decays at the same fluence (solid lines), assuming different SRH lifetimes (100 ns, 500 ns, and 2 μs). The spheres represent the same material parameters as the solid blue line but with a lower laser fluence. (b) Decay time *vs.* Fermi level splitting derived from the data in (a). Notably, the blue solid line and the spheres overlap, indicating that the plot of decay time *versus* Fermi-level splitting is unaffected by the assumed laser fluence in this simple example. Therefore, the effects of laser fluence are usually a signature of effects such as diffusion or trapping.

Any steady-state or transient data on one of the three figures of merit should consider the carrier density or Fermi-level splitting. This is because all the equations discussed thus far show different regimes, that is, a constant decay time (or effective lifetime) for low carrier densities and a 1/*n* dependence for high carrier densities. Fig. 10 illustrates the effects of nonlinear recombination on the PL decay shape. The solid lines represent the calculated PL decays with different SRH lifetimes and the same fluence, that is, the same initial carrier density, *n*(*t* = 0). The dashed line has the same material parameters (*k*_ext_, *τ*_eff_) as the 2 μs SRH lifetime curve (blue). However, the fluence is lower. Hence, the decay appears different, and it is not immediately obvious that the two curves (blue and black dashed lines) originate from a sample with the same properties. However, if we plot the decay time according to eqn (21), the blue and black dashed curves lie perfectly above each other. The difference between the two is within this range. The black dashed curve starts at lower Fermi level splitting. Thus, plotting the decay time *vs.* Fermi level splitting allows us to create a representation of the data that is independent of fluence.

#### 4.1.2 Challenge

**Fig. 11:**
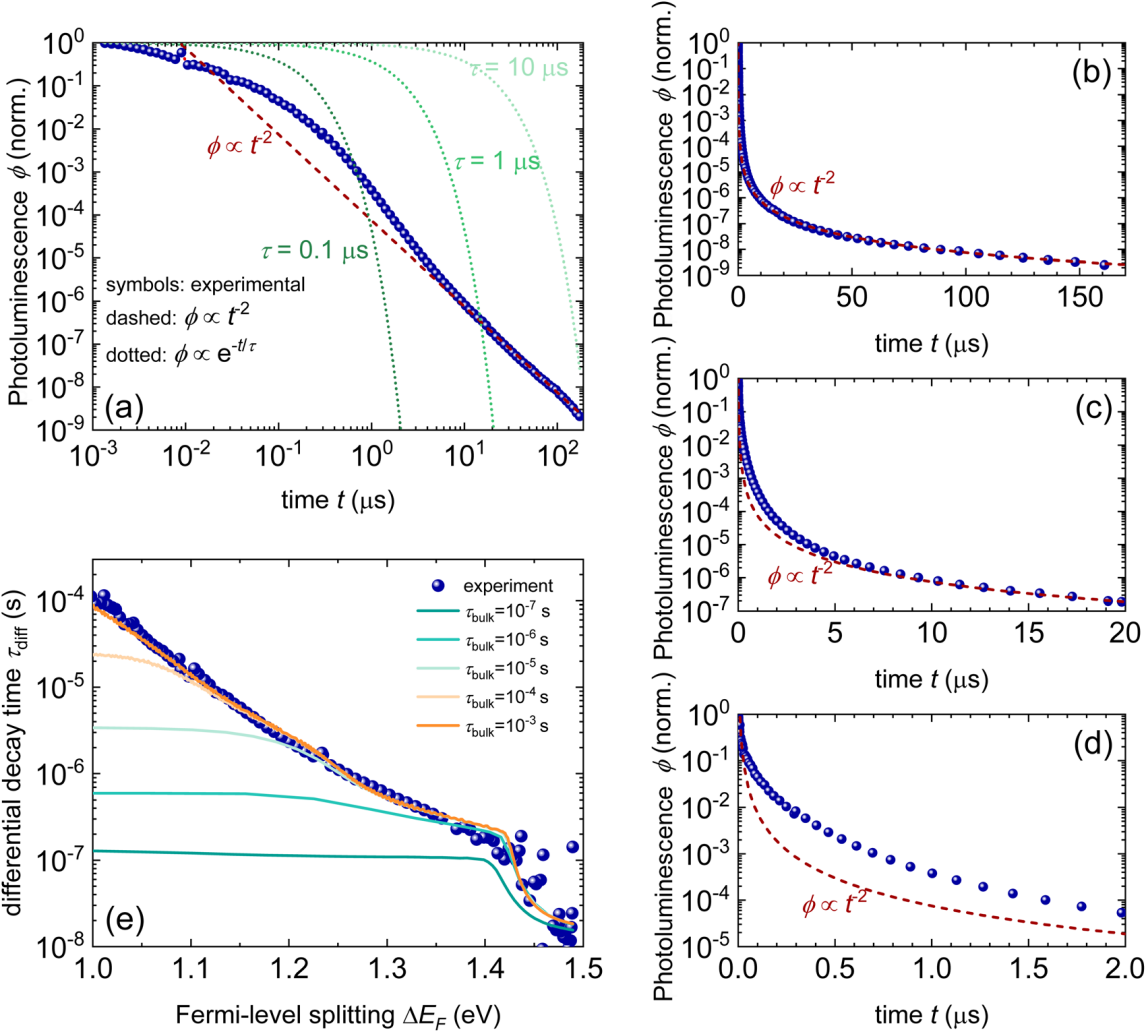
(a) Double-logarithmic representation of the tr-PL decay of a triple-cation perovskite film (blue symbols) shows that the decay follows a power law from approximately 5 μs onwards. The green dotted lines indicate the shape of the exponential decays, and the red dashed line indicates the shape of the power law with a slope of −2. (b)–(d) Same data shown with a linear time axis. In these depictions, it is not clear whether the decay is a power law or multi-exponential decay. (e) Decay time *vs.* Fermi-level splitting data derived from the tr-PL decay shown in (a)–(d) (blue symbols). The lines show simulations assuming shallow defects plus one deep defect with a lifetime, as indicated in the legend. The parameters of the fit can be found in the ESI of ref. 22.

Although both transient and steady-state methods are frequently used, they are rarely analysed in combination. The few attempts to quantitatively model both datasets show that fitting both data simultaneously is highly challenging.^22,23^ Furthermore, data are often analysed using the working hypothesis of first-order recombination. Thus, transient data are often fitted using exponential or bi-exponential fits, even if the data do not follow exponential decay.^25^ The difficulty here is that non-exponential decays are difficult to distinguish from exponential decays, as shown in Fig. 11a–d. On a double-logarithmic representation of the tr-PL decay of a triple-cation perovskite film (blue symbols), the power-law nature of the decay is clearly visible. However, on a semilogarithmic plot with a linear time axis as shown in Fig. 11b–d, the decay looks like it could very well be a double-exponential decay. Furthermore, even on the double-logarithmic plot, the power law only becomes visible from several microseconds onwards. Thus, many representations of tr-PL decays in the literature might be power laws but it may be impossible to judge from the range of data presented.

The second obvious question triggered by power-law decays is what the meaning of the decay time is in the presence of higher-order recombination. The decay time continuously increases with time and thereby with decreasing Fermi-level splitting. In the example shown in Fig. 11, the decay time varies from tens of ns to >100 μs. As the PL quantum yield is approximately 2%, the origin of the power-law decay cannot be simply radiative recombination.^22^ It must be a non-radiative recombination mechanism that is quadratic in charge carrier density. A possible explanation is shallow defects that have the same recombination dynamics as radiative recombination without being radiative in nature.^22^

#### 4.1.3 Opportunity

**Fig. 12:**
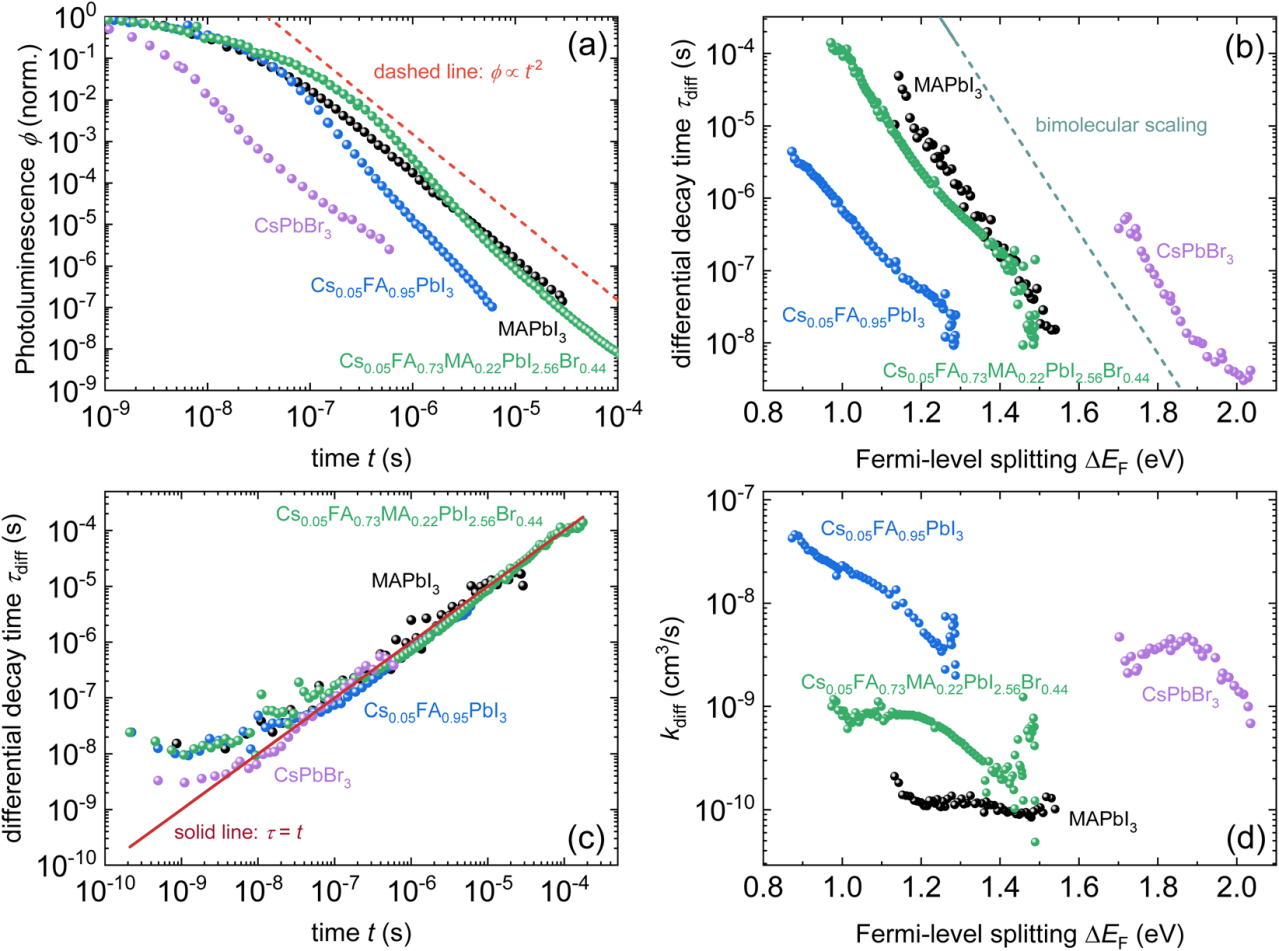
(a) Photoluminescence transients of different lead-halide perovskite films on glass with the stoichiometry as indicated in the figure. For reference, a power law of type *ϕ* ∝ *t*^−2^ is shown. (b) Differential decay time *τ*_diff_*versus* Fermi-level splitting Δ*E*_F_ and (c) *versus* time *t*. (d) Bimolecular recombination coefficient *k*_diff_*versus* Δ*E*_F_ for the same samples. Figure reproduced from ref. 25 under the terms of the CC-BY 4.0 license. © The authors of ref. 25 (2025).

We propose to measure data over a high dynamic range (see Fig. 11) and analyse both transient and steady-state data in combination.^22^ A high dynamic range can be obtained by measuring with different fluences^30^ or by changing the gain settings in situations where a gated CCD with a variable gain is employed. This allows for easier detection of power-law decays. As in our experience, power-law decays are nearly always seen in lead-halide perovskites (see Fig. 12); therefore, we propose to consider alternatives to the determination of lifetimes. One option could be the determination of nonradiative recombination coefficients as discussed for instance in ref. 25 and illustrated in Fig. 12d.

In addition to the conceptional aspects of analysing film data properly, there are also experimental considerations that become important especially in the context of the next chapters on comparison between film data and layer stacks. Here, the implicit or explicit assumption of many papers is that the addition of transport layers typically reduces the luminescence and the decay time relative to the surfaces to the glass substrate on one side and the N_2_ atmosphere of a sample holder on the other side. However, this assumption may not necessarily be true and thus, strategies have been developed to create better reference samples by passivating the two interfaces. Notable molecular passivation layers are 1,6-hexylenediphosphonic acid (HDPA)^97^ at the perovskite/glass interface and (3-aminopropyl)trimethoxysilane (APTMS)^59,98^ or *n*-trioctylphosphine oxide (TOPO).^94,99^

### 4.2. Layer stacks

#### 4.2.1 Introduction

One of the major reasons for the early successes in the field of lead-halide perovskite photovoltaics was that the contact layers typically used in the field of solid-state dye cells, namely TiO_2_ and Spiro-OMeTAD are by some coincidence nearly perfect contact layers for the compositions researched at the beginning of the development of the field (mainly MAPI). Since then, many different compositions have been developed^100^ and suitable transport layers had to be developed that provide a good compromise between passivation and efficient charge extraction.

#### 4.2.2 Challenge

In the field of molecular photovoltaics, it has been common to study exciton diffusion and dissociation *via* transient or steady PL,^101^ whereby the reduction (quenching) of the PL was considered an indication of successful exciton dissociation.^102^ In the language of perovskite photovoltaics, a rapid initial decay of the transient PL was therefore often associated with fast charge extraction. However, at the same time, fast charge recombination could also lead to a rapid decay of the transient PL which makes it difficult to interpret the data. In most methods, a qualitative comparison between samples is simple, as there is often a clear correlation between sample quality and the observable (*e.g.* more steady-state PL is always better than less). However, in the case of the transient PL, a clear correlation was no longer observed. Faster decays could be better or worse depending on the context, which makes the interpretation of the method more susceptible to confirmation bias. If one wants to explain better fill factors, then a fast decay might be interpreted *via* a change in charge extraction, and if one wanted to explain differences in open-circuit voltage, one may interpret it in terms of recombination. This risk was easy to identify,^103^ but the key challenge was how to use and interpret the method without risking a bias in interpretation.

**Fig. 13:**
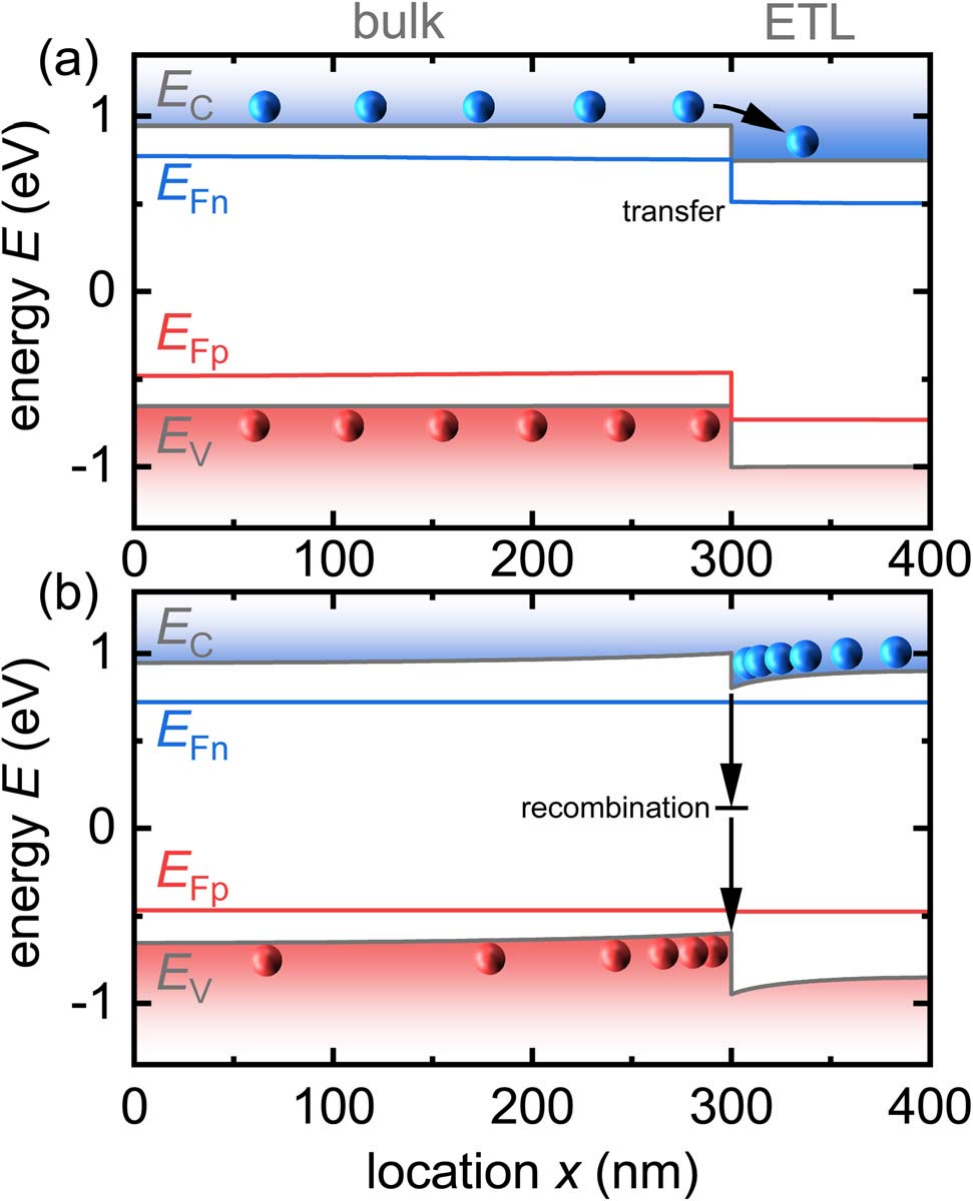
Schematic of (a) charge transfer after photogeneration of charge carriers in the perovskite bulk, followed by (b) charge accumulation and recombination. Note that if recombination is sufficiently slow, electrons in the transport layer will have a sufficient density to accumulate close to the interface and attract holes to the other side of the interface, thereby accelerating recombination relative to the situation where space-charge effects are negligible. Modified after ref. 34. © The Royal Society of Chemistry (2018).

Unfortunately, the problem seems to be difficult to treat analytically.^33,34^ The reason for this is shown in Fig. 13. After photoexcitation of the electron–hole pairs in the absorber layer of our layer stack, a current flows to equilibrate the quasi-Fermi levels. As initially, the layers that are not absorbing the light well (*e.g.* contact or transport layers) do not contain excess carriers, there will be charge transfer from the absorber to those layers. Fig. 13 shows a relevant case for halide perovskites, where both the perovskite and the electron transport layer (ETL) are intrinsic. In this situation, charge transfer to the ETL leads to the accumulation of electrons inside the ETL and the build-up of space charge. This space charge counteracts further charge transfer but also accelerates recombination by attracting holes in the perovskite to the interface. The space-charge effect seen in Fig. 13b depends on the laser fluence (how many charge carriers are there to begin with?) and the interfacial recombination rate (how long do they live?).

**Fig. 14:**
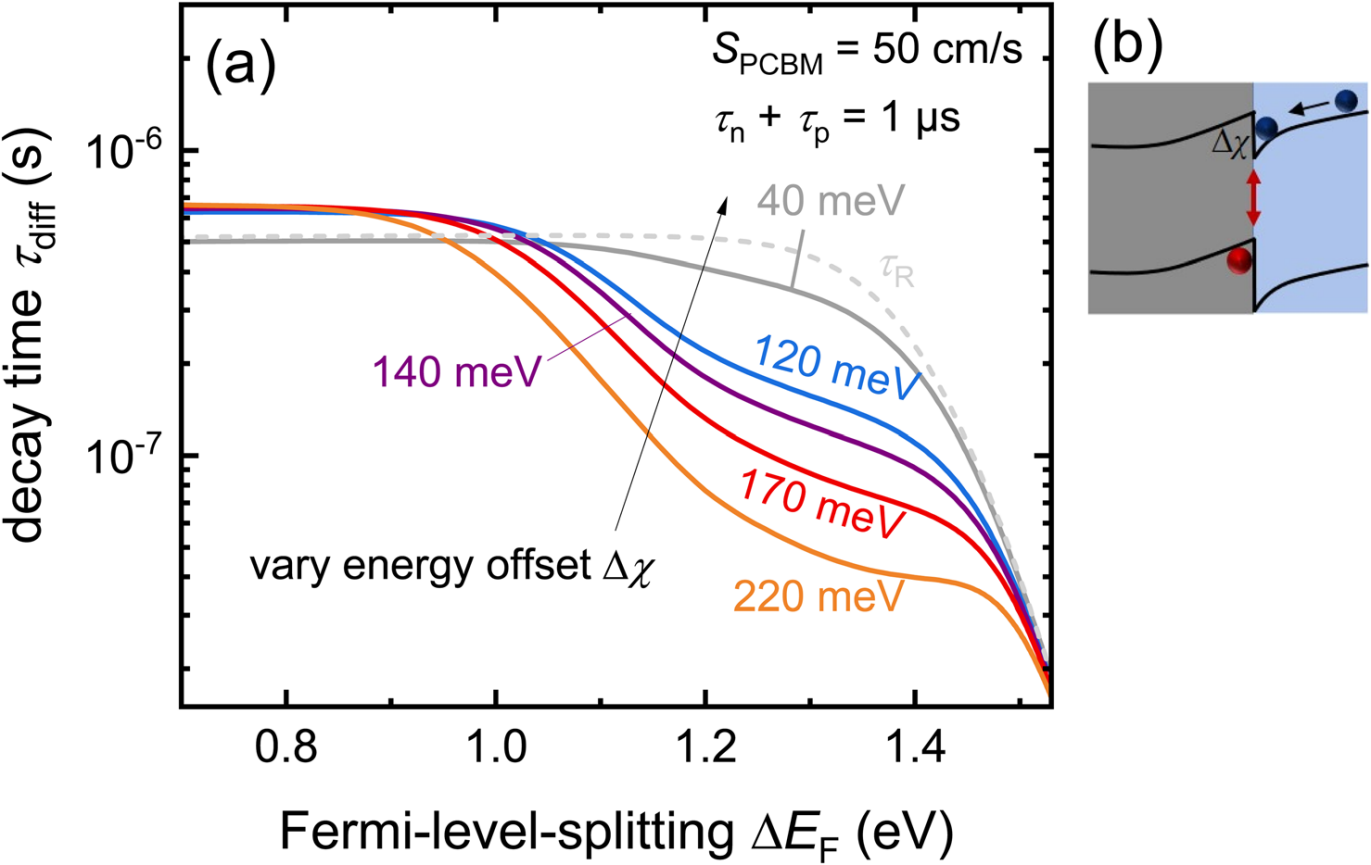
(a) Decay time derived from simulated tr-PL measurements of a perovskite-ETL bilayer as a function of band offset. The higher the offset at the interface (see (b)), the more pronounced the dip in the decay time at intermediate Fermi-level splittings (1.2 eV to 1.4 eV). Towards low Fermi-level splittings, the Coulomb attraction between electrons in the ETL and holes in the perovskites (see (b)) has become negligible and the effect of the offset disappears. Figure redrawn from ref. 33 under the terms of the CC-BY 4.0 license. © The authors of ref. 33 (2021).

The interfacial recombination rate depends on the recombination velocity, *S*, and the band offset. Fig. 14 illustrates how the lifetime depends on the Fermi level splitting for the case of a bilayer, as shown in Fig. 13 as a function of the band offset Δ*χ*, defined as the difference in electron affinity between the absorber and the ETL. We note that the higher the band offset, the lower is the decay time in an intermediate range of injection conditions. At long times and low Fermi-level splittings, the effect of the offset disappears. Unfortunately, the physics contained in Fig. 13 and 14 could not be simulated using rate equation models that (only) consider charge transfer and recombination across the heterointerface. Instead, the Poisson equation (eqn (1)) must be solved to consider the Coulomb attraction between electrons and holes across the interface. The importance of the Poisson equation implies that a full drift-diffusion simulation is already the simplest conceivable type of simulation that would consider the relevant physics.^34^

#### 4.2.3 Opportunity

**Fig. 15:**
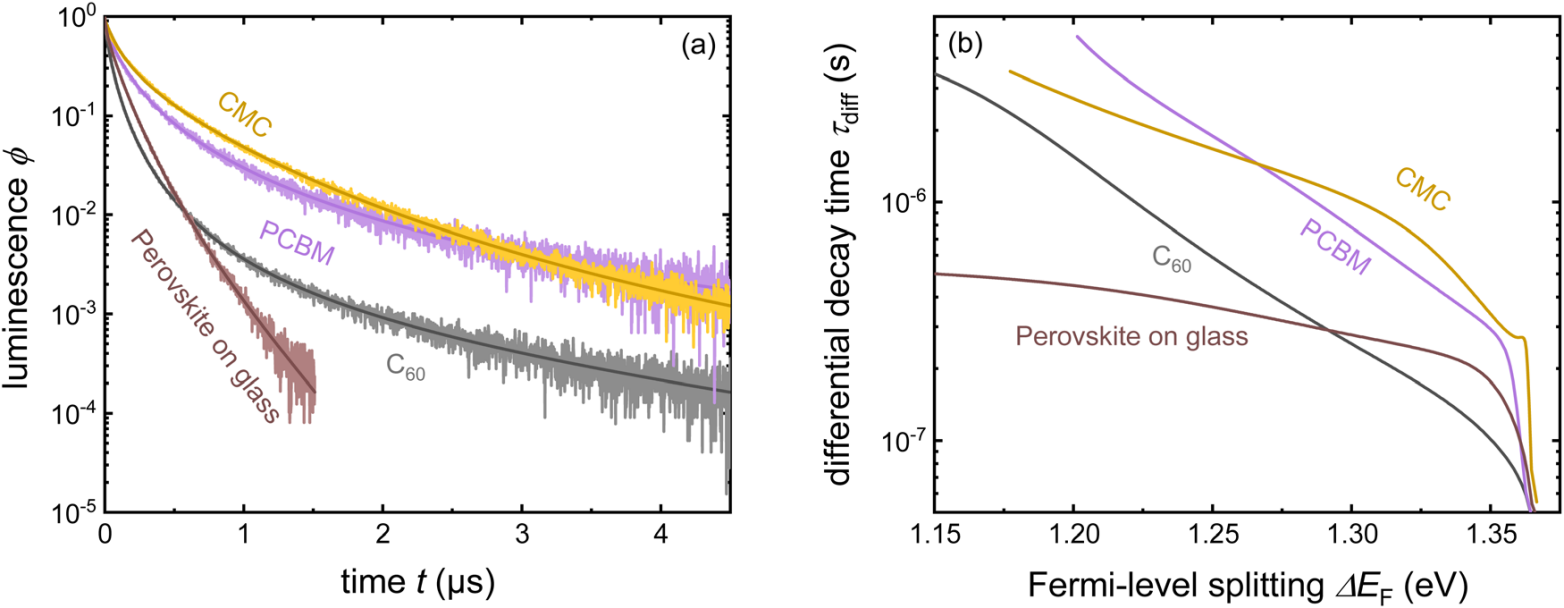
(a) Tr-PL measurements of three different samples with the layer stack Me-4PACz/triple-cation perovskite/ETL, where the ETL is PCBM, CMC, or C_60_. Furthermore, the tr-PL decay of the perovskite film on glass is presented as a reference. (b) Differential decay times were calculated from the fitted tr-PL data as a function of Fermi-level splitting. Figure reproduced from ref. 30 under the terms of the CC-BY 4.0 license. © The authors of ref. 30 (2023).

Despite its apparent simplicity, the bilayer configuration contains a range of relevant complications that make the analysis of PL transients challenging. Fig. 15 shows some example data taken on half cells that all share the same hole-transport layer (glass/ITO/Me-4PACz), the same perovskite and different electron-transport layers (C_60_, PCBM, and CMC).^30^ Furthermore, a perovskite film on glass is added for comparison. It is noteworthy that the decays correlate with the eventual device performance, however, not necessarily in an obvious way. Among the three ETLs, the fastest decay is seen for C_60_, the ETL with the highest electron affinity among the three. This is also eventually the ETL with the best fill factor, *J*_sc_ and lowest recombination losses at short circuit (see Fig. 19b). Thus, the initial fast decay could be related to the need to transfer more electrons to the C_60_ before Fermi levels equilibrate as in the case of the lower electron affinity fullerenes (PCBM and CMC) and this higher electron affinity is likely to support faster charge extraction.^104^ Thus, it is likely that the decays contain valuable information, but the extraction of this information is not straightforward.

Thus far, there are three types of models found in the literature. There are models that only consider one layer (the perovskite) and consider the ETL or HTL attached in one effective boundary condition.^60,105–107^ Here, the major disadvantage is that these models do not contain any band offset, so they would not be able to capture any of the physics contained in Fig. 14. Rate equation models^108,109^ could consider the ETL or HTL explicitly but would fail to account for Coulomb interactions. Finally, drift-diffusion simulations^34,61,110^ would account for all effects, but they are more time-consuming than the other two approaches and require learning a software tool (for instance from among the list found in Table 2).

Thus, future work will likely have to use time-dependent drift-diffusion simulations for data analysis, which is unfortunately the most complicated approach of the ones mentioned above. The challenge will then be to infer parameters from the decays. The decay depends on the energy level alignment as well as on the interface recombination dynamics and it is not entirely obvious how to disentangle the effects of both.^61^ In situations like that, Bayesian inference approaches such as the ones presented in ref. 40, 111 and 112 are likely to become highly useful. Bayesian inference means to fit simulations to experimental data and while doing so record the likelihood of material parameters (recombination velocity, band offset, *etc*…) being consistent with the experimental data. Bayesian inference thereby allows not only to identify the most likely material parameters explaining a certain experiment but also allows quantifying the confidence in the result and the need to add additional experimental data to improve the confidence.^41^

### 4.3. Devices

#### 4.3.1 Transient photoluminescence and photovoltage

##### 4.3.1.1 Introduction

Eventually, the spectroscopic results that are experimentally obtained on films or layer stack only matter if they have a predictive power on the performance of devices. In addition to *e.g.* correlating spectroscopic results on films with the performance of devices (see *e.g.* Fig. 14 in ref. 113) it is therefore logical to also extend the same forms of spectroscopy used on films to the complete devices. While the existence of metal contacts is a necessary condition for spectroscopy that involves electrical detection of signals (*e.g.* impedance, transient photovoltage), it is at least not an unsurmountable obstacle for purely optical spectroscopy such as transient or steady state photoluminescence.^33^ While steady-state PL going from films to complete devices was adapted quite early on and is reasonably straightforward as a method (see *e.g.* ref. 95 and 114), applying transient methods to devices adds an additional component, namely capacitive charging and discharging currents (*J* = *C* d*V*/d*t*) related to the capacitance *C* of the electrodes and the changes in external voltage *V*.

##### 4.3.1.2 Challenge

**Fig. 16:**
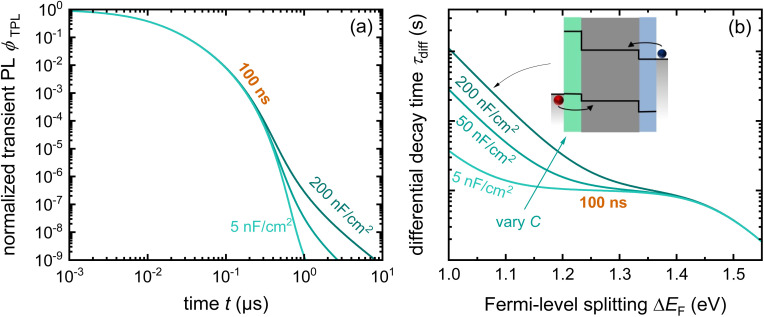
(a) Simulated transient PL decays as a function of time for three different values of the device capacitance. Differential decay times resulting from the three decays showing the effect of the capacitance, which makes the decay times become longer towards lower values of the Fermi-level splitting. Redrawn after ref. 33 under the terms of the CC-BY 4.0 license. © The authors of ref. 33 (2021).

In the case of devices, the complexity of all attempts to determine charge-carrier lifetimes is increased by the existence of the geometrical capacitance of the electrodes, which leads to various RC time constants that are not recombination lifetimes but may affect transient or frequency-domain measurements. It is often possible to distinguish between different types of time constants based on their dependence on the voltage or light intensity. Thus, the frequently observed habit in the literature on halide perovskites to show only one decay of transient photovoltage or only one arc of a Nyquist plot in impedance is unhelpful because it makes it impossible to attribute these time constants to different physical phenomena. Fig. 16 provides an example of a simulated tr-PL decay of a perovskite solar cell, where the electrode capacitance of the device was varied in the simulation.^33^ This leads to different decays at longer times that lead to a continuously rising differential decay time towards lower Fermi-level splittings. This behaviour is caused by the RC time constant of the electrode capacitance discharging *via* the recombination resistance of the solar cell, which can lead to extremely long decay times if the voltage is sufficiently low and the recombination resistance therefore sufficiently large.

##### 4.3.1.3 Opportunity

All small-signal transient and frequency-domain measurements of halide perovskites contain at least two features that can be analysed. For instance, in the case of transient photovoltage measurements, the rise and decay are visible in each transient. Traditionally, only the decay has been analysed, but the rise also contains information. Furthermore, the two time constants that can be determined at each light intensity and voltage can be compared with the voltage dependence of the different physical phenomena that can occur within a perovskite solar cell. These phenomena include different types of recombination, extraction, and discharge of the electrodes by reinjecting charge carriers through the charge-transport layers back into the absorber where they can recombine.

**Fig. 17:**
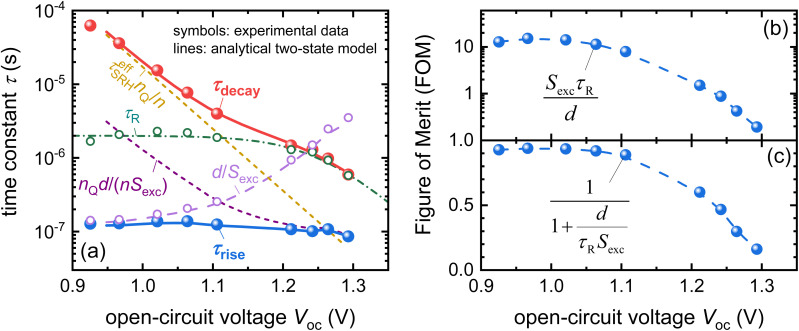
(a) Time constants extracted from the TPV rise and decay compared with the different terms in eqn (22) and (23) which are shown as dashed or dash-dotted lines. (b) and (c) Figures of merit that follow from the analysis of the rise and decay times shown in panel (a). Reproduced from ref. 17 under the terms of the CC-BY 4.0 license. © The authors of ref. 17 (2023).

A possible mathematical approach to compare experimental data with a model is to create a linearised model of two coupled differential equations describing the time dependence of the external voltage, as well as quasi-Fermi level splitting. This is a sensible approach, as the quasi-Fermi level splitting inside the perovskite absorber layer at open circuit should be reasonably constant in good devices. Such a model with two linear differential equations can be written as a matrix equation, where the time constants are given by the negative inverse eigenvalues of the matrix.^17^ The major advantage of 2 × 2 matrices is that their eigenvalues are typically simple enough to connect them with a physical meaning. In the following, we describe the results for the case of transient photovoltage measured using a bias light that keeps the solar cell at open circuit. The cell is then perturbed by illumination with a pulsed laser, which creates a small additional open-circuit voltage. The Δ*V*_oc_ first increases and then decreases again such that before the pulse and at long times after the pulse, the Δ*V*_oc_ is zero and the open-circuit voltage is entirely given by the bias light intensity. There are two well-distinguishable time constants, one for the rise and one for the decay of the Δ*V*_oc_. The eigenvalues can be determined analytically and further Taylor-expanded to simplify the equations. The rise time then follows as^17^22
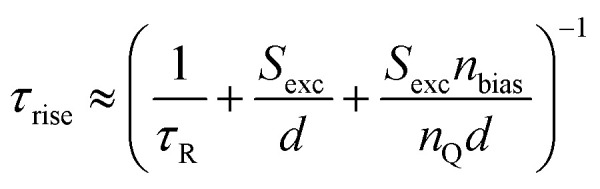
and the decay time as23
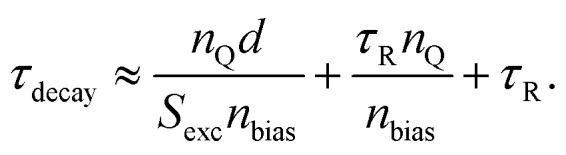
Here, *τ*_R_=(1/*τ*^eff^_SRH_ + 2*k*_rad_*n*_bias_)^−1^ is the actual recombination lifetime that contains the information on SRH and radiative recombination. The capacitance of the electrodes is contained in the parameter *n*_Q_ which is defined as *n*_Q_ = 2*C*_el_*kT*/(*q*^2^*d*), where *C*_el_ is the capacitance of the electrodes and *d* is the absorber thickness. The electron density at the bias condition is given by *n*_bias_. Finally, the exchange velocity *S*_exc_ contains information regarding the speed of electron and hole extraction through the two transport layers. Fig. 17a shows the experimental rise and decay times, as well as the different terms that contribute to the time constants as a function of the bias open-circuit voltage. The decay time is the sum of several contributions, so it approaches the value of the longest of the three terms in eqn (23) for any given value of *V*_oc_. However, the rise time is the inverse of the sum of inverse time constants. Thus, it assumes the value of the smallest of the three terms in brackets of eqn (22). Based on the interpretation shown in Fig. 17, the decay time is mostly influenced by recombination at a high *V*_oc_ and capacitive discharge at a lower *V*_oc_, whereas *S*_exc_ is not relevant in this case. The rise time depends mostly on the two terms that contain *S*_exc_.

The advantage of an analysis that considers both the time constants is that information on both recombination and extraction can be obtained from the data. The likelihood of extracting charge carriers depends critically on the dimensionless ratio24
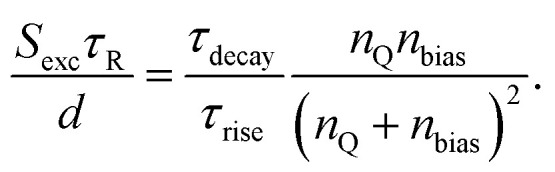


The efficiency of collecting charge carriers then follows from the ratio *via*25
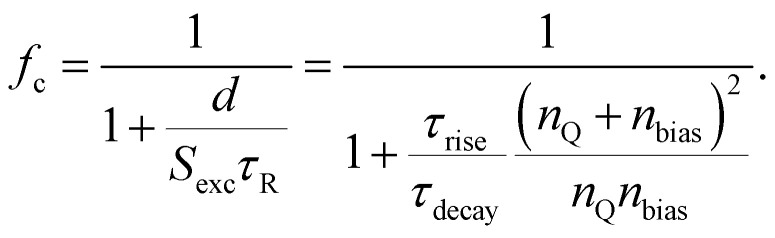


Fig. 17b and c show the two figures of merit as a function of *V*_oc_, as determined from the data shown in Fig. 17a. The main advantage of the method presented above for an example of transient photovoltage is that a variant of this approach applies to all small-signal methods applied to full devices. Similar approaches have already been published for methods such as impedance, as well as intensity-modulated photocurrent and photovoltage spectroscopy (IMPS and IMVS).^27^

#### 4.3.2 Frequency domain measurements (impedance, IMPS, IMVS)

##### 4.3.2.1 Introduction

In addition to time-domain methods, there are also small-signal frequency domain methods that can be applied to perovskite solar cells. The approach is usually to set a bias (DC) voltage and light intensity, then measure the AC response of the solar cell to some periodic excitation (*e.g.* a periodic voltage signal) and then study the behaviour of real and imaginary part of the transfer function (output/input) as a function of the different variables of the measurement which include primarily frequency, temperature and DC bias conditions. There are several conceivable types of excitation signals and therefore transfer functions. The most popular variants are impedance (*Z* = *Ṽ*(*ω*)/*J̃*(*ω*)), where the ratio of complex voltage to complex current is studied, as well as intensity modulated photocurrent (IMPS) and photovoltage (IMVS) spectroscopy.^115–117^ Here, the excitation is a modulated light intensity, and the measured signal is either the photocurrent or photovoltage induced by the modulated light intensity *φ̃*(*ω*). The transfer functions are therefore *Q* = *J̃*(*ω*)/*qφ̃*(*ω*) for IMPS and *W* = *Ṽ*(*ω*)/*qφ̃*(*ω*) for IMVS. Given these definitions, we note that the impedance should be the ratio of the transfer functions of IMVS and IMPS, *i.e. Z* = *W*/*Q*.^118^ This implies that two out of the three measurements should contain all information that could be obtained by measuring all three.

##### 4.3.2.2 Challenge

The purpose of the measurements is to determine time constants that allow the researchers to quantify recombination, extraction or other physical processes happening inside the solar cell in a similar manner as explained for the example of TPV in Chapter 4.3.1.^115,117,119,120^ As was the case for TPV, the obvious challenge of this approach is not so much to actually determine the time constants but to assign meaning to these constants in such a way that a high confidence in the result is obtained. This is hampered by the frequent inability to distinguish between different processes without having additional information. There are essentially four competing approaches to analyse the data and extract meaningful information, namely equivalent circuits,^117^ diffusion recombination models,^121^ matrix models^27,28^ and full drift-diffusion simulations.^27^ The most traditional approach is to use equivalent circuit models that enable fitting the real *vs.* imaginary part of the transfer function as a function of frequency. The simplest conceivable equivalent circuit model would be a resistor *R* and capacitor *C* connected in parallel leading to an impedance26
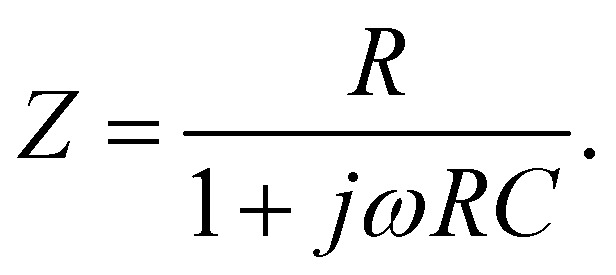


**Fig. 18:**
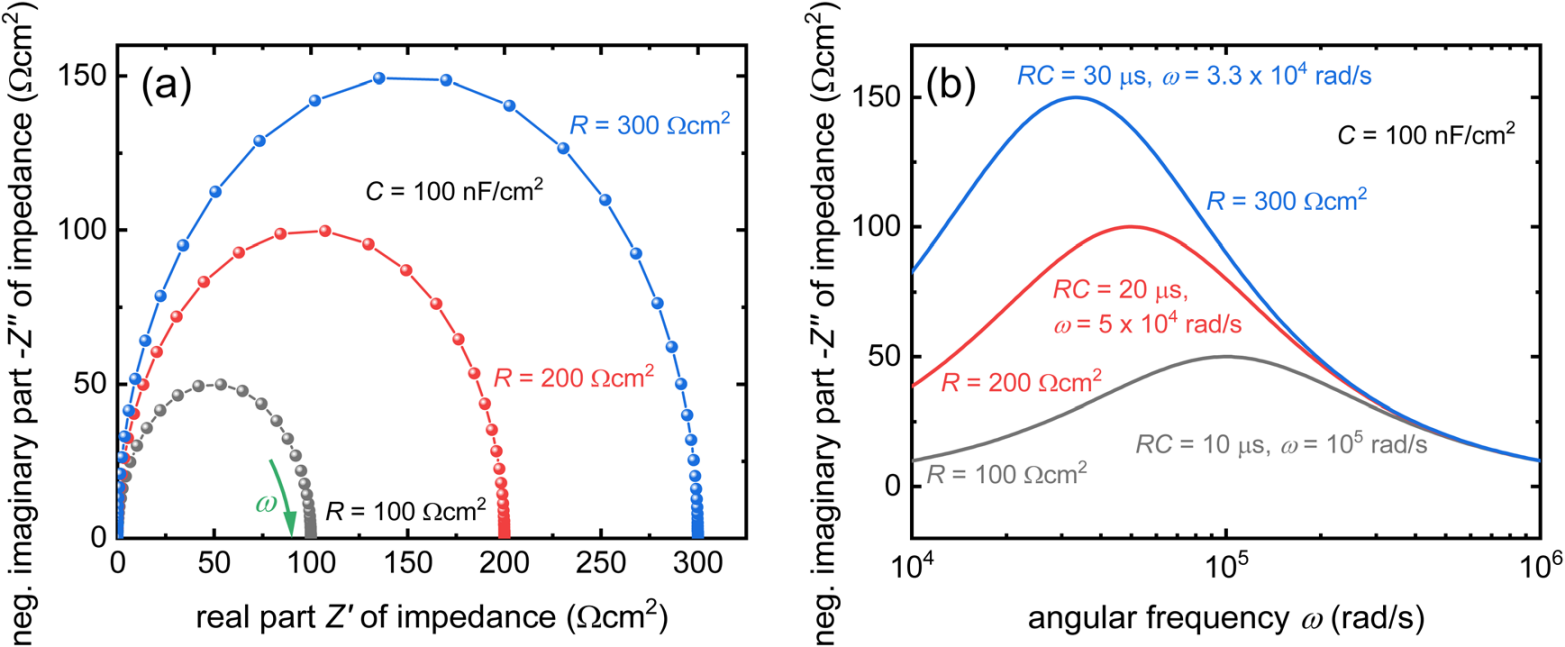
(a) Nyquist plot of an ideal parallel connection between a resistance *R* and a capacitance *C* for a constant capacitance of 100 nF cm^−2^ and three resistances. The higher the resistance, the larger the semicircle. The peak of the semicircle is reached at the maximum of the negative imaginary part (−*Z*′′) of the impedance. The higher *x*-axis intersect of the Nyquist plot corresponds in this simple case exactly to the value of *R*. (b) Plot of −*Z*′′ as a function of the angular frequency *ω* showing that the peak of −*Z*′′ always corresponds to 1/*RC*.

This impedance *Z* leads to a semicircular Nyquist plot (see Fig. 18a), where real and the (negative) imaginary part of the impedance *Z* are plotted *vs.* each other as a function of angular frequency *ω*. The maximum of the imaginary part is reached exactly if *ω* = (*RC*)^−1^ and the *RC* product is then typically extracted as a time constant as shown in Fig. 18b.

To interpret the time constant, we need to consider the different contributions to the capacitance and resistance of a solar cell as a function of bias conditions. In many situations, the *RC* time constant may indeed correlate with recombination and the open-circuit voltage which may have been partly responsible for the popularity of the method also in technology-oriented publications. Let us briefly assume the resistance of the solar cell is indeed dominated by the recombination current flowing in the dark or under illumination. The recombination current density has the typical form *J* = *J*_0_ exp(*qV*/*n*_id_*kT*), where *J*_0_ is the saturation current density and *n*_id_ is the ideality factor. Then, the differential resistance at a bias voltage *V* is27
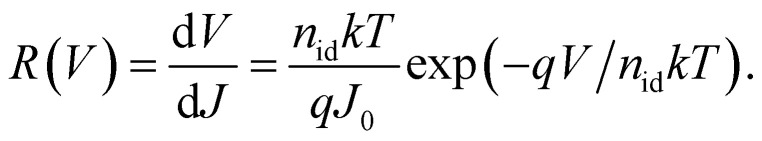


If two solar cells are primarily different in their recombination rate at a given carrier density, then they would have different values of the saturation current density *J*_0_ which contains all information on recombination except for the influence of voltage on carrier density. Thus, any variation of *e.g.* passivation quality would impact *J*_0_, thereby *V*_oc_ = (*n*_id_*kT*/*q*)ln(*J*_sc_/*J*_0_ + 1) and in consequence also the *RC* time constant if measured at a constant voltage. Note that measuring the samples at their respective open-circuit voltages would lead to a completely different trend. In this case, we would obtain28
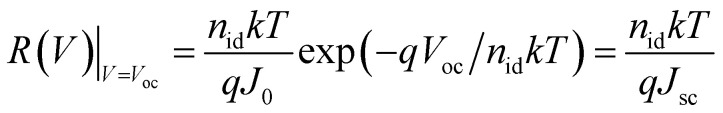
*i.e.* the recombination resistances would only reflect variations in the ideality factor *n*_id_ or (less likely) in *J*_sc_. Thus, whenever authors wish to compare Nyquist plots of different samples with each other, it is of crucial importance to unambiguously state the measurement conditions (*i.e.* at open circuit of each sample individually or at a constant voltage close to open circuit of either of the two samples).

As outlined in ref. 27, 88 and 119, even if *R* is dominated by recombination, the *RC* time constant may not necessarily be the same thing as a recombination lifetime. In many situations, the value of *C* is influenced or dominated by the capacitance of electrodes or transport layers rather than being the chemical capacitance of the absorber layer. In these situations, the *RC* time constant may still correlate with *V*_oc_ (because of *R*) but being often much longer than the actual lifetime measured by *e.g.* photoluminescence measurements.^36^ This situation is completely analogous to the case previously discussed for transient photovoltage. In Chapter 4.3.1 we discussed that the decay time of a TPV measurement is approximately29
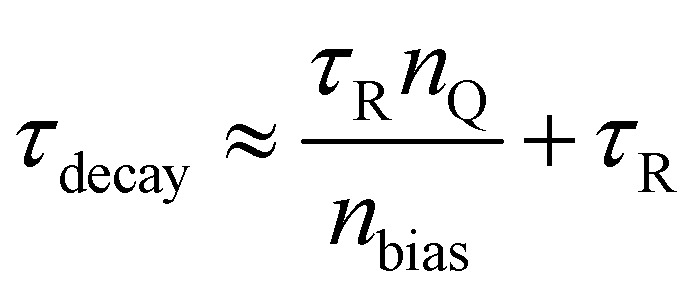
in many relevant situations (we omitted the first term for simplicity). In the logic of the typical RC equivalent circuits this can be expressed as^119^

30 *τ* ≈ *R*_rec_*C*_g_ + *R*_rec_*C*_μ_.

When the voltage and light intensity were chosen such that the second term dominates (*τ* ≈ *R*_rec_*C*_μ_), we would measure a recombination lifetime. If, however, the first term was to dominate, we would not. Thus, strategies are needed to identify the dominant term.

##### 4.3.2.3 Opportunity

In essence, the strategy to make sense of frequency domain measurements is identical to the strategy for small signal transient measurements such as TPV.^27,28,119,120^ In isolation, *i.e.* measured at one bias condition, the time constants are largely meaningless as they do not allow the researcher to identify their origin. While being meaningless in absolute terms, they might still correlate with *e.g. V*_oc_ as they might contain the recombination resistance. This may again lead to a confirmation bias of the type that the result correlates with my expectation so my interpretation of the data must be correct and likely meaningful. This can be completely wrong and further be a rather useless bit of additional evidence. Imagine the situation that you already know a trend in parameter *A*. If you now measure a parameter *B* that correlates with parameter *A* (say *via* a linear function), then knowing *B* does not help me except for confirming their correlation. Thus, the whole value of measuring a single Nyquist plot of a solar cell might provide negligible additional information if I want to understand a given trend in *e.g. V*_oc_. To infer meaning from frequency-domain measurements on perovskite solar cells, it is therefore necessary to measure over a range of bias conditions (voltages or light intensities) and then to apply a model that contains the parameters of interest. The level of complexity of the model depends on the situation. In many situations, an analytical approach *via* the eigenvalues of matrices as proposed in ref. 27 and 28 may be sufficiently complex to assign meaning to the data. Complementary measurements that are sensitive to recombination but for instance not to other terms in the equation such as the geometric capacitance are then useful to increase the confidence in the interpretation of the data. Such additional measurements could then be for instance tr-PL measurements on films or layer stacks that do not have a second electrode.

## 5. Charge transfer and extraction

### 5.1. Voltage dependent photoluminescence

#### 5.1.1 Introduction

Halide perovskite films can have extremely low surface recombination velocities when interfaced with suitable charge-transfer or passivation layers.^13,33,59,122,123^ However, a significant part of the best working transport layers from the point of view of interface passivation has fairly low conductivity.^124–127^ Many of these layers are undoped organic layers that have suitable energy levels to accept one type of carrier but not the other. Typical examples include polymers, such as PTAA, and fullerenes, such as C_60_ or PCBM, which are employed in many pin and sometimes nip device designs.^100^ As there is a range of active perovskite compositions, it is important to identify the charge-transport layers that, for a given perovskite-based absorber, have suitable energy levels and transport properties to allow the efficient extraction of the right type of carrier.^30,128,129^

#### 5.1.2 Challenge

Charge extraction initially requires that the charge is transferred from the absorber to the ETL or HTL, after which the charge carriers are transported to the contact. It is reasonable to assume that the charge transfer alone is not the sole limiting factor. Thus, transport through the ETL or HTL must be a part of the characterisation. As charge transport is only complete once the charge carriers travel through the transport layer to the metal- or metal-oxide-based electrodes, any measurement sensitive to the entire process would require electrical measurements performed on full devices that are structures with two electrodes that allow current to flow. The disadvantage of this approach is that it makes the isolation of charge-transport limitations quite challenging, as both the ETL and HTL always affect the results.^126,127,130^

#### 5.1.3 Opportunity

It is possible to selectively measure the conductivities or mobilities of the ETL and HTL separately by preparing suitable test samples. For instance, one can measure the mobilities of the ETL using space-charge-limited current measurements.^13^ This approach allows for discrimination between the two transport layers. The disadvantage is that it requires additional samples that are prepared under slightly different conditions than those of the solar cells. For instance, for SCLC measurements, the thickness of the layer whose mobility is desired must often be significantly greater (>50 nm) than the layer thickness in a solar cell (often ∼10 nm). Furthermore, finding suitable contact layers that do not affect the data can be challenging.^30^

**Fig. 19:**
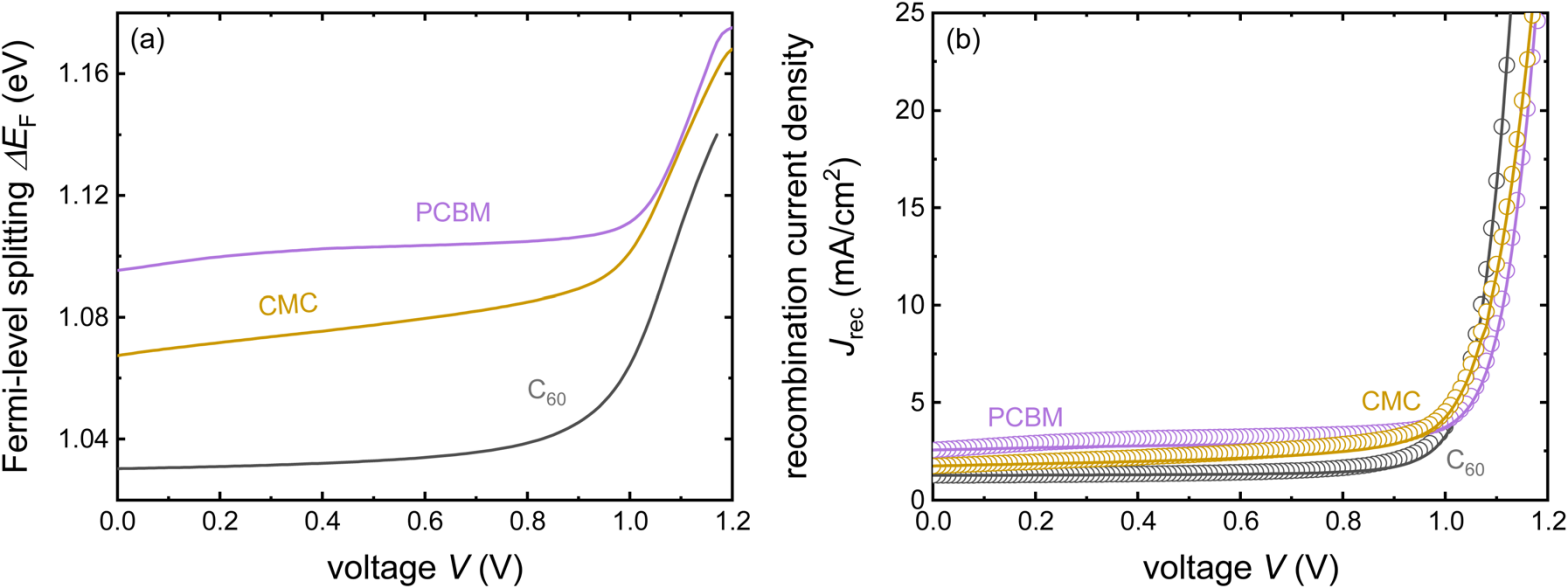
Example of the application of the voltage-dependent PL method to quantify charge collection losses. (a) Fermi-level splitting *versus* external voltage obtained from the voltage-dependent PL. Data were obtained for three solar cells that differed in the ETL used: C_60_, PCBM, or CMC. (b) The recombination current density as a function of the external voltage was calculated from the PL(*V*). Figure reproduced from ref. 30 under the terms of the CC-BY 4.0 license. © The authors of ref. 30 (2023).

If charge transfer and transport are supposed to be measured on a complete solar cell, it is often useful to study the photoluminescence of the solar cell and measure it as a function of the external voltage.^124,126,127,130^ The photoluminescence is proportional to the product of electron and hole densities in the perovskite absorber and thereby to exp(Δ*E*_F_/*kT*). This implies that the Fermi-level splitting Δ*E*_F_ can be obtained from a suitably calibrated measurement of the voltage-dependent PL. As the overall recombination current scales with Fermi level splitting, it is possible to quantify the voltage-dependent recombination current from the PL(*V*) measurements.^127^ While these data do not directly quantify the transport through the ETL and HTL, they do quantify recombination losses that are due to insufficiently fast charge collection. As the speed of charge collection is often limited by transport through CTLs, the result shown in Fig. 19 is a viable strategy for quantifying the effect of insufficiently conductive CTLs. Whenever trends as a function of the type or processing of ETL or HTL become visible (as seen in Fig. 17 for the case of a variation of ETL type), the influence of the CTLs on slowing down charge extraction and increasing recombination can be quantified. This approach is useful in halide perovskites, as the fill factors might be reasonably good, although there are still significant charge collection losses. This is due to ion-induced screening of the electric field, which leads to voltage-independent collection losses that hardly appear in the fill factor.^127^

### 5.2. Transient and steady-state surface photovoltage

#### 5.2.1 Introduction

**Fig. 20:**
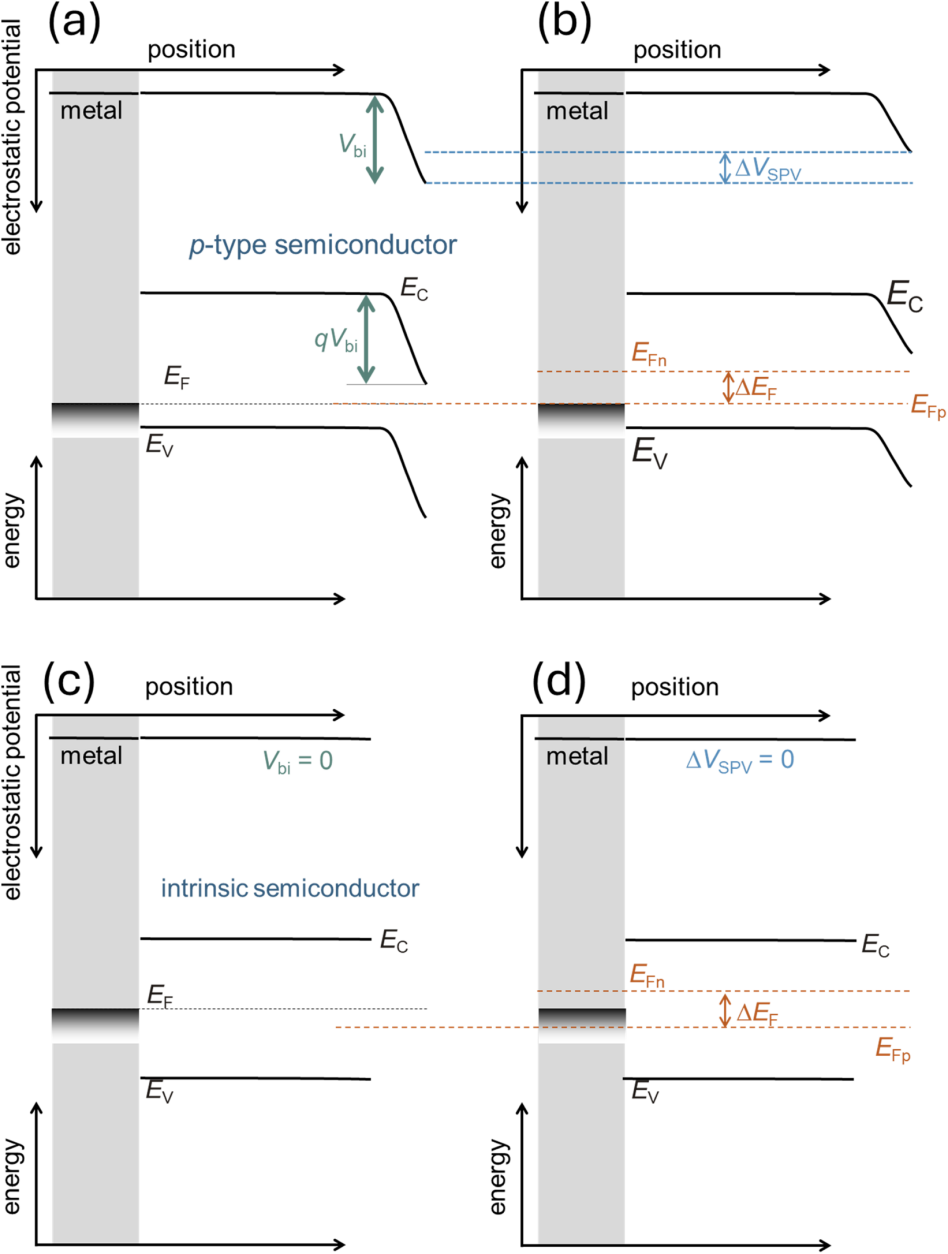
Schematic to explain the difference between surface photovoltage Δ*V*_SPV_*vs.* Fermi level splitting Δ*E*_F_ in doped (a and b) and undoped semiconductors (c and d). In the case of a p-type semiconductor forming an ohmic contact to a metal substrate, there is an electrostatic potential difference *V*_bi_ in equilibrium (a). Under non-equilibrium, low level injection conditions, the semiconductor will show a Fermi level splitting Δ*E*_F_ and an electrostatic potential difference *V*_bi_ − *V*, where *qV* = Δ*E*_F_. Thus, a PL measurement to determine Δ*E*_F_, would provide the same information as the surface photovoltage. In case of an intrinsic semiconductor with blocking contacts (c) *V*_bi_ = 0, and any illumination would still lead to zero electrostatic potential difference (d) as there is no symmetry breaking element in the system. Thus, in this case one could measure a PL but the SPV would be zero. Thus, the comparison of PL and SPV can be sensitive to the existence (or absence) of symmetry breaking elements that support charge separation.

Surface photovoltage (SPV) measurements are typically performed on half cells or layer stacks that do not form complete solar cells with two electrodes but only contain one electrode. The surface photovoltage is then measured as the voltage between that single electrode and a reference electrode that can detect the position of the Fermi level on the opposite side of the layer stack. This can be done either by an electrode that is pressed onto the sample surface and electrically insulated from the sample surface by a spacer or by a Kelvin probe. Alternatively, the surface photovoltage can also be measured using photoelectron spectroscopy methods such as UPS that are sensitive to changes in the Fermi level position relative to vacuum. The surface photovoltage is then the difference in electrostatic potential between the front and back of the layer stack as shown in Fig. 20. If the experiment is done in steady state on a complete cell, the measurement will recover the open-circuit voltage.^131^ If the experiment is however done on half cells, the surface photovoltage will be due to charge generation and separation of positive and negative charge carriers. If we briefly assume that we had an intrinsic semiconductor attached to a blocking contact (no charge injection, see Fig. 20c), the semiconductor will contain equal amounts of electrons and holes at any illumination intensity as well as in the dark. As this sample type would not contain any built-in electrostatic potential difference, there would be no charge separation and therefore also no surface photovoltage (see Fig. 20d). At the same time, the sample might show significant photoluminescence if the product of electron and hole densities are high enough. Only in situations, where any type of asymmetry exists within the sample that leads to a spatial separation of electrons and holes, a surface photovoltage can be detected. Thereby, the approach can be highly sensitive even to small changes in the net charge density due to illumination.

#### 5.2.2 Challenge

**Fig. 21:**
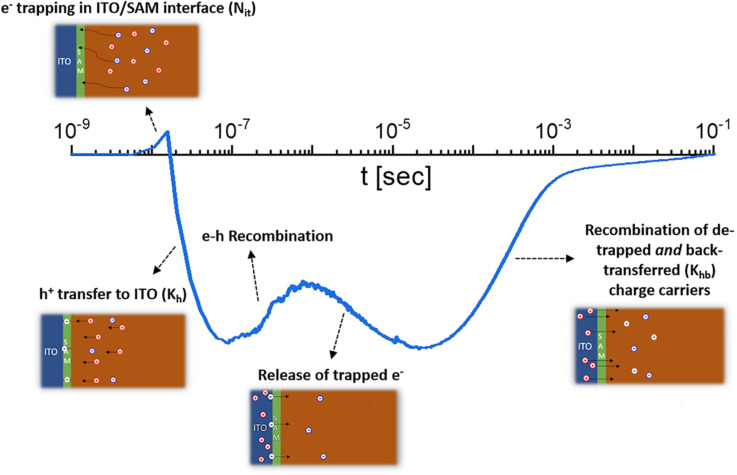
Transient SPV measurement of an ITO/SAM/perovskite sample, whereby the different features are tentatively explained using schematics of the phenomena that likely contribute to the observations. These are from short times to long times, the trapping of electrons at the ITO/SAM interface, the hole transfer to the ITO, the released of trapped electrons from the ITO/SAM interface and the recombination of detrapped charge carriers that are reinjected into the perovskite absorber. Figure reproduced from ref. 132 under the terms of the CC-BY 4.0 license. © The authors of ref. 132 (2021).

The major challenge of SPV measurements lies in the quantitative interpretation of the data as illustrated using the example from ref. 132. Here, Levine *et al.*^132^ measure transient SPV on different perovskite half cells with a glass/ITO/HTL anode but no cathode (see Fig. 21). The typical transient shows a range of features that differ in their sign, their voltage amplitude and the time at which they appear. Each feature will have at least two time constants (rise and decay), thereby leading to a multitude of time constants that are contained in the data. In this specific case, the authors of ref. 132 assign the visible features in Fig. 21 from short to long times to (i) electron trapping, (ii) hole transfer to the ITO, electron detrapping, and recombination.

#### 5.2.3 Opportunity

**Fig. 22:**
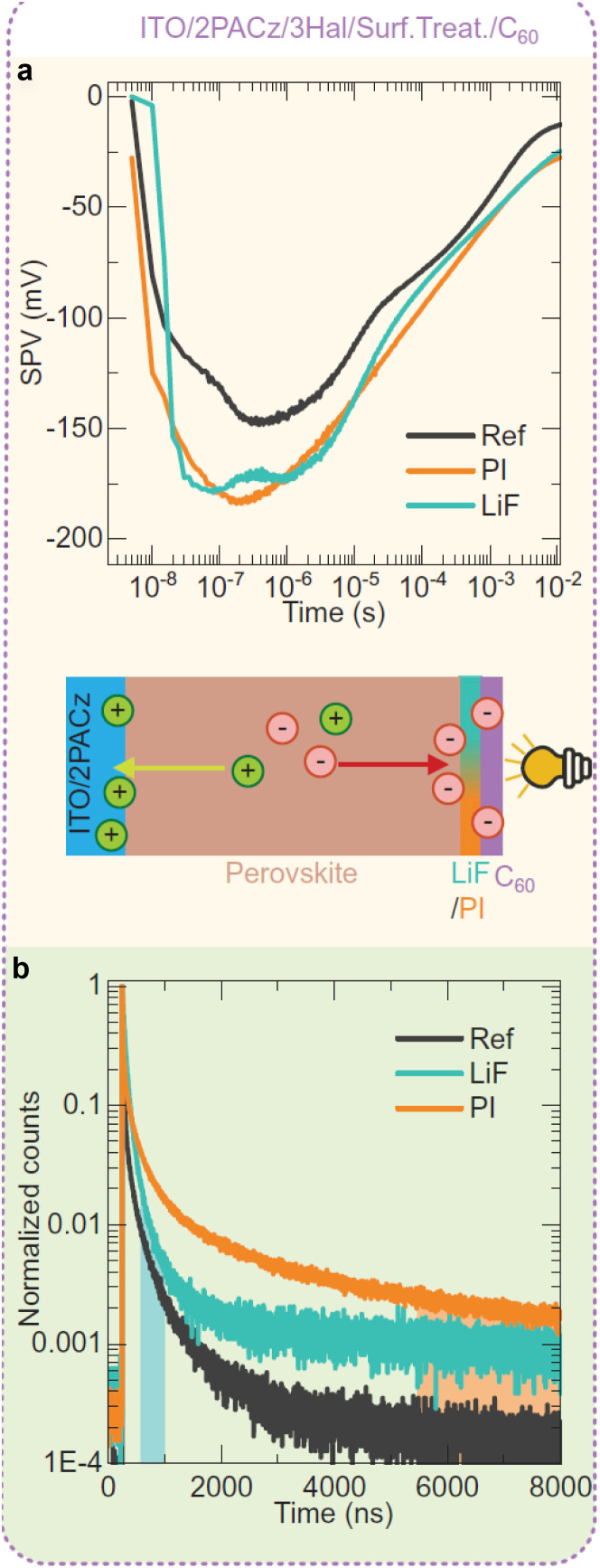
(a) Transient SPV and (b) transient photoluminescence measurement of an ITO/SAM/perovskite/C_60_ sample with different interlayers (piperazinium iodide, PI, and LiF) between perovskite and C_60_. In this study, the PI led to a sharp rise and large amplitude of the SPV signal (suggesting fast charge separation) and to slow recombination at the same time (seen in the tr-PL). This is consistent with the improved solar cell performance (see ref. 16). Figure reproduced with permission from ref. 16. © The authors of ref. 16 (2023), some rights reserved; exclusive licensee American Association for the Advancement of Science.

Unlike TPV, typical transient SPV measurements are large signal measurements that do not allow using similar approaches based on the linearization of non-linear equations as is possible for small signal measurements. Furthermore, SPV is sensitive to spatial separations of electrons and holes. Thus, any model-based analysis of the data will have to consider at least one spatial dimension thereby implying that a full transient drift-diffusion simulation is already the simplest conceivable level of theory. Thus, the data analysis of transient SPV will be more involved than that of most other techniques described in this review. However, numerical simulations are doable and specific opportunities for the method arises from its complementarity to other widely used methods such as transient photoluminescence (see Fig. 22). SPV is a rare example of a method that is specifically sensitive to the difference in electron and hole densities that creates a net charge and is thereby complementary to all methods that are sensitive either to the product (PL) or the sum (conductivity) of electron and hole densities. Thus, the application of global fitting of a combination of tr-SPV with *e.g.* tr-PL or TRMC measurements might be a powerful method to infer parameters. Ideally, such fitting would include methods to quantify confidence in the data analysis such as Bayesian inference methods. While these have been used in the context of transient PL already,^40,111,112^ they are likely being used in the future also for combinations of complementary measurements done on the same sample.

#### 5.2.4 Mobilities

The charge-carrier mobility of photovoltaic absorber materials affects the likelihood of the charge carriers being extracted at the correct electrode. In addition to high mobility, charge carriers also need to have a high lifetime, and the electrodes need to be selective for charge carriers, which can be achieved through different mechanisms. These include electrostatic effects, such as built-in voltages and asymmetries in the available densities of states achieved, for example, *via* charge-transport layers that lack electronic states at the right energy for the conduction of the unwanted carrier type, as well as kinetic effects, where the conduction of charge carriers of only one type is efficient. In halide perovskites, we observe a special scenario in which the mobilities in the absorber layer are often higher than those in the charge-transport layers. Thus, it is not *a priori* clear which mobility crucially affects the performance of the device.

**Fig. 23:**
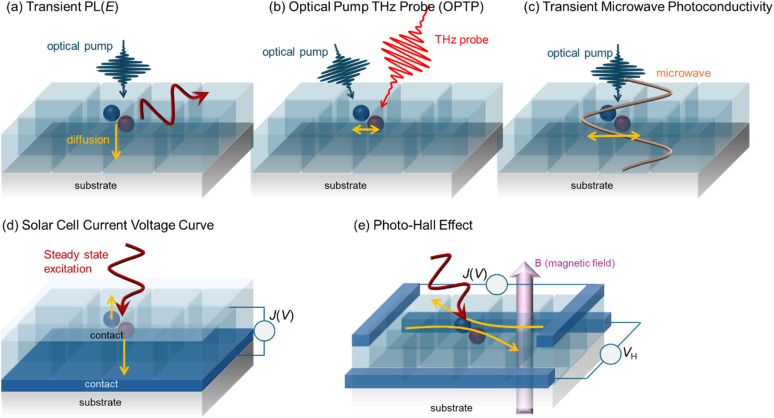
Schematic illustration of different measurement methods in the context of charge carrier mobilities applied to polycrystalline materials (inspired by Fig. 5c in ref. 135). The active layer is illustrated by transparent cubes, indicating the existence of grain boundaries that are primarily parallel to the surface normal of the film. This leads to different effects of the grain boundaries on the determined mobility or performance of the solar cell. (a) Transient and spectrally resolved PL, where the diffusion of charge carriers into the depth of the film after photoexcitation leads to a red-shift of the PL spectrum as a function of time and allows determination of the out-of-plane mobility which is relatively unaffected by the grain boundaries. (b) OPTP measurement that probes the intragrain motion of photogenerated charge carriers *via* the relative transmission change of a THz probe pulse. (c) Transient microwave photoconductivity measures the intra- or inter-grain motion of charge carriers *via* changes in the microwave reflectance. (d) Solar cell *JV* curves primarily require vertical (out-of-plane) transport within the active layer and lateral transport within the contacts (primarily the TCO) to achieve good performance. (e) The photo-Hall effect allows access to the transport properties of electrons and holes separately by measuring the Hall voltage *V*_H_ for a given applied voltage and magnetic field as a function of steady-state illumination intensity.

Meta-studies on mobilities in halide perovskites have previously found that the mobilities show a particularly strong method-specific variation^133^ as well as a strong variation between thin films and single crystals.^134^ An important reason for these variations is the sensitivity of different methods to the presence and orientation of grain boundaries. Let us assume that the grain sizes are on the order of typical thin film thicknesses (hundreds of nanometres), then grain boundaries are much more likely to affect in-plane transport as opposed to out-of-plane transport. Fig. 23 illustrates the different techniques discussed in Chapter 6 and the type of transport they are sensitive to or depending on (in the case of the *JV* curve). The spectrally resolved transient PL (Fig. 23a) measures the depth dependence of the carrier concentration profile, which homogenises as a function of time by diffusion. Homogenisation leads to larger self-absorption losses, which cause a redshift in the spectrum. Thus, this method measures out-of-plane mobilities that are particularly relevant for normal device operation (see Fig. 23d). The optical pump terahertz probe (OPTP) measures the change in THz probe transmission as a function of the time delay to an optical pump. The high frequency of THz radiation ensures that the mobility is measured on a length scale of nanometres, that is, within a single grain. Thus, OPTP often gives the highest values of mobility.^133,134^

### 5.3. Vertical electrical measurements (out of plane)

In a solar cell, transport is mostly vertical through the absorber layer and lateral only within the highly conductive contact layers consisting of metals and transparent conductive oxides. Thus, the absorber mobility of interest is generally the out-of-plane mobility.

#### 5.3.1 Challenge

It is difficult to achieve stable ohmic contacts to halide perovskites without the use of interlayers. Because these interlayers are often organic, low-mobility semiconductors, their impact on vertical electrical transport measurements is rarely negligible.^30^ Furthermore, ionic conductivities can affect the validity of many traditional methods for measuring out-of-plane mobilities. For instance, space charge limited current measurements have been widely used in organic photovoltaics.^78^ Mobile ions present in halide perovskites, however, significantly complicate the method, whereby possible workarounds involve the pulsed application of voltage^82,83^ and avoiding the use of low-conductivity charge injection layers.^30^

#### 5.3.2 Opportunity

Purely optical methods have been frequently used^136,137^ to determine mobilities or diffusion lengths within the field of halide perovskites; however, until quite recently this was primarily true for in-plane measurements of transport. Out-of-plane measurements of charge transport using purely optical techniques have been shown to be feasible in single crystals^138^ and, recently, in thin films.^32^ Photon reabsorption is the physical mechanism that allows depth resolution in purely optical techniques. Here, the photons absorbed or emitted deeper into the film undergo more reabsorption (photon recycling) than photons absorbed or emitted closer to the front of the film. This general idea of exploiting the relative ease of achieving wavelength resolution for achieving depth resolution is at a the core of quite traditional methods in photovoltaics, such as the determination of diffusion lengths from the external quantum efficiency measurement.^139,140^ However, it is also applicable to the reciprocal method of photo- or electroluminescence spectroscopy and has been used for classical photovoltaic technologies such as crystalline silicon.^141–143^ The key innovation of ref. 32 is that it showed that the method can also be applied to photoluminescence transients on perovskite thin films, is compatible with confocal microscopy, and that it thereby allows the spatially-resolved comparison of in-plane and out-of-plane measurements on the same film.

While the approach discussed in ref. 32 and 138 requires somewhat costly equipment, the general idea is feasible with relatively minor alterations to a traditional time-correlated single photon counting setup as it is available in numerous laboratories working on halide perovskites. The setup discussed in ref. 32 and 138 used either a gated CCD camera to generate spectral resolution at every delay time, or they combined time-correlated single photon counting (TCSPC) detectors with wavelength-selective filters that allow detection of a different part of the PL spectrum. The latter method was implemented in ref. 32 using a beam splitter and two detectors (each with a different filter) measuring simultaneously. In principle, it could also be performed using a single detector and by measuring the sample, for example, first with one filter and then with another. This has the huge advantage of negligible additional costs for the setup relative to those available in many laboratories. However, it has a practical disadvantage that there is no built-in mechanism to detect any changes in the sample properties between the measurements. This could be overcome by several repeated measurements to check whether any difference in the decay dynamics is due to transport or changes in the sample properties.

**Fig. 24:**
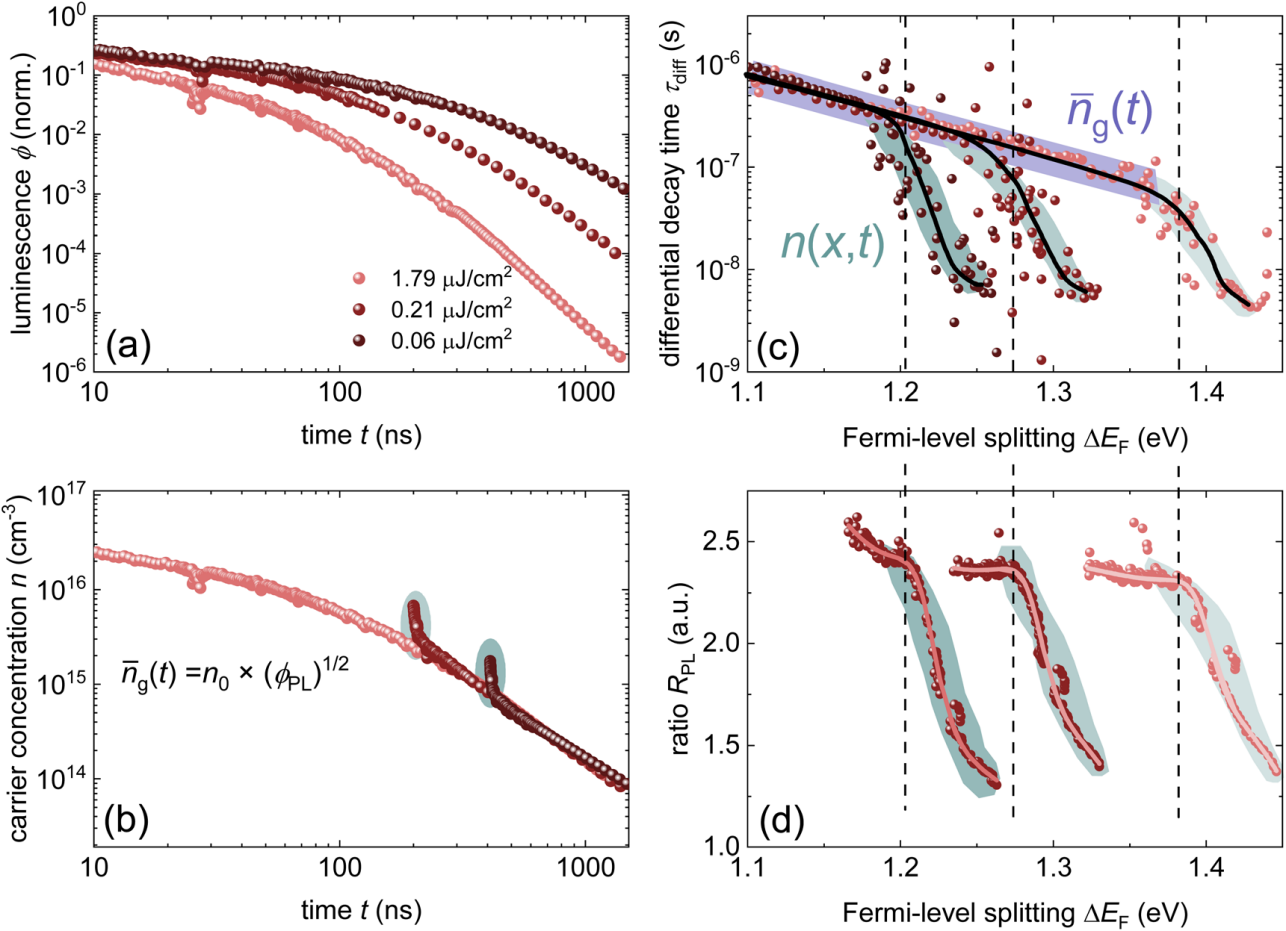
(a) Photoluminescence decays taken at different fluences on a double-logarithmic plot. (b) Same decays but now with the carrier concentration on the *y*-axis and with the time axis shifted such that the data overlays. (c) Differential decay time *vs.* Fermi-level splitting. (d) Spectral shift ratio *vs.* Fermi-level splitting. The regions highlighted in green represent regions of the decay where diffusion matters, while the regions highlighted in blue represent the part of the decay where all curves follow a common trajectory. Data for the figure and further information on the sample is found in ref. 144.

Fig. 24 shows an example of the spectrally resolved tr-PL data measured at different fluences.^144^ The samples were triple-cation perovskites, similar to those used for Fig. 10. If we assume that the PL intensity *ϕ* ∝ *np*, we can then determine the geometric mean 
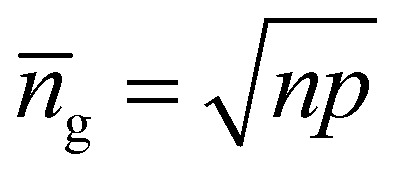
 of the charge carrier density as a function of time (see Fig. 24b). The data for the lower two fluences is offset in time such that the data fits to each other, except for a short initial part, where the carrier density decays rapidly to the its longer time trajectory. Fig. 24c shows the resulting differential decay time calculated using eqn (21). We note that the common trajectory shown in Fig. 19b leads to a common decay time where all three traces overlap. However, at the highest Fermi-level splitting for each of the three curves, the decay time was shorter than the predicted trajectory. This part of the decay time corresponds to the fast initial decay shown in Fig. 24b. Fig. 24d shows the condensed information resulting from the PL spectra, expressed as the ratio of the low-to high-energy parts of the PL spectra. At longer times and lower Fermi-level splitting, the carrier concentration becomes more homogeneous which increases the self-absorption effect and results in a redshift of the spectra. This redshift translates into an increase of the ratio *R* with time and decreasing Fermi-level splitting Δ*E*_F_. We note that the part of the data that shows a change in *R* is identical to the part of the decay time data in Fig. 24c, where each individual fluence diverges from the trajectory at longer times. Mathematically, we observe a transition from a partial to an ordinary differential equation governing the behaviour of tr-PL. At early times (highlighted in shades of green), the decay is governed by a combination of diffusion and recombination (or trapping) of charge carriers. Even in the complete absence of recombination, diffusion alone can lead to a reduction in the PL intensity as it moves carriers away from a region of high *np* to a situation where the electron and hole distributions are equally distributed throughout the depth. Even if 
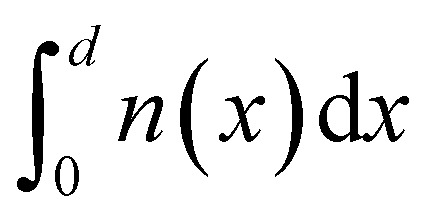
 does not change (no recombination or trapping), the term 
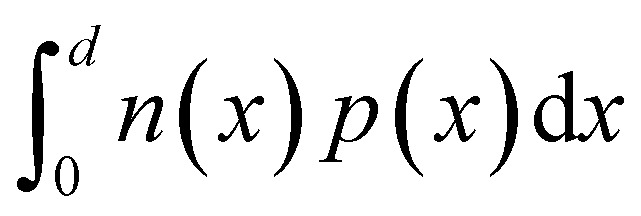
 will change just by diffusion. As long as 
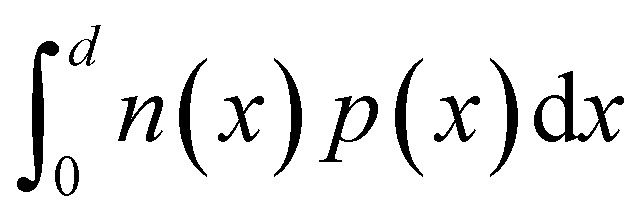
 changes differently than 
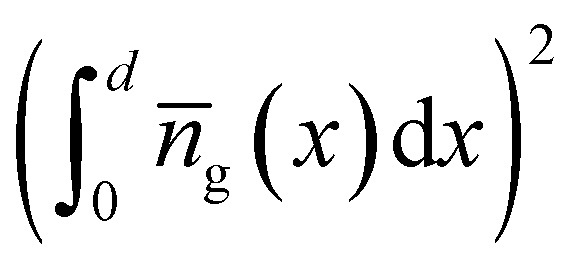
, the spatial distribution of *n*(*x*) will matter and one would have to solve a partial differential equation for the variable *n*(*x*, *t*) to simulate the decay. Once diffusion leads to a homogeneous carrier distribution (*n*(*x*) = const), the depth dimension ceases to matter, and the mathematical problem reduces to the solution of an ordinary differential equation in time. The solution of the ordinary differential equation will then depend only on *n* (and *p*) and not separately on time *t*. Thus, measurements at different fluences will end up following the same trajectory at any given carrier density *n* (or Fermi-level splitting Δ*E*_F_). From the change in the ratio *R* with time, mobility can be inferred by fitting a diffusion-recombination model to the data. This model requires precise knowledge of the optical properties of the perovskite film, which creates a challenge for data analysis. However, a major advantage of the model is that it allows the determination of both the decay time and the (relevant) out-of-plane mobility of a film using contactless measurements.

### 5.4. Lateral measurements (in-plane)

#### 5.4.1 Introduction

Lateral electrical measurements dispense with some problems of vertical measurements as they separate the width between the contacts from the thickness of the film. Therefore, the influence of the charge-transport layers is significantly reduced. In the case of optical methods, the lateral geometry allows the use of measurement techniques that would not otherwise be possible. Examples include Hall effect measurements,^145–147^ steady-state photocarrier grating technique,^148–150^ and determination of diffusion coefficients or even diffusion tensors from lateral PL decays.^136,151^

#### 5.4.2 Challenge

Lateral measurements are affected more strongly by grain boundaries than vertical measurements, as illustrated in Fig. 23. Thus, we have to assume, based on evidence such as that discussed in ref. 32 that lateral (in-plane) measurements provide a lower limit for mobilities for device-relevant vertical measurements (out-of-plane). Furthermore, in ionic–electronic conductors, such as halide perovskites, it is difficult to disentangle electronic from ionic transport using steady-state methods.

#### 5.4.3 Opportunity

The photo-Hall effect measurement^146^ is a powerful method that is one of the few that allows distinguishing between the properties of electrons and holes and that allows access to both transport and recombination. In a Hall effect measurement, a voltage is applied to a sample that leads to in-plane current flow through the sample. A magnetic field creates a Lorentz force that separates electrons and holes, leading to the creation of a Hall voltage perpendicular to the initial applied voltage and magnetic field (see Fig. 23e). The knowledge about the geometry of the sample, the measured Hall voltage, the applied voltage (and injected current) and the magnetic field allows the determination of the Hall coefficient *R*_H_, which is related to carrier densities and mobilities in limit of low magnetic fields by31
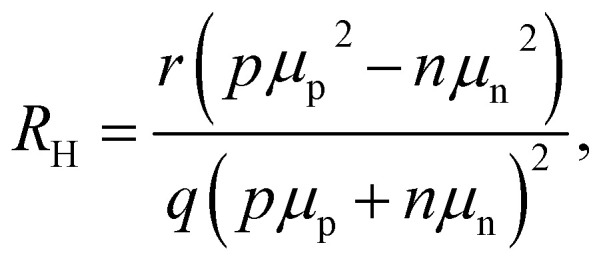
where *r* is the scattering factor (see Section 8.3.1 in ref. 152). The extension of eqn (31) towards high magnetic fields is found *e.g.* in ref. 152, eqn (8.12). Note that eqn (31) contains both the difference (numerator) and the sum (denominator) of terms related to the transport of electrons and holes. In low-level injection, where the majority carrier concentration is fixed by the doping density, the Hall coefficient simplifies to32
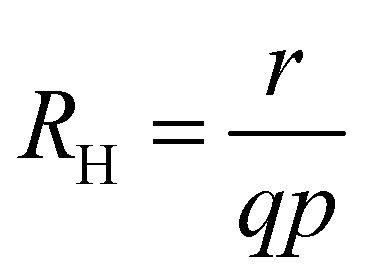
assuming *p* ≫ *n*. Thus, in a doped semiconductor in the dark, the Hall coefficient depends only on the majority carrier concentration, and thereby on the doping density. The conductivity in this case is *σ* = *qpμ*_p_, which implies that the mobility follows as *μ*_p_ = *σR*_H_/*r*. A straightforward method to modify the low-level injection condition is to illuminate the sample. If we consider the example of a (lightly) p-type sample in the dark, initially, only the hole density determines the Hall coefficient. However, under illumination, the influence of the electron density *n* and electron mobility *μ*_n_ will become increasingly important. Now, the same approach as before will allow us to determine the so-called Hall mobility given by33
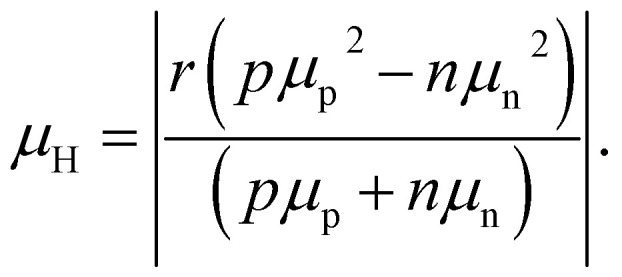


**Fig. 25:**
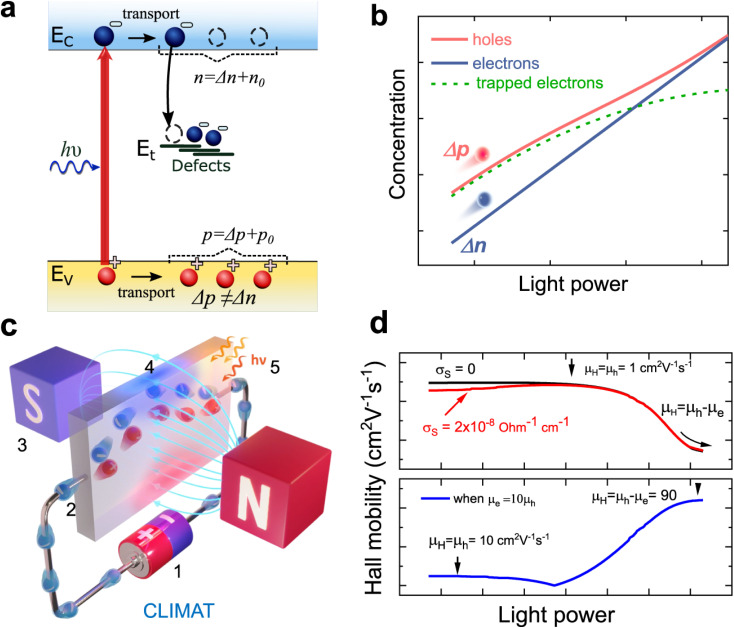
(a) Charge carrier generation, recombination, and transport in p-type semiconductor with acceptor-like defects. (b) Simulated dependence of electron, hole, and trapped electron density as a function of illumination. (c) Schematic of the Hall effect measurement under illumination, where (1) represents the current source, (2) represents the electrical contacts, (3) represents the magnet, (4) represents the induced Hall voltage, and (5) represents the illumination. (d) Simulation of Hall mobility in p-type material with identical (upper panel) electron and hole mobilities and electron mobilities that are a factor of 10 higher than the hole mobilities (bottom panel). The symbol *σ*_S_ indicates the value of the parasitic conductivity that affects the correct determination of the Hall mobility. Figure reproduced from ref. 145 under the terms of the CC-BY 4.0 license. © The authors of ref. 145 (2024).

Let us assume a semiconductor with deep acceptor-like traps (negatively charged when full and neutral when empty), such that *p* ≥ *n*, as shown in Fig. 25a. With increasing illumination, the photogenerated free electron Δ*n* and hole densities Δ*p* will start to exceed the trapped electron density and due to charge neutrality Δ*n* and Δ*p* will approach each other as seen in Fig. 25b. If we assume that *μ*_n_ = *μ*_p_, the Hall mobility approaches zero towards high light intensities, as shown in the upper panel of Fig. 25d. At low light intensities, the Hall mobility is determined by the hole mobility and scattering coefficient *r*. In this case, also parasitic conductances (called *σ*_S_ in ref. 145) can affect the result. If we assume the electron mobility to largely exceed the hole mobility, we will see the opposite trend of Hall mobility with light intensity. Now, the low illumination result will be the same, but the high illumination result will be significantly higher and will yield a difference in electron and hole mobilities.

**Fig. 26:**
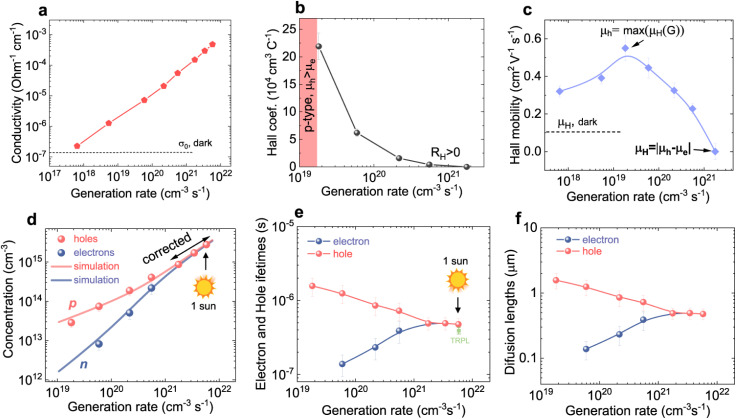
(a) Conductivity, (b) Hall coefficient, and (c) Hall mobility of a triple-cation lead-halide perovskite thin film measured as a function of light intensity and, thereby, generation rate. The decrease in the Hall coefficient *R*_H_ indicates the increasing relevance of the minority-carrier term in the numerator of eqn (31). (d) Electron and hole concentrations as a function of the generation rate. (e) Electron and hole lifetimes as functions of the generation rate. (f) Diffusion lengths of electrons and holes as a function of generation rate. Figure reproduced from ref. 145 under the terms of the CC-BY 4.0 license. © The authors of ref. 145 (2024).

Fig. 26 shows the results of the application of the photo-Hall method to a triple-cation, lead-halide perovskite thin-film sample (∼500 nm thickness), where each parameter is plotted *versus* the light intensity or photogeneration rate *G*. The sample had acceptor-like defects, such that holes were the majority carriers in the dark. The photoconductivity increases with the generation rate (see Fig. 26a) while the Hall coefficient *R*_H_ decreases (see Fig. 26b). This is a logical consequence of the conductivity *σ* = *q*(*pμ*_p_ + *nμ*_n_) depending on the sum of electron and hole conductivities, while the Hall coefficient contains a difference in hole and electron transport properties (*R*_H_ ∝ *pμ*_p_^2^ − *nμ*_n_^2^). The Hall mobility *μ*_H_ (see Fig. 26c) increases slightly (probably owing to parasitic conductance) but then drops to near zero for the highest measured illumination intensities, implying that the hole and electron mobilities are approximately equal. An important feature of the photo-Hall method is that the electron and hole densities can be determined independently from the Hall coefficient, the photoconductivity, and the knowledge of the electron and hole mobilities that can be derived from the Hall mobility. The electron density results from34
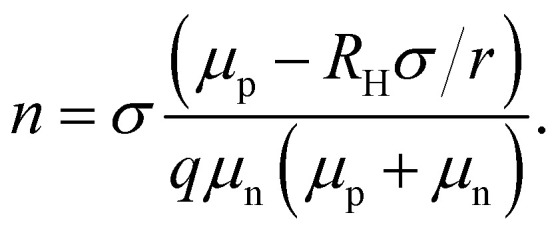
and the hole density then results from35
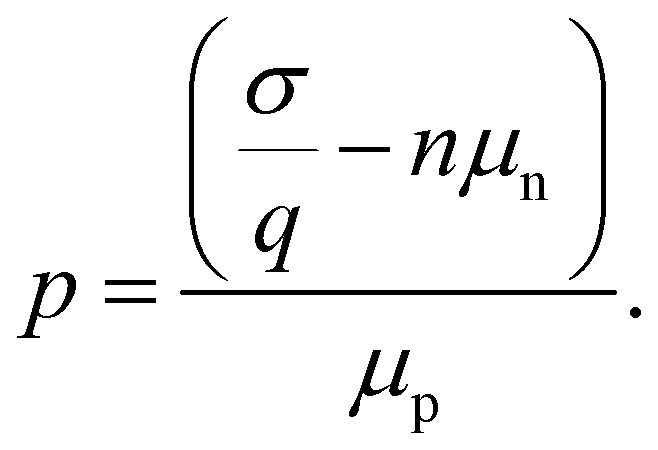


The charge carrier densities are plotted as a function of light intensity in Fig. 26d showing that they approximate each other at high light intensities, whereas the hole density exceeds the electron density at low light intensities. This suggests the existence of acceptor-like traps with a density that is sufficiently high to lead to a difference in electron and hole densities at low injection due to charge neutrality *n* + *n*_t_ = *p*, where *n*_t_ is the density of the trapped electrons. At higher light intensities, *n*_t_ cannot further increase but is limited by the total trap density *N*_t_, whereas *n* and *p* continuously increase until *n* ≈ *p*.

A further advantage of this method is that the knowledge of carrier densities allows one to derive information on charge-carrier lifetimes. The approach proposed in ref. 145 is to define an electron36
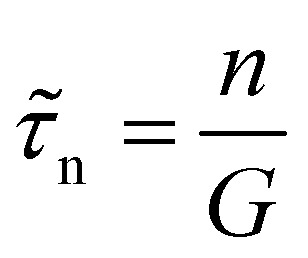
and a hole lifetime37
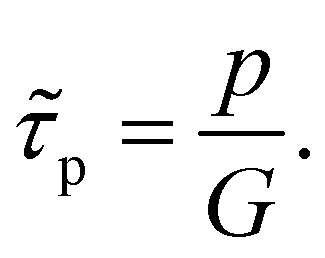


In contrast to the notation used in ref. 145, I introduced the tilde (∼) to indicate that these definitions are different from the SRH lifetimes for electrons and holes that we used previously, *e.g.* in eqn (17), (20), and (21).

It is worth briefly comparing the definitions in eqn (36) and (37) with the SRH lifetimes used previously. The data suggest a trap that is not too shallow and not too high in density, as its effect disappears towards higher light intensities. Thus, for simplicity, we assume that *G* = *R*_SRH_, and that *R*_SRH_ is limited by a deep defect. Then we can write38
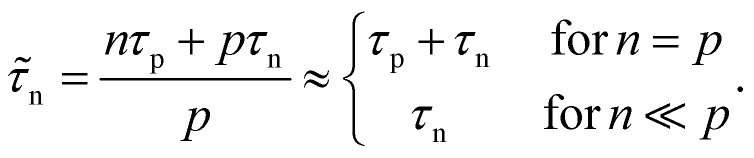


Thus, at low light intensities, where *p* ≫ *n*, we obtain the simple result *τ̃*_*n*_ = *τ*_n_ while towards higher light intensities, we obtain a higher value *τ̃*_*n*_ = *τ*_n_ + *τ*_p_. This mathematical result corresponds well to the observed effect of *τ̃*_n_ increasing with generation rate (see Fig. 26e) and then saturating to a constant value. The intuitive explanation for this trend is that for low light intensities, electrons are the minority carriers and, for every recombination event, the capture of electrons will be rate limiting, while the capture of holes will be fast in comparison. Towards higher light intensities, this is no longer true, and the capture of electrons and holes will be similarly fast, implying that both the electron and hole SRH lifetimes contribute to the measured (Hall) lifetime *τ̃*_n_. The hole lifetime instead is given by39
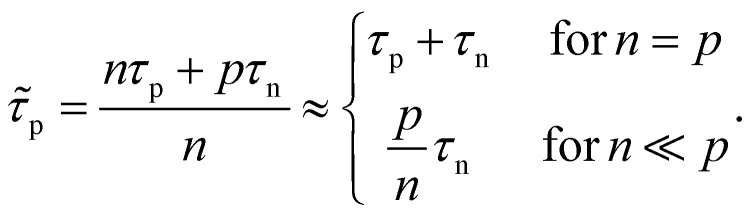
where we assume that *τ*_n_ and *τ*_p_ are similar in magnitude. Eqn (39) immediately explains why we observe the opposite trend for *τ̃*_p_ as compared to *τ̃*_n_. For low generation rates, we have 
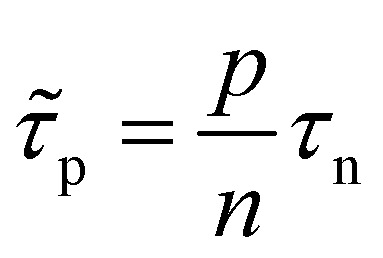
 and thus (given *p* ≫ *n*) a higher value as compared to higher generation rates, where *τ̃*_*n*_ = *τ̃*_*p*_ = *τ*_n_ + *τ*_p_. Thus, the data show a consistent behaviour for a sample with acceptor-like traps of medium density and offer strategies to connect the quantities with tilde that are directly derived from observables to the quantities without a tilde that are parameters of a model (here the SRH model) and that could be used as input parameters, for example, in numerical device simulations. Fig. 26f finally shows the electron and hole diffusion lengths based on the definitions 
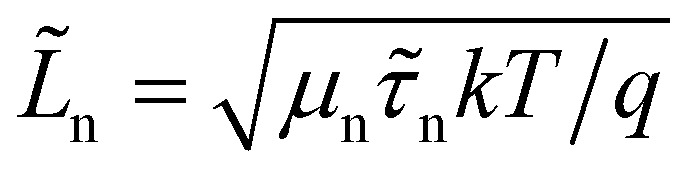
 and 
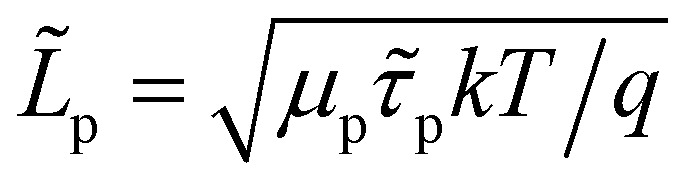
. Given the similarity in electron and hole mobilities determined using the photo-Hall measurement, these diffusion lengths show the same trends as the lifetimes discussed previously.

Thus, to summarize, the photo-Hall effect measurement provides a rather unique combination of information as it is sensitive to both transport and recombination as well as to electron and hole properties independently. The challenges in going forward will be associated with a better understanding of the effects of possible parasitic conductivities (*e.g.* ionic motion) and the translation of the parameters obtained from experiments (particularly lifetimes and diffusion lengths) to the parameters of the relevant models for recombination and transport. Furthermore, it is important to achieve a better understanding of the effects of surface passivation on effective Hall lifetimes.

### 5.5. Microscopic measurements

#### 5.5.1 Introduction

So far, I have discussed in-plane and out-of-plane mobility measurements that were both affected by grain boundaries orthogonal to the respective transport direction. Thus, to obtain a better understanding of the upper limit of the charge transport for a given material, one has two choices. Either one studies single crystals or one chooses methods that allow access to the transport properties within a single grain of a polycrystalline film. There are two non-contact methods to achieve this: optical pump terahertz probe (OPTP) and time-resolved microwave photoconductivity (TRMC) (see Fig. 23b and c). These methods and their applications to halide perovskite films have been recently summarised in a review article by Hempel *et al.*^135^ Another review article specifically on TRMC was published by Savenije *et al.*^153^ The working principles of the two methods are as follows: both techniques are pump probe techniques, where an optical pump pulse creates a certain density of photogenerated charge carriers in a thin film of a semiconductor. These charge carriers move in response to electric fields created by the probe. The probe in the case of OPTP is a terahertz pulse, and the observable is the relative change in the electric field of the terahertz pulse. From this change in the electric field, the photoconductivity can be calculated (see eqn (1) in ref. 135) as a function of time delay after the pump and as a function of the frequency of the terahertz pulse. If the initial photocarrier density is known, for example, by measuring the power of the optical pump pulse, the mobility can be derived from the initial value of the photoconductivity, whereas the time dependence provides information on trapping and recombination. In the case of the TRMC technique, the probe represents the change in the reflected microwave power within a resonator. This relative change in microwave power then again gives the photoconductance and a known initial density of charge carriers, providing mobility and information on trapping and recombination. The main differences between the methods are the different time resolutions and wavelengths or frequencies of the probe. The THz probe pulses used in OPTP are typically created using optical fs laser pulses that create THz pulses by nonlinear processes in crystals (for example, ZnTe). Therefore, the same optical fs laser pulses are often used as the pump pulse with the time delay between the pump and probe determined by the optical delay stages. This is compatible with a high time resolution; however, the physical length of the delay stages limits the time window to approximately 2 ns. However, variants of OPTP have also been developed that allow access to a longer time window of up to milliseconds by using electronic delays.^154^ The TRMC technique does not have as high a time resolution as OPTP. The time resolution is typically limited by the quality factor of the microwave resonator to times >nanoseconds. The advantage of the TRMC technique is its ability to measure very low mobilities and thereby its applicability to molecular semiconductors.^135,155^

#### 5.5.2 Challenge

The two measurement techniques discussed in this section are somewhat different from most of the methods described in this article. They have been used frequently in the context of research on halide perovskites,^108,110,122,135,153,154,156^ but they do not belong to the classical set of canonical methods used in many of the more technology-focused publications in the field. Thus, they are primarily used by groups specifically focusing on optical spectroscopy, which implies that many of the issues relevant for more widely used methods (*e.g.* PL, SCLC, capacitances) do not apply to OPTP and TRMC. Nevertheless, there are some challenges related to the interpretation of the results. Both methods measure mobility on relatively small length scales that are, however, slightly different. For the THz probe of the OPTP, the measured mobility is related to the length scales of a few tens of nanometres (see Fig. 5a and eqn (13) in ref. 135). Thus, if signs of localisation were visible in the OPTP data of perovskites, the domain sizes would have to be on the order of tens of nanometres. Usually, no signs of localisation are visible. In the case of TRMC, the length scales are approximately one order of magnitude larger (<200 nm), which implies that they are still typically smaller than the film thickness and smaller (but not much smaller) than the typical grain size.

In a round-robin study on OPTP and TRMC,^135^ the authors initially looked at published data and noted significant variations in the mobility values from these two methods. Of course, these variations in the literature could be partly due to variations between samples. These differences nearly disappeared when a series of similar samples were measured using the same setup. However, some variation was maintained when comparing measurements of the same samples using setups from different groups. Interestingly, the authors identified the cause “human error” as the biggest effect, which suggests that despite the significant variation in the obtained mobility values, there is no inherent issue with the methods that makes them less reliable or less comparable.

#### 5.5.3 Opportunity

The two methods, OPTP and TRMC, offer a range of advantages compared to other methods that make them attractive for the characterisation of halide perovskite samples. Because of their ability to probe intra-grain mobilities, especially OPTP, is attractive for estimating the upper limit of the mobility unaffected by grain boundaries, and thereby the potential benefits associated with targeting larger grain sizes or single-crystal devices (see for example, Fig. 5d in ref. 135). Furthermore, compared with other macroscopic methods, OPTP allows discrimination between trends in mobility due to stoichiometry and trends due to grain size variations. In the context of thin-film materials with shorter lifetimes, such as kesterites,^157^ OPTP have also served as a means to study trapping and recombination. In contrast, for halide perovskites, the traditional OPTP method with optical delay stages does not provide much information that can be interpreted in terms of trapping and recombination. Further applications of the recently introduced approach of using an additional laser for the optical pump pulse with electronic delay may lead to a wider application of the method in the context of studying recombination.

TRMC has already been extensively used to study recombination as a method with information content comparable to that of transient photoluminescence. An opportunity that has so far rarely been used (exception is *e.g.* ref. 111) is the combination of methods that are sensitive to *np* (such as tr-PL) and *n* + *p* (such as TRMC or transient absorption spectroscopy). This should lead to additional insights into trapping, as this would lead to imbalances between electron and hole densities (due to charge neutrality). One of the rare examples of comparing tr-PL with transient absorption spectroscopy lead to the conclusion that the effect of charged defects under illumination on recombination and recombination dynamics must be substantial in the studied multi-cation films but insignificant in MAPI.^44^

## 6. Band diagrams and band offsets

### 6.1. Band offsets and alignment

#### 6.1.1 Introduction

The ability to draw band diagrams for electronic devices is an important requirement for quantitatively predicting their functionality and performance using numerical simulations. To achieve a predictive understanding of device functionality, it is necessary to know not only the material parameters, such as the mobility and lifetime discussed so far, but also the energy levels of the conduction and valence bands. In particular, the change in energy levels at the interfaces between two materials can have a significant impact on transport and recombination across interfaces, which often make up a significant part of the losses in perovskite solar cells.^95,96^ Given the high number of thin layers and interfaces in typical thin-film solar cells, accurately determining and quantifying the properties of the relevant interfaces that limit device performance can be time consuming and challenging for a variety of reasons.

#### 6.1.2 Challenge

The typical approach to determining the positions of the conduction band, valence band, and Fermi level in semiconductors is *via* photoelectron spectroscopy, typically ultraviolet photoelectron spectroscopy (UPS). This method measures the valence-band edge and Fermi-level position relative to the vacuum level. Typically, the position of the conduction band edge is estimated by adding the optical bandgap to the position of the valence band edge. Alternatively, inverse photoemission spectroscopy (IPES) can be used.

**Fig. 27:**
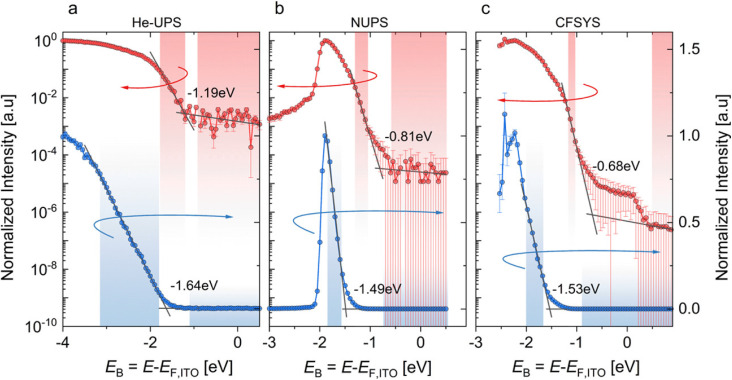
Comparison of different fitting approaches for determining the valence band edge for triple-cation lead-halide perovskite films on glass/ITO substrates. The data are plotted as a function of the binding energy with the Fermi level of the ITO substrate, defining the zero of the energy scale. (a) Satellite-corrected He-UPS, (b) NUPS (using a UV source with 6.5 eV photon energy), and (c) CFSYS measurements. The assignment of the valence band edge was achieved in all cases by obtaining the intersection of a fit to the linear or logarithmic band edge data with the background. Especially in the case of the logarithmic method, the intercept critically depends on the dynamic range of the measurement, which sets the level of the background and, consequently, the position of the intercept. Note the large method-induced differences in the logarithmic determination of the valence band edge, as well as the generally much higher distance *E*_F_ − *E*_V_ obtained for the linear method. Reprinted with permission from ref. 31. © American Chemical Society (2021).

This method poses a range of challenges whenever absolute numbers of these energy levels are required. First, UPS measures energy levels in ultrahigh vacuum (UHV) using a method that is very surface-sensitive (a few monolayers), which implies that it accesses the surface of a film, but not the actual interface of a device. Furthermore, contamination of the sample surface might have affected the results. Second, the determination of the valence band edge is an attempt to assign a number to a continuously changing function. Depending on the exact shape of the data, different evaluation techniques might lead to drastically different results. Fig. 27 illustrates this problem by showing the same data on logarithmic (red) or linear scales (blue) originating from three different variants of photoelectron spectroscopy applied to the same sample.^31^ These are the most commonly seen UPS with a He source (He-UPS), a UPS variant using a near-UV light source (NUPS),^31^ and a variant called constant final state yield spectroscopy (CFSYS),^158^ which offers a particularly high dynamic range. The numbers indicated in the figure are the distance from the Fermi level to the valence band edge (*i.e.* −1.19 eV means that the valence band is 1.19 eV below the Fermi level). We note that for the logarithmic method, we observe variations from *E*_F_ − *E*_V_ = 0.68 eV to 1.19 eV distance, while for the linear method the differences are smaller, but the absolute values are higher, suggesting a strong *n*-type doping of the surface of the film. This strong surface doping is likely an artefact of the linear extrapolation method, which overestimates the distance *E*_F_ − *E*_V_. Thus, different photoelectron spectroscopy methods and different ways of assigning a number (the valence band edge) to a measured spectrum can lead to a range of energy levels of nearly 1 eV, which could be (depending on the band gap of the semiconductor under investigation) the difference between highly n-type or highly p-type. Thus, the interpretation of the data can invert the conclusion by 180° thereby leading to a massive challenge for any quantitative data interpretation. I will therefore come back to this point in the “opportunity” section of this chapter.

In addition to the challenge of quantifying the band edge positions *via* photoelectron spectroscopy, there are also conceptional problems in how to interpret the band edges. If we had a device geometry consisting entirely of crystalline inorganic semiconductors, we could argue that the band edge is well defined as the onset of the square-root-like density of states that we expect to have in 3D crystals. In the case of halide perovskites, however, we often have interfaces between inorganic and organic materials, where the meaning of the offset becomes more difficult to define. Let us take for instance, the interface between an inorganic material with a square-root-like density of states and a molecular semiconductor with a Gaussian density of states, the concept of an offset will critically depend on the characteristic energy that we use for the Gaussian density of states. Here, the onset will be impossible to use as a Gaussian continually decreases towards zero. The only characteristic and well-defined point is the peak.

Furthermore, in addition to the conceptual definition of the offset, also the absolute value of the density of states will have an impact on the quantitative effects seen in the devices. If for instance, the density of states is very high inside the ETL, then the density of electrons will be higher there for a flat Fermi level as if the density was lower. Thus, a higher density of states will effectively function like a lower lying (in the case of electrons) or higher lying (in the case of holes) band edge. Koopmans and Koster have discussed this topic in ref. 128 and propose an effective figure of merit (see eqn (1) in ref. 128) that combines the effects of energetics and density of states into one parameter.

#### 6.1.3 Opportunity

**Fig. 28:**
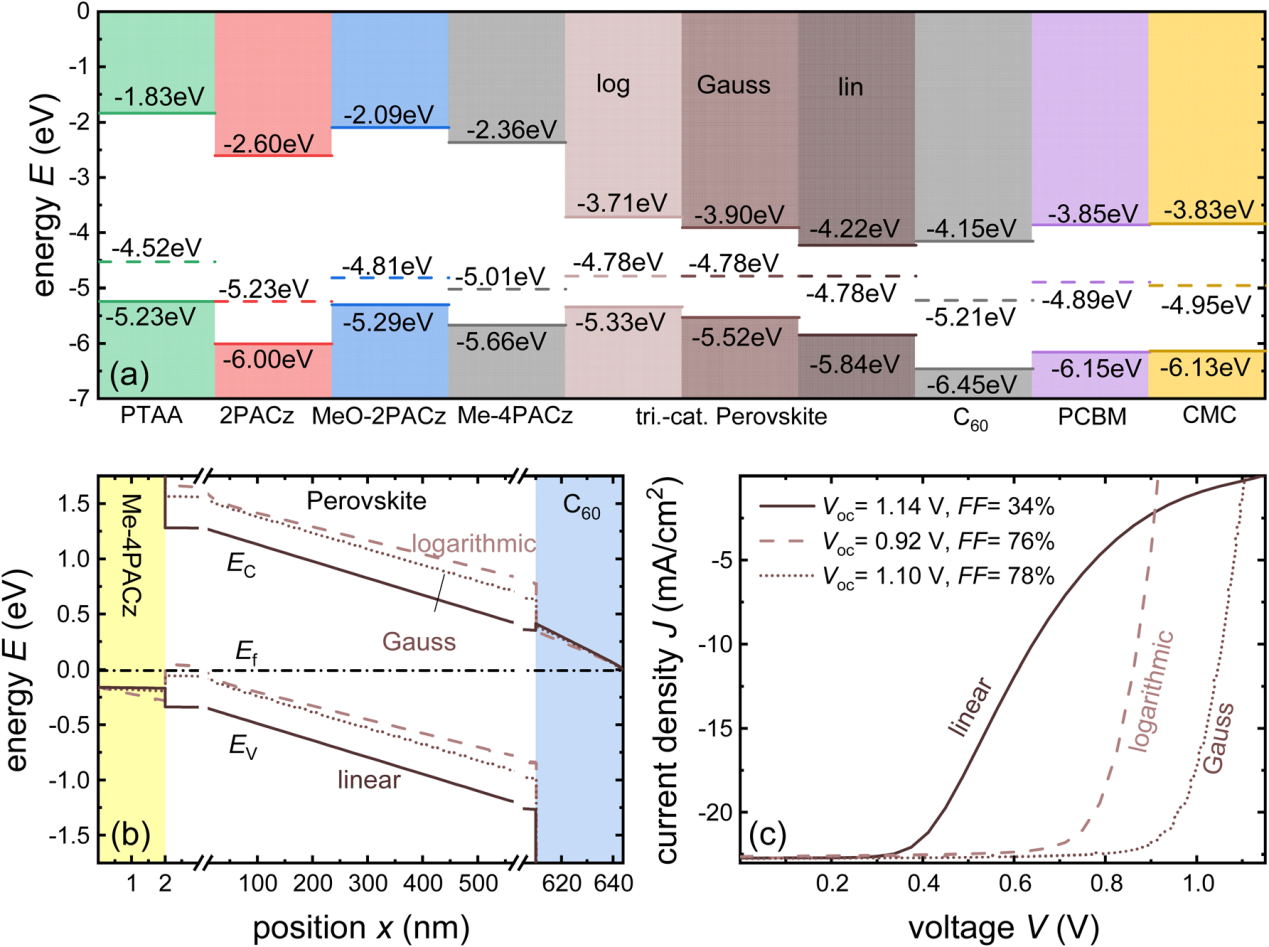
(a) Energy levels (*E*_C_, *E*_F_, *E*_V_ relative to the vacuum level) for different HTLs, perovskite layers deposited on Me-4PACz, and ETLs deposited on perovskite. For all transport layers, the linear method was used to determine the *E*_V_, whereas for the perovskite, we used three methods (linear, logarithmic, and Gaussian fitting). (b) Resulting band diagrams for the stack Me-4PACz/perovskite/C60 and three fitting methods. (c) Simulated current–voltage curves showing S-shapes for the linear method (extraction barrier towards C_60_) and strong losses in open-circuit voltage for the logarithmic method (large band offset towards C_60_). Figure reproduced from ref. 30 under the terms of the CC-BY 4.0 license. © The authors of ref. 30 (2023).

The UPS method, in combination with either IPES and/or optical spectroscopy, has a significant value in determining the relative changes in energy levels. Ref. 159 (see Fig. 5 in this reference) provides a large dataset of different perovskite compositions, where the energy levels were determined using the linear method applied to UPS data. Thus, all energy levels are somewhat further away from vacuum as compared to the literature that uses the logarithmic method or other versions such as Gaussian fitting.^160^ Quantitative device simulations using UPS data as is are, however, extremely difficult, as shown in ref. 30. Fig. 28a illustrates the positions of the conduction and valence band edges for a variety of electron and hole transport layers (determined using the linear method) compared with the three different evaluation methods for the perovskite absorber layer (linear, logarithmic, and Gaussian). Using these numbers, numerical simulations can be performed to test whether a given band diagram (Fig. 28b) is consistent with a functional device (Fig. 28c). In this case, we observe that the linear method with its low-lying energy levels (high electron affinities) has a barrier towards the C_60_ electron extraction layer (see Fig. 28b), which then causes an S-shaped current voltage curve (see Fig. 28c). The logarithmic extraction method leads to a high band offset towards the C_60_ electron extraction layer, which leads to strong losses in the open-circuit voltage. The Gaussian extraction method leads to intermediate values of the energy levels that lead to the best compromise between the offsets and extraction barriers. Thus, for the Gaussian extraction method, realistic *JV* curves can be obtained using realistic parameters for recombination and transport (see ref. 30 for further details on the simulation parameters used). Thus, Fig. 28 shows the difficulty in obtaining absolute values that can be used for modelling and understanding complete devices. Thus, photoelectron spectroscopy could be used in the future in combination with other characterisation methods that are sensitive to the band offsets and positions of the Fermi level. One example is the measurement of the current–voltage curves of single-carrier devices, as described in Section 3.1. These current–voltage curves are sensitive to the built-in voltage of the devices^80^ and, therefore, to the difference in the injection barriers. This can provide some indication of how energy levels change with the type of transport layer material used. Furthermore, tr-PL measurements of bilayers between the perovskite and a transport layer are sensitive to the band offset if the laser fluence is sufficiently high (see Fig. 14). While such data are difficult to analyse quantitatively, they can provide additional insights into the presence or absence of band offsets. Finally, evidence for larger extraction barriers can be obtained from the S-shaped current–voltage curves of the solar cells under illumination. Thus, a simple comparison among the obtained energy levels, experimental device performance, and simulated device performance can yield important insights. The position of the Fermi level can be translated into a doping density, which can then be compared with other doping density measures, such as the series of measurements used by Peña-Camargo *et al.*^42^ in their multi-method study on doping densities in lead halide perovskites.

### 6.2. Electrostatics and ion movement

#### 6.2.1 Introduction

A peculiar property of halide perovskites that distinguishes the material system from other photovoltaic materials is the presence of mobile ions *i.e.* charged defects that can move within the perovskite absorber layer and in some cases also beyond. The flexibility of the perovskite lattice may have positive consequences such as the screening of the Coulomb potential around a defect leading to a rather local influence of the defect on the lattice.^161^ However, there is also ample evidence that the creation of high densities of charged defects and their subsequent movement can lead to performance losses and long-term degradation.^162^ It is therefore somewhat surprising that in most technological papers on high efficiency perovskite solar cells, the characterization of ionic motion and its influence on performance and degradation is hardly discussed. The only canonical technique is to measure the current voltage curve in two scan directions (short circuit to open circuit and *vice versa*) and to note down the scan speed of the measurement (in mV s^−1^).^163^ As we will see in the following, the comparison of forward and reverse scan at a single scan speed is useful only to overlook the effect of ions but not useful if one aims to detect it. Thus, this chapter is another exception to the logic of the review insofar as I will only cover methods that may eventually become canonical but are currently only used in more specialized publications.

#### 6.2.2 Challenge

With ions, even more as with defects, the challenge for the photovoltaics community is to overcome the desire to prove the absence of negative effects caused by mobile ions and thereby to be influenced by confirmation bias. The increasing interest of the scientific community in applications of halide perovskites that make use of ions (*i.e.* in memristors)^164,165^ might therefore be a positive development to arrive at a better understanding of reversible and irreversible degradation in halide perovskites used in solar cells. One of the simplest techniques to quantify the effect of ionic motion on solar cell performance and degradation is the so-called fast hysteresis measurement that involves measuring the current of a solar cell as a function of triangular voltage pulses of different period length.^166,167^ This is equivalent to measuring current voltage curves at a large variety of different scan speeds. However, rather than using source-measure units as for traditional *JV* curve measurement, the fast hysteresis method uses function generators and preamplifiers for generating the voltage ramps and an oscilloscope for detection of the measured current (measured *via* the voltage drop over a resistance). This has the advantage that a wide range of frequencies can be probed but also leads to the minor challenge that the result depends on the resistor over which the oscilloscope measures the voltage and in consequence the current of the solar cell. This resistance is usually varied to generate a good compromise between signal-to-noise ratio of the measured current and minimizing the influence of the series resistance caused by the resistor.

**Fig. 29:**
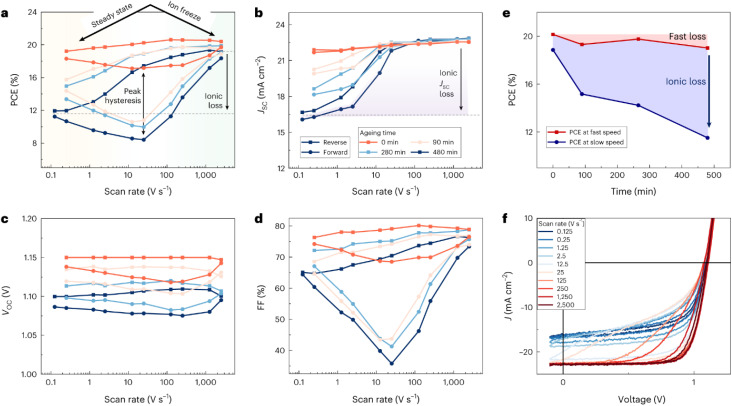
(a) Efficiency (b) *J*_sc_, (c) *V*_oc_, and (d) FF as a function of scan rate and degradation time for a triple-cation perovskite solar cell. (e) Efficiency loss as a function of ageing time, whereby the losses are split up in ionic losses (see panel (a)) and fast losses, *i.e.* the losses at the high scan rate condition. (f) Current–voltage curves after 480 min of ageing as a function of scan rate. Figure reproduced from ref. 162 under the terms of the CC-BY 4.0 license. © The authors of ref. 162 (2024).

Fig. 29 shows an example of such a measurement,^162^ whereby panels (a) to (d) show the photovoltaic parameters ((a) efficiency, (b) *J*_sc_, (c) *V*_oc_, and (d) FF) as a function of scan rate (in V s^−1^) and as a function of aging time (see color of the lines and the legend in panel (b)). We note that at typical scan rates of 100 mV s^−1^ that are used for traditional *JV*-curve measurements, the difference between forward and reverse scan is minimal (left edge of the figures). The same is true for the fastest shown measurements. In an intermediate range of scan rates, however, the difference between forward and reverse scan has a maximum that is primarily caused by a huge discrepancy in FF (see panel (d)). This result is logical if we assume that for very slow scan speeds, the ions can follow the voltage, while for very fast scan speeds they will be fixed at the condition set before the scan started. These are the two situations labelled “steady state” and “ion freeze” in Fig. 29a. At an intermediate scan rate, the ions will move during the scan but will not have found a steady-state configuration, thereby leading to an efficiency that depends strongly on the scan direction (labelled as “peak hysteresis” in panel (a)).

The second observation is about long-term degradation and is summarized in panel (e) which shows that degradation primarily affects the steady state efficiency but less so the efficiency at the ion-freeze condition of fast scan rates. Thus, these results suggest that the major contribution to the degradation is at least related to the mobile ions as it shows up as a growing difference between the high and low scan rate efficiencies (labelled ionic loss in panel (a) and (e)).

The fast hysteresis measurement provides a remarkably simple approach to quantifying the effect of mobile ions on performance and degradation. Challenges associated with this type of measurement are primarily relevant for researchers who aim for more quantitative information on the ions themselves such as their diffusion coefficient and absolute densities. These properties will affect the fast hysteresis measurements, but they may not do so in a sufficiently unique way to be able to infer these parameters with certainty. For instance, Le Corre *et al.*^167^ show how the ion density and the ion diffusion coefficients affect the position and extent of the region of peak hysteresis (see Fig. 1d and e in ref. 167). Thus, additional measurements are likely beneficial to quantify *e.g.* ion densities. Conceivable approaches are to use variants of charge extraction methods such as BACE (bias-assisted charge extraction) or CELIV (charge extraction with linearly increasing voltage)^42,90,167^ that allow quantifying the ion density from the current extracted from the sample at longer times after the voltage step that triggers the charge extraction process. The frequency domain equivalent of these techniques is to quantify ion densities *via* the low frequency capacitance response as shown for instance by Diekmann *et al.*^90^ or by Diethelm *et al.*^91^

#### 6.2.3 Opportunity

There are a wide variety of methods available that provide valuable insights into the effect of mobile ions on performance and degradation. The general appeal of most of these methods is that they have a fairly low activation energy for experimental implementation as they are usually applied to devices and require relatively standard equipment available in many laboratories (such as function generators and oscilloscopes). Thus, characterizing ions has the potential to eventually become part of the standard repertoire of perovskite solar cell characterization. A potential roadblock for adaptation might be less technical but rather embedded in the inner workings of the academic system which provides incentives to show the absence rather than the presence of metastable effects in electronic devices. While stability and metastability caused by ion movement are not the same but likely related phenomena, the editorial^4^ and critique of Prashant Kamat on the community focus on advertising “highly efficient and stable” perovskite solar cells is equally applicable to the topic of metastability.

On the bright side, the topic of metastability is a thankful and popular topic for specialized papers that focus on the phenomena rather than technology development. There are a couple of recent findings that I would like to highlight as an outlook to show possible future developments. The paper by Thiesbrummel *et al.*^162^ already featured before, is significant as it applies a reasonably simple method to relevant samples and shows that the ions are strongly affecting degradation. A somewhat related finding was recently reported by Jiang *et al.*^168^ showing how ions and their interaction with electron and hole transport layers affect degradation under reverse bias stress. A related work by Li *et al.*^169^ showed that the detrimental effects of reverse bias can be mitigated by ion diffusion barriers consisting (in this case) of SnO_2_ layers. Especially, the combination SnO_2_/ITO that is commonly used in tandem solar cells yields significant stability advantages that are consistent with the high reverse bias stability seen in silicon-perovskite tandem solar cells.^170^

In addition to obtaining further insights into the degradation mechanism, there are also methodological innovations within the field. In addition to the example of the fast hysteresis measurement, a somewhat complementary technique recently developed by Hill *et al.*^171^ is the stabilize and pulse experiment. Rather than going through all different scan rates, the focus in this experiment is to put a larger emphasis on the prebias condition (stabilizing phase) and then measure the *JV* curve using a series of fast voltage pulses. To better explain the influence of ions, the combination of these experimental methods with drift-diffusion simulations that explicitly consider ion movement is highly beneficial.^172^

## 7. Outlook and conclusions

The state of the research community working on the device physics of halide perovskites can be best understood by comparison with crystalline silicon. In the case of silicon, many material properties have been determined over the years, which implies that sophisticated parameterisations such as mobility^173^ or Auger coefficients as functions of doping density^174^ or (in the latter case) excess carrier density exist and can be used in simulation software.^173^ In the world of crystalline Si, relevant variations can include the type of wafer (for example, Czochralski *vs.* floatzone), the doping type and density, and the surface texture and passivation. Often, surface passivation has the most significant influence on the charge carrier lifetimes, while the mobilities are largely known. There wouldn't even be a need to experimentally determine them anymore if the doping density is known. Wafer characterization for the purpose of solar cell development can therefore focus on the measurement of minority carrier lifetimes using *e.g.* the quasi-steady-state photoconductivity method^175^ possibly combined with optical characterization of the passivation and transport layers to quantify possible parasitic absorption losses.^176^

In the world of halide perovskite research, it is often not even clear what kind of model one would have to use for a given simulation or attempt to data extraction.^23^ Furthermore, the huge variety of different compositions, different surface passivations and treatments as well as different characterisation methods lead to the unfortunate situation that even for a given sample, there is rarely such a thing as a well-defined parameter such as lifetime or mobility. However, it is possible to measure samples using different techniques, extract parameters from the observables, and then compare parameters obtained from different measurements to approach a consistent model and a consistent set of parameters that can describe the behaviour of the solar cell and can help in designing better solar cells.^33,177,178^ The development of measurements or variants of measurements that provide additional degrees of freedom is therefore important, as they will provide more measurement data to quantify the many unknowns that exist.^179^ Examples of additional degrees of freedom that have recently been explored are the different implementations of photo-Hall measurements,^145,146^ as well as variants of transient photoluminescence employing variations of repetition rate^23^ or the use of pulse bursts^180,181^ which are not commonly employed in the literature.

The article argues that one of the major obstacles towards this goal of determining a consistent set of parameters and using a consistent model is the step from the experimental observables (PL intensity, current, impedance, *etc*…) to the parameters (mobility, lifetime, surface recombination velocity, *etc*…).^179^ This step requires either an analytical or numerical approach. As most analytical equations are valid only within a limited parameter range,^29,89,182^ the sensible choice is often the numerical approach to data analysis.^40,111,183^ However, this approach has the decisive disadvantage that data analysis might become a chicken- and egg-type problem as one parameter will depend on another. Eventually, the problem will boil down to the determination of several unknowns using several experimental datasets and a global model that should describe all experimental datasets simultaneously. Such approaches have been applied to halide perovskite research in the past,^33,177,178^ but examples are rather scarce, given the relatively large effort associated with such global fits. Thus, future research should focus on data analysis methods that significantly speed up the analysis of several experimental datasets using a global model with several unknowns. Approaches employing the tool set of Bayesian statistics and Bayesian parameter inference exist,^41,184^ and first approaches to apply them to halide perovskites have been published.^40,111,179^ However, this field is currently still in its infancy. The rapid developments in the general field of machine learning methods provide some hope that data analysis methods based on sensible numerical models will become more usable and more widely applied within the community and may be able to overcome the current tendency to use overly simplified analytical models.

Beyond the prospect of better data analysis methods to be implemented in the future, this article provides a summary of the major challenges that researchers face when applying different methods and possibilities to overcome or mitigate the challenges that can be implemented without the need for additional developments. Table 3 goes back to the discussion in the introduction and provides a new column as compared to Table 1 that summarises the opportunities and safeguards that are recommended to either verify the validity of certain data analysis approaches (*e.g.* the case of SCLC or capacitance measurements), provide alternative analytical equations (*e.g.* the case of transient photovoltage or photoluminescence), suggest less common variants of measurements to obtain additional information (see the use of the peak shift in transient photoluminescence), or suggest specific combinations of measurements that are not commonly used in the literature to verify or falsify certain conclusions (see, *e.g.* the chapter on photoelectron spectroscopy). Many, but certainly not all, of the methods discussed here tend to be on the less expensive end of equipment costs such that their application is or could be rather widespread.

**Table 3:** List of electronic properties of interest for perovskite photovoltaics connected to typically used characterisation methods and associated opportunities to overcome the risks highlighted in Table 1 and to improve data analysis and correct interpretation of experimental data

Quantity	Method	Opportunities and safeguards
Defect density	Trap filled current in single carrier devices	Measure built-in voltage using all four branches of the *JV*-curve (up, down, forward, reverse)^30,80^
	Capacitance based methods	Consider detection threshold,^72^ study lateral devices^121^ or thick devices^86^
Lifetime	Transient photoluminescence	Determine recombination coefficient from power law shaped decays^25^
		Use low repetition rates. Vary repetition rates or use complex pulse sequences^23,180^
	Transient photovoltage	Combine rise and decay of photovoltage^17^
	Impedance	Vary voltage or light intensity. Use matrix model^27^ for data analysis
Doping	Capacitance, Mott–Schottky	Consider detection threshold.^72^ Include ionic contributions in numerical simulations^91^
Energy levels	Photoelectron spectroscopy	Complement with device simulation as well as with complementary methods such as SCLC^30^
Mobility	SCLC, TPC	Remove contact layer or account for contact layers in data analysis^30^
	Photoluminescence	Exploit peak shift to determine out-of-plane mobilities^32^
Charge transfer and extraction	Transient PL on bilayers	Vary laser fluence. Use numerical methods for data analysis^34^
	Voltage dependent PL	Vary properties of ETL or HTL to isolate the extraction-limiting interface^104,126,127^

**Fig. 30:**
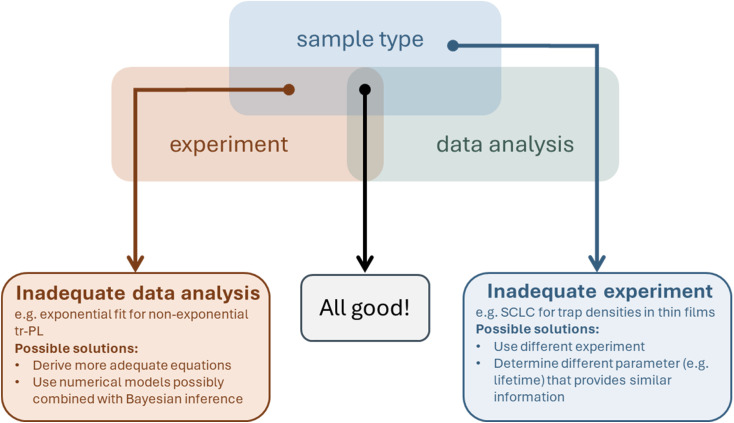
A good experiment requires a match between the sample and its properties (*e.g.* thickness, presence of ions, low doping density), the experiment, and the data analysis (analytical, numerical). Many problems within the field of halide perovskites can be summarized by a mismatch between these three, whereby the typical problems are that an inadequate data analysis is used that does not consider the specific properties of halide perovskites or that an altogether inadequate experiment is used that is not sensitive to the desired material parameters. The solutions and opportunities discussed in this perspective therefore aim to provide solutions to either of these challenges.

Finally, Fig. 30 aims to summarize the main message of the study in a more abstract manner. Meaningful characterization of samples within any research field requires a good match between the sample, the experiment and the data analysis method. The experiment and the data analysis approach must consider the properties of the sample. In the case of halide perovskites, parameters and properties are so difficult to classical semiconductors such as silicon that often either the experiment or the data analysis method are inapplicable to the sample. Thus, solutions as presented in this article will always provide either more adequate experimental methods or variants of existing methods or analysis methods that are better adapted to the expected sample properties.

## Data availability

No new data is associated with this work.

## Author contributions

TK wrote the article.

## Conflicts of interest

The author declares no competing financial or non-financial interests.
